# Spectrum and functions of ion channels and transporters in osteoclasts

**DOI:** 10.1038/s41413-026-00513-9

**Published:** 2026-03-27

**Authors:** Hongyu Chen, Yanli Zhang, Yulong Zhu, Xiang Xiao, Shanshan Huang, Xiaohong Duan

**Affiliations:** State Key Laboratory of Oral & Maxillofacial Reconstruction and Regeneration, National Clinical Research Center for Oral Diseases, Shaanxi Key Laboratory of Stomatology, Department of Oral Biology, Clinic of Oral Rare Diseases and Genetic Diseases, School of Stomatology, The Fourth Military Medical University, Xi’an, China

**Keywords:** Bone, Bone quality and biomechanics

## Abstract

Osteoclasts are essential for bone resorption and interact with osteoblasts during bone remodeling. Ion channels and transporters located in the ruffled border or intracellular vesicles coordinate the transport of various ions and substrates, which is fundamental to the primary functions of osteoclasts. Numerous channels and transporters are implicated in bone metabolic disorders and genetic diseases. Among these, the voltage-gated chloride channel 7 (ClC-7) and vacuolar proton ATPases (V-ATPase) represent the most well-characterized examples in osteoclasts. Using the classification system of the Transporter Classification Database, we reviewed nearly 90 osteoclastic ion channels and transporters, categorizing them into six groups: ATPases, cation channels, anion channels, complex transporters, organic substance transporters, and ATP-binding cassette transporters. We summarized recent advances in their subcellular localization, transported substrates, associated diseases, and physiological roles in relevant biological functions and signaling pathways. Notably, transporters for hydrogen, chloride, phosphate, and calcium are particularly critical for osteoclast function. We also reviewed therapeutic candidates targeting these ion channels and discussed strategies for their future development. As transcriptome and other advanced techniques have identified more channels and transporters in osteoclasts, the diversity and unexplored functions of these molecules may exceed previous understanding. Increased attention to their widespread distributions and interactions could reveal new therapeutic targets for osteoclast-related and other bone disorders.

## Introduction

Osteoclasts are essential for bone resorption and help bone remodeling through their interactions with osteoblasts. Active ion transport across the osteoclast membrane is critical for bone resorption and other cellular functions.^1,2^ Over recent decades, numerous ion channels and transporters have been identified as key regulators of osteoclast ion homeostasis, including subunits of vacuolar proton ATPases (V-ATPase) and the voltage-gated chloride channel 7 (ClC-7)^3–6^. Dysfunctions in these proteins are associated with various bone disorders, such as osteoporosis and osteopetrosis, as well as certain systemic diseases.

Advances in RNA sequencing and related technologies have broadened our understanding of the diverse repertoire of ion channels and transporters in osteoclasts. However, the precise localization and functional roles of many of these proteins remain incompletely characterized. According to our incomplete statistics, although over 600 genes of ion channels have been investigated in previous literature, only about 90 of them have been identified and studied in osteoclasts. In this review, we summarize recent progress in the study of ion channels and transporters in osteoclasts. We classify these ion channels and transporters according to the Transporter Classification Database, focusing on six major groups: ATPases, cation channels/transporters, anion channels/transporters, complex transporters (cotransporters, symporters, or antiporters), organic substance transporters, and ABC (ATP-binding cassette) transporters. By systematically organizing this information, we aim to provide a comprehensive perspective on the functional roles and ion transport mechanisms in osteoclasts, offering new insights to guide future research.

## Part I ATPase

### Proton-transporting ATPase

An acidic microenvironment within the bone resorption pit is essential for proteolytic enzymes to degrade the bone matrix. Osteoclasts secrete substantial quantities of protons (H^+^) into the resorption pit via proton pumps, exchangers, and voltage-gated H^+^ channels. These transporters facilitate proton secretion and maintain intracellular acid-base balance. Additionally, they regulate osteoclast activity, proliferation, differentiation, and apoptosis, playing a critical role in bone metabolism homeostasis.^3–5,7^ Voltage-gated H^+^ channels will be discussed in detail in a subsequent section.

**Fig. 1:**
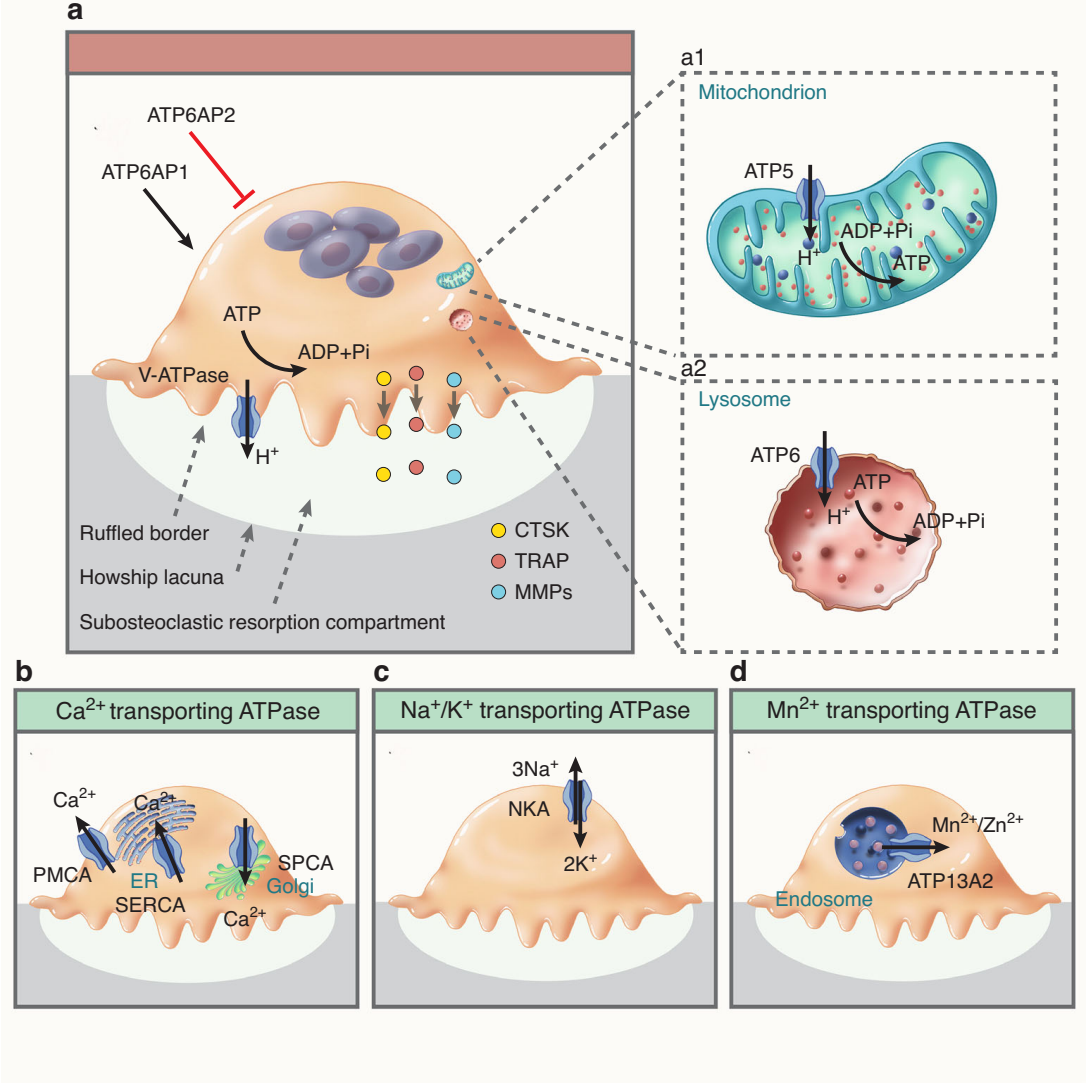
Schematic diagram of various ATPases in osteoclasts. **a** H^+^ transporting ATPase. V-ATPase pumps proton into the resorption lacuna to form an acidic microenvironment for bone matrix degradation. The ATP5 family in mitochondria participates in ATP synthesis (**a1**), while the ATP6 family in lysosomes mediates lysosomal acidification (**a2**). **b** Ca^2+^ transporting ATPase. The plasma membrane Ca^2+^-ATPase (PMCA), sarco/endoplasmic reticulum Ca^2+^-ATPase (SERCA), and secretory pathway Ca^2+^-ATPase (SPCA) are localized at the plasma membrane, endoplasmic reticulum (ER), and Golgi apparatus, respectively. They are involved in Ca^2+^ transport across cell membranes and between organelles, regulating osteoclast differentiation and function. **c** Na^+^/K^+^ transporting ATPase (NKA). It actively transports three Na^+^ out of the cell and two K^+^ into the cell by hydrolyzing ATP, maintaining cellular ion gradients and osmotic balance. **d** Mn^2+^ transporting ATPase (ATP13A2). Localized in endosomes, it is involved in the transport of metal ions such as Mn^2+^ and Zn^2+^, maintaining intracellular metal ion homeostasis. Other labeled molecules include CTSK (Cathepsin K), TRAP (Tartrate-resistant acid phosphatase) and MMPs (Matrix metalloproteinases)

Hydrogen ion-transporting ATPase is a complex consisting of multiple transmembrane proteins that form membrane pump structures responsible for ATP hydrolysis and hydrogen ion extrusion across the cell membrane.^7^ There are two principal types of proton pumps: F-type ATPase found in bacteria and plant cells, and V-type ATPase present in animal cells and certain bacteria. The mitochonrial H^+^-transporting ATPase is encoded by the ATP5 family, while the lysosomal H^+^-transporting ATPase is encoded by the ATP6 family.^8,9^ Notably, subunits from both the ATP5 and ATP6 families are detected in osteoclasts at high expression levels. We have previously reviewed various subunits of V-ATPases in osteoclasts.^10^ These molecules play essential roles in regulating extracellular acidification,^11^ maintaining intracellular compartment pH homeostasis,^12,13^ facilitating cytoskeletal F-actin assembly,^14^ promoting preosteoclast fusion,^15,16^ mediating endocytosis and vesicle trafficking,^17^ processing and degrading endoplasmic reticulum proteins,^18^ and modulating enzyme activity^19^ (Fig. 1).

#### ATPase, H^+^ transporting, lysosomal

The mammalian V-ATPase proton pump comprises a peripheral V1 domain and a membrane-bound V0 domain. The V1 complex consists of subunits A through H, while the V0 complex contains subunits a through e.^9^ In osteoclasts, V-ATPase complexes incorporating subunits a3, d2, and C1 localize specifically to ruffled borders, where they maintain the acidic extracellular microenvironment essential for bone resorption.^20^ Notably, subunit a3 plays a pivotal role in regulating acidification of the bone resorptive lacunae.^20^ The V-ATPase subunit ATP6AP1 (Ac45) critically supports multiple osteoclast functions, including differentiation, extracellular acidification, lysosomal trafficking, and protease exocytosis, all vital processes for osteoclast-mediated bone resorption.^21^ The impairment of ATP6AP1 function reduces osteoclast numbers by diminishing the proliferation and fusion of their precursors, owing to lower extracellular signal-regulated kinase (ERK) phosphorylation and downregulated expression of differentiation factors such as FBJ osteosarcoma oncogene (c-fos), nuclear factor of activated T-cells, cytoplasmic 1 (NFATc1), and transmembrane 7 superfamily member 4 (Tm7sf4, also known as DC-STAMP) (ref. ^21^). Unlike ATP6AP1, which promotes osteoclast formation, ATP6AP2 acts as an inhibitor of both osteoclastogenesis and osteoclast activity. Current evidence suggests that ATP6AP2 maintains β-catenin levels, thereby preventing excessive osteoclast activation and subsequent trabecular bone loss.^22^

*Atp6v0c* has been identified as a candidate gene for the rat osteopetrosis phenotype^23^ is also implicated in neurodevelopmental disorders frequently associated with epilepsy.^24^ In mice, *Atp6v0d2* shows high expression levels in mature osteoclasts, and its depletion completely inhibits extracellular acidification.^16^ *Atp6v0d2*-deficient mice exhibit increased bone mass.^25^ Studies indicate that *Atp6v1a* and *Tcirg1* mRNA expression in osteoclasts correlates with fluid shear stress.^26^ The B2 subunit (but not the B1 isoform) localizes to intracellular vesicles and ruffled membranes of osteoclasts.^27,28^ Phosphatidylinositol 3-kinase (PI 3-kinase) critically mediates the interaction between the B2 subunit and F-actin, which is essential for recruiting V-ATPase complexes to osteoclast ruffled borders during polarization and bone resorption.^29^ The C1 subunit shows significant expression in osteoclasts and interacts with the ruffled border-localized a3 subunit. C1 deficiency severely impairs osteoclast acidification and bone resorption capacity.^30^ Lentiviral-shRNA-mediated knockdown of *Atp6v1c1* in 4T1 mouse models reduced tumor-induced bone resorption and metastasis in vivo by modulating osteoclastogenesis; in addition, it impaired V-ATPase activity, cell proliferation, and mTORC1 activation in vitro.^31^ Bivariate genome-wide association studies have identified ATP6V1G1 as a novel pleiotropic locus associated with both osteoporosis and age at menarche.^32^ In osteoclasts, ATP6V1H localization has been confirmed, and its deficiency leads to increased intracellular pH. This results in inhibited osteoclast formation and function, including impaired bone resorption capacity and reduced induction of osteoblast formation.^33^ Studies using CRISPR/Cas9-generated *Atp6v1h*^*+/-*^ mice revealed decreased bone remodeling and net bone matrix loss. Osteoclasts from these mice exhibited impaired bone formation and resorption activity. The observed elevation in intracellular pH within *Atp6v1h*^*+/-*^ osteoclasts was shown to downregulate TGF-Β1 activation, consequently reducing osteoblast formation induction.^33^ Further investigations demonstrate that Atp6v1h deficiency affects multiple physiological processes: it impairs glucose tolerance by enhancing endoplasmic reticulum stress in high-fat diet-fed mice, while also preventing bone loss in simulated microgravity conditions through the fos-jun-src-integrin pathway.^34,35^

#### ATPase, H^+^ transporting, mitochondrial

Mitochondrial ATP production serves as the primary energy source for intracellular metabolic pathways. This process synthesizes ATP from ADP in the mitochondrial matrix, utilizing the proton electrochemical gradient as the driving force.^36^ The F_0_-F_1_ ATP synthase complex mediates mitochondrial ATP synthesis through two structural components: (1) the F_0_ proton channel, composed of a, b, and c subunits, and (2) the F_1_ catalytic moiety containing α, β, γ, δ and ε subunits.^37^ In osteoclast biology, *ATP5B* encodes the β subunit of the F_1_ ATP synthase complex, which constitutes the catalytic core responsible for ATP production.^38^ During osteoclast differentiation from myeloid monocytic precursors, ATP5B expression shows significant upregulation compared to other F_0_-F_1_ ATPase subunits. Functional studies demonstrate that ATP5B inhibition specifically impairs both osteoclast differentiation and bone resorption capacity.^38^

### Calcium-transporting ATPase

Calcium-transporting ATPase, commonly referred to as calcium ATPase (Ca^2+^-ATPase), transports calcium ions across cellular membranes. Depending on intracellular calcium ion concentration, it moves extracellular calcium into the cell or exports intracellular calcium out of the cell, a process driven by ATP hydrolysis. Beyond regulating calcium ion concentration, Ca^2+^-ATPase participates in other biological processes, including intracellular pH modulation and protein synthesis.^39,40^ Structurally, Ca^2+^-ATPase functions as a multi-subunit enzyme complex composed of α, β, γ, δ, and ε subunits. The α subunit serves as the catalytic center, the β subunit contains the ATP-binding site, and the γ subunit facilitates ATP hydrolysis.^41^ The coordinated interaction of these subunits enables Ca^2+^-ATPase to mediate calcium ion transport. Based on subcellular localization, Ca^2+^-ATPase is categorized into three subtypes: sarco/endoplasmic reticulum Ca^2+^-ATPase (SERCA), encoded by *ATP2A1-3*; plasma membrane Ca^2+^-ATPase (PMCA), encoded by *ATP2B1-4*; and secretory pathway Ca^2+^-ATPase (SPCA), localized in the Golgi apparatus and Golgi-derived vesicles and encoded by *ATP2C1*, *ATP2C2*.^41^

The precise regulation of Ca^2+^ dynamics is essential for the proper differentiation and function of osteoclasts. In *Serca2*^*+/−*^ (*Atp2a2*) bone marrow monocytes, RANKL treatment induced incomplete formation of multinucleated cells, subsequently leading to diminished bone resorption activity. These findings demonstrate that SERCA2 activation by RANKL is crucial for generating RANKL-induced intracellular calcium (iCa^2+^) oscillations, which are vital for osteoclastogenesis.^42^ Furthermore, the Sigma-1 receptor suppresses osteoclastogenesis by promoting endoplasmic reticulum-associated degradation of SERCA2.^43^ Thapsigargin, a SERCA inhibitor, reduces both osteoclast survival and bone resorption,^44^ supporting the role of SERCA in regulating these processes.

Emerging evidence suggests that PMCAs play crucial roles in regulating bone homeostasis by modulating Ca^2+^ signaling in osteoclasts.^45^ Among PMCA isoforms, PMCA1 (ATP2B1) and PMCA4 (ATP2B4) have been specifically implicated in osteoclastogenesis. In immature/undifferentiated cells, PMCAs inhibit RANKL-induced Ca^2+^ oscillations and osteoclast differentiation in vitro. PMCA4 exerts additional anti-osteoclastogenic effects by suppressing nitric oxide (NO) production, thereby inhibiting preosteoclast fusion. In addition to their role in immature cells, elevated PMCA expression in mature osteoclasts was found to prevent osteoclast apoptosis in both in vitro and in vivo settings. Genetic studies revealed that *Pmca1* heterozygous and *Pmca4* null mice developed an osteogenic phenotype characterized by increased osteoclast presence on bone surfaces.^45^ Furthermore, *PMCA*-mediated regulation of bone mineralization has been demonstrated in zebrafish models.^46^ These findings position PMCA inhibitors and modulators as potential therapeutic interventions for calcium dysregulation disorders including neurodegeneration,^47^ cardiovascular disorders,^48^ and malignancies.^49^ While the role of the SPCA family in osteoclast biology remains poorly characterized, current evidence supports SERCA and PMCA as promising therapeutic targets for osteoporosis management.

### Sodium/potassium transporting ATPase

The sodium/potassium-transporting ATPase (Na^+^/K^+^-ATPase, NKA) is an essential membrane protein that utilizes ATP hydrolysis to actively transport Na^+^ out of the cell and K^+^ into the cell against their respective concentration gradients. The primary role of NKA is to regulate the concentration gradients of K^+^ and Na^+^ across the cell membrane and to maintain osmotic balance between intracellular and extracellular fluids.^50^ Structurally, NKA forms an oligomeric complex consisting of: a catalytic α subunit (ATP1A) responsible for ion transport; a β subunit (ATP1B) functioning as a molecular chaperone; and a regulatory FXYD protein that modifies transport properties.^51^

Cells depend on NKA activity to maintain ionic, osmotic and electrical homeostasis. Notably, the osteoclast plasma membrane exhibits particularly high NKA expression compared to other bone cells, bone marrow cells, monocytes and macrophages. NKA plays a specialized role in bone resorption through its coupling with the secondary active transport of calcium and/or protons.^52^ The DR-Ab (targeting the DR peptide, a conserved NKA sequence) showed no significant effect on the osteoclast precursor proliferation, but counteracted the inhibitory effect of H_2_O_2_ on osteogenic marker gene expression, including Runt-related transcription factor 2 (*Runx2*), alkaline phosphatase (*Alp*) and bone sialoprotein (*Bsp*).^53^ Importantly, DR-Ab prevented bone loss in an ovariectomised rat osteoporosis model,^53^ underscoring NKA’s potential as a therapeutic target for osteoporosis. FXYD proteins, which regulate NKA activity, are also implicated in bone and pain biology. Fxyd5 can activate the nuclear factor kappa-B (NF-κB) pathway and participates in chondrocyte inflammation and extracellular matrix degradation.^54^ While NKA has been clearly linked to bone resorption function, the specific roles of its individual subunits (α, β and FXYD) in osteoclasts remain unclear, warranting further investigation for potential osteoporosis applications.

### Manganese-transporting ATPase

Manganese transporter genes are classified within the ATP13A family, which comprises five members (*ATP13A1-5*) in humans.^55^ Manganese (Mn), an essential biological cofactor for numerous enzymatic reactions, plays critical roles in various physiological processes. Manganese transporters that remove excess intracellular manganese mitigate cytotoxicity, thereby preserving manganese’s efficacy in essential cellular processes such as enzymatic catalysis.^56^ Research has revealed that ~40% of the body’s Mn is stored in bone, suggesting bone tissue as a potential cumulative biomarker for Mn exposure.^57^ In bone formation processes, osteoclasts particularly depend on manganese transporters for Mn regulation. Previous studies demonstrate that Mn can enhance bone regeneration, with Mn supplementation showing potential for osteoporosis treatment.^58^ Mn^2+^ promotes osteoblast adhesion, viability and proliferation through integrin activation.^59,60^ Interestingly, zinc-regulated transporters (ZNTs) also participate in Mn^2+^ transport, which will be discussed further in the zinc transporters section.^61^

ATP13A1 is essential for mitochondrial antiviral signaling protein (MAVS)-mediated antiviral innate immunity and modulates major histocompatibility complex I-related protein 1 (MR1)-mediated antigen presentation.^62,63^ Mn^2+^ can upregulate the expression of ATP13A2, which helps to protect cells from Mn-induced cytotoxicity by regulating manganese homeostasis.^64,65^ In mammalian cells, ATP13A2 predominantly localizes to lysosomes, the endoplasmic reticulum, endosomes, and multivesicular bodies.^66^ ATP13A2 participates in intracellular metal ion transport and other processes, contributing to the maintenance of intracellular homeostasis. Current studies concerning ATP13A2/ATP13A3 in osteoclasts are lacking. ATP13A2 may affect the metal ion balance, particularly calcium ions, in osteoclasts, consequently influencing their differentiation, activity, and bone resorption function.

## Part II cation transporter

### Proton channel (Fig. 2)

Among the reported proton channels or transporters in osteoclasts, voltage-gated H^+^ channels represent the primary conductive pathways for H^+^^67–69^. The hydrogen voltage-gated channel 1 (HVCN1, also known as HV1 and VSOP) is the sole mammalian voltage-gated proton channel identified in human cells.^70^ The human *HVCN1* gene encodes two isoforms: a full-length 273-amino acid protein and a shorter variant lacking the first 20 amino acids.^71^ HVCN1 functions effectively in the plasma membrane by facilitating dynamin-dependent endocytosis. Furthermore, indirect evidence suggests the presence of proton channels in phagosomes and the Golgi complex.^72^ HVCN1 mediates highly selective outward H^+^ currents, thereby compensating for charge displacement.^73^ Its activity may vary during osteoclastogenesis, serving as a critical mechanism for pH homeostasis in osteoclasts, which encounter significant pH fluctuations during the bone resorption cycle.

**Fig. 2:**
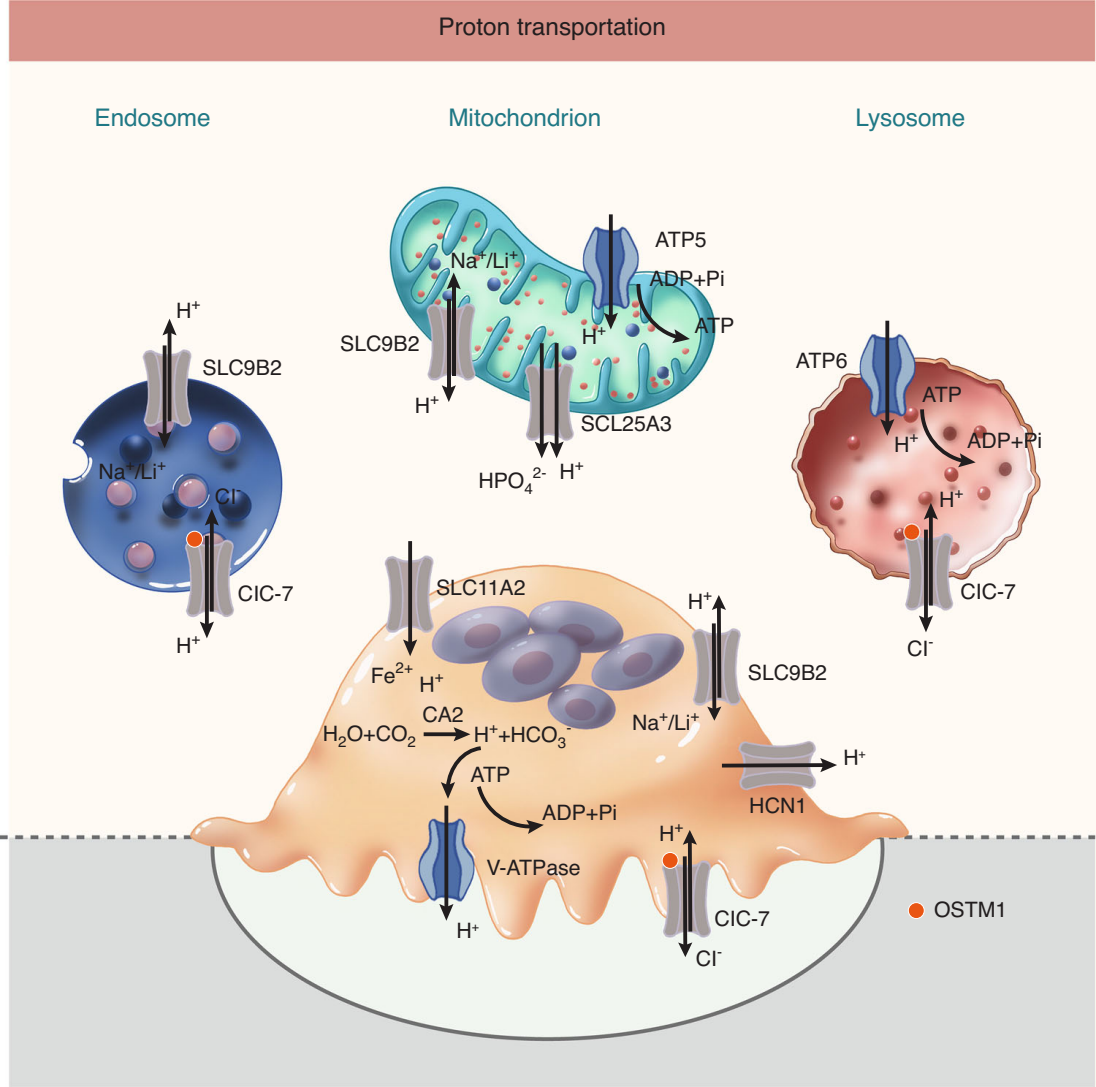
Localization and Function of Proton Transporter in Osteoclasts. Key molecules and their primary roles in establishing the acidic resorption lacuna and maintaining cellular pH balance. Carbonic Anhydrase II (CA2): Cytosolic generation of H^+^ and HCO_3_^−^ substrates. V-ATPase: Localized to the ruffled border for active H^+^ extrusion into the resorption lacuna. HCN1 (Voltage-gated H^+^ Channel): Plasma membrane channel for charge-compensating H^+^ efflux. ClC-7/OSTM1 Antiporter: Ruffled border and lysosomal Cl^−^/H^+^ exchange for electroneutral acid secretion. SLC9B2 (Na^+^/H^+^ Exchanger): Plasma membrane and mitochondrial regulator of cytosolic and organellar pH. SLC11A2 (Iron Transporter): Plasma membrane Fe²^+^/H^+^ cotransport. SLC25A3 (Mitochondrial Phosphate Carrier): H^+^-coupled phosphate import for ATP synthesis. ATP5 (Mitochondrial ATP Synthase): H^+^-driven ATP production. ATP6 (Lysosomal V-ATPase): H^+^ pumping for lysosomal acidification

### Calcium channel

**Fig. 3:**
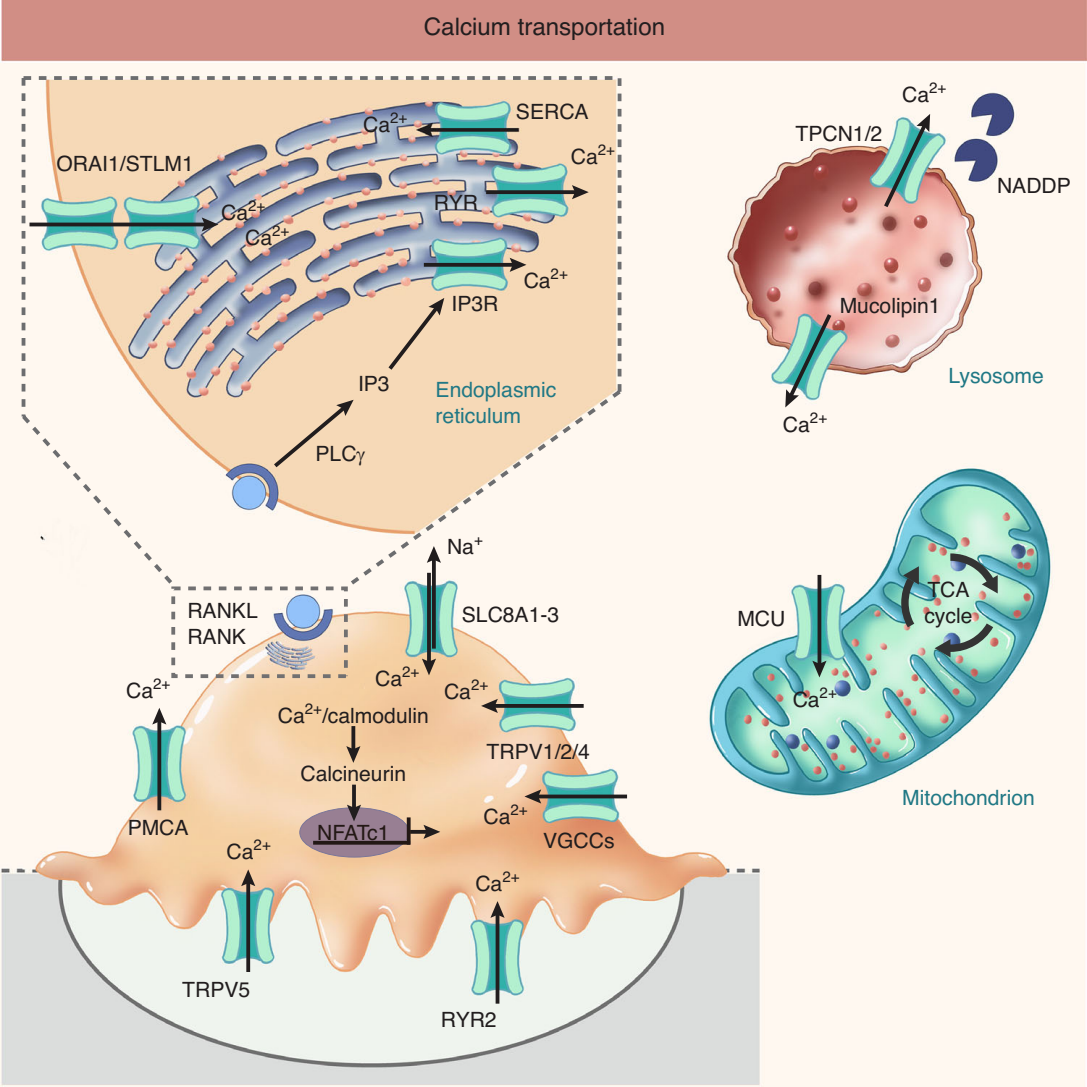
Calcium Transporter Localization and Function in Osteoclasts. Key calcium transporters and channels, categorized by functional class, with their roles in Ca²^+^ signaling, differentiation, and bone resorption. 1. Ca²^+^ Export & Sequestration. PMCA: Plasma membrane Ca²^+^ extrusion for intracellular Ca²^+^ homeostasis. SERCA: Endoplasmic reticulum (ER) Ca²^+^ uptake for ER store maintenance. 2. Ca²^+^ Release from Intracellular Stores. IP₃R_S_ & RYRs: ER membrane receptors for Ca²^+^ release, initiating cytosolic Ca²^+^ oscillations. TPCN1/2 & TRPML1: Lysosomal Ca²^+^ release channels for lysosomal function and autophagy regulation. 3. Ca²^+^ Influx from Extracellular Space. VGCCs: Voltage-dependent Ca²^+^ entry for cytoskeletal reorganization and resorptive activity. CRAC Channels (ORAI1/STIM1): Store-operated Ca²^+^ entry (SOCE) for NFATc1 activation and osteoclastogenesis. 4. Other Key Ca²^+^ Regulators. TRPV Channels: TRPV1/2/4 for general Ca²^+^ signaling; TRPV5 at ruffled border for bone resorption modulation. Mitochondrial Calcium Uniporter (MCU): Mitochondrial Ca²^+^ import for energy metabolism and Ca²^+^ signaling. 5. RANKL-Induced Signaling Cascade. PLCγ activation leads to IP₃ generation, triggering ER Ca²^+^ release, calcineurin activation, and subsequent NFATc1 dephosphorylation to drive osteoclast differentiation

Calcium channels play crucial roles in regulating diverse cellular functions, including membrane depolarization and signaling pathways, enabling calcium to act as a key intracellular messenger in physiological processes such as exocytosis and proliferation.^74,75^ Calcium is particularly important in modulating osteoclast differentiation and function. Calcium oscillations are well-documented in receptor activator of NF-κB ligand RANKL-induced osteoclastogenesis and bone resorption, mediated via the calcineurin pathway.^74–78^ Based on their gating mechanisms, calcium channels can be categorized into voltage-gated calcium channels, ligand-gated calcium channels, and calcium-transporting ATPases (previously discussed in Part I. 2). In this review, we examine calcium channels in osteoclasts according to these classifications, as well as the molecules implicated in mitochondrial calcium uptake (Fig. 3).

#### Voltage-gated calcium channel

Voltage-gated calcium channels comprise multiple subunits (α1, α2δ, β1-4 and γ), with the α1 subunit determining calcium ion flux directionality while the auxiliary subunits regulate channel function.^77^ Based on α1 subunit heterogeneity, these channels are categorized into five principal groups containing at least ten members: L-type (Cav1.1-Cav1.4), P/Q-type (Cav2.1), N-type (Cav2.2), R-type (Cav2.3) and T-type calcium channels (Cav3.1-Cav3.3)(ref. ^77^).

Voltage-gated calcium channels in osteoclasts have been well documented in previous studies.^79^ These channels undergo inactivation upon osteoclast attachment to bone surfaces, a process concomitant with podosome reduction. This suggests their potential role in regulating cytoskeletal reorganization and cell adhesion through intracellular calcium (iCa^2+^) modulation, ultimately affecting bone resorptive activity.^79^ Potassium ion-induced depolarization in osteoclasts transiently elevates cytosolic calcium concentration (iCa^2+^), an effect inhibited by nicardipine, indicating the presence of functional L-type voltage-sensitive calcium channels.^79^ Supporting this, L-type Ca^2+^ channel agonists suppress RANKL-induced osteoclast formation through NFATc1 downregulation.^80^ Notably, Cav1.3 expression is upregulated in rat osteoporosis models and promotes osteoclast differentiation in RAW264.7 pre-osteoclast cell lines.^81^ Furthermore, ionomycin ameliorates hypophosphatasia by restoring L-type Ca^2+^ channel internalization in alkaline phosphatase-deficient mesenchymal stem cells.^82^ However, alternative studies demonstrate that extracellular calcium-induced calcium influx occurs independently of membrane voltage, showing insensitivity to nifedipine, BAYK8644 and diltiazem.^83^ These findings suggest voltage-gated calcium channels may not constitute the predominant calcium transport pathway in osteoclasts.

#### Ligand-gated calcium channel

Ligand-gated calcium channels comprise inositol 1,4,5-trisphosphate receptors (IP₃Rs), ryanodine receptors (RyRs), two-pore channels (TPCs), calcium release-activated calcium (CRAC) channels, and the transient receptor potential (TRP) channel superfamily. These channels predominantly exhibit dual ionic permeability, conducting both calcium ions and other non-selective cations, with TRP channels demonstrating particularly broad cation selectivity.^84–87^

##### IP3 receptor, ryanodine receptor, two-pore channel

IP₃ receptors (IP₃Rs) and ryanodine receptors (RyRs) represent the canonical ligand-gated calcium channels. These evolutionarily related channels are activated by cytoplasmic Ca²^+^ to mobilize calcium from intracellular stores. While IP₃Rs respond to inositol 1,4,5-trisphosphate (IP₃), RyRs are activated by cyclic adenosine diphosphate ribose (cADPR), with both channel types collectively regulating Ca²^+^ release from the endoplasmic/sarcoplasmic reticulum (ER/SR). Nicotinic acid adenine dinucleotide phosphate (NAADP) targets two-pore channels (TPCs) to mediate Ca^2+^ efflux from acidic endolysosomal compartments.^88,89^ Structurally, IP₃Rs and RyRs share a conserved architecture, although RyRs have acquired additional cytoplasmic regulatory domains. Their large molecular size allows them to interact with numerous proteins and small molecules, thereby modulating channel gating kinetics.^90^ Ligand activation (via IP₃ or ryanodine) triggers calcium flux that regulates cytosolic Ca^2+^ concentrations. IP₃Rs participate in cytoskeletal organization and cellular adhesion processes,^91^ with osteoclast attachment requiring functional IP₃R-associated cGMP-dependent kinase substrates.^92^ Notably, RyR-like molecules localize to osteoclast plasma membranes, where they function as extracellular Ca^2+^ sensors. The surface-expressed RyR-2 mediates Ca^2+^ influx and serves as a divalent cation sensor.^93^

TPC represents another class of ligand-gated calcium channels distinguished by its unique structural architecture. The TPC family consists of three subtypes: TPC1, TPC2, and TPC3, though only TPC1 and TPC2 are expressed in human and murine cells. These subtypes exhibit distinct subcellular localization patterns, with TPC1 present across various endolysosomal compartments while TPC2 shows predominant localization to late endosomes and lysosomes.^94^ TPCs have emerged as key components of the NAADP-regulated calcium channel system, notwithstanding their additional function as phosphoinositide PI(3,5)P₂-regulated sodium channels.^95^ Under low magnesium conditions, TPC2 activity and osteoclast differentiation are inhibited via the PI(3,5)P₂ signaling pathway.^96^ While the precise activation mechanisms involving NAADP and PI(3,5)P₂ remain debated, initial homology modeling of their three-dimensional structures suggested that TPCs employ an asparagine-gated selectivity filter for cation permeation.^97^

##### Ca^2+^ release-activated Ca^2+^ channel

Ca^2+^ release-activated Ca^2+^ channels (CRAC) on the plasma membrane constitute the principal calcium entry pathway in non-excitable cells, a process termed store-operated calcium entry (SOCE).^98^ This mechanism involves two core components: stromal interaction molecule (STIM), an endoplasmic reticulum (ER)-resident calcium sensor, and Orai, a plasma membrane-localized calcium-selective channel.^98,99^ Following ER calcium store depletion, STIM undergoes activation, oligomerisation, and subsequent coupling with Orai to initiate calcium influx.^100,101^ The Orai family comprises three highly conserved homologs (Orai1-3). Orai1 regulates mineral resorption through its participation in osteoclast differentiation. Genetic ablation of Orai1 impairs both osteoclast and osteoblast differentiation, ultimately compromising skeletal development.^102^ *Orai1*-deficient mice exhibit osteopenia characterized by reduced bone mineral density and diminished trabecular bone volume.^102^ Mechanistically, Orai1-mediated calcium influx facilitates osteoclast differentiation and function through activation of the transcription factor NFATc1.^103^ While Orai2 remains less extensively characterized in osteoclasts, it displays functional similarities to Orai1. Both Orai1- and Orai2-mediated calcium release-activated calcium currents demonstrate regulation by intracellular pH, whereas Orai3 does not.^104^ Furthermore, heteromeric Orai1-Orai2 complexes have been implicated in store-operated calcium entry within chondrocyte cell lines.^105^

##### Transient receptor potential channel

Transient receptor potential (TRP) channels constitute a family of non-selective cation channels permeable to sodium, calcium and other cations.^87^ Notably, certain TRP members exhibit preferential calcium conductivity. The TRP channel superfamily comprises seven subfamilies with distinct evolutionary lineages: TRPC (canonical, 1-7), TRPV (vanilloid, 1-6), TRPM (melastatin, 1-8), TRPA (ankyrin, 1), TRPP (polycystin, 2/3/5), TRPML (mucolipin, 1-3), TRPN (NOMPC in Drosophila, 1), and TRPY (yeast-specific).^106^

TRPC1 and the inhibitor of the MyoD family A (I-mfa) appear to exert antagonistic effects on CRAC channel modulation, consequently regulating osteoclastogenesis.^107^ Furthermore, TRPA1 has been demonstrated to promote osteoclastogenesis and exacerbate osteoporosis through activation of SRXN1-mediated endoplasmic reticulum stress pathways.^108^

Multiple TRPV family members have been characterized in osteoclasts and osteoblasts. TRPV1-TRPV3 proteins show preferential localization in human odontoblast processes, mitochondria and the endoplasmic reticulum.^109^ Notably, TRPV1 and TRPV4 play crucial roles in intracellular Ca^2+^ signaling and extracellular calcium homeostasis in bone cells, with *Trpv1/Trpv4* double knockout mice exhibiting increased bone mass.^110^ TRPV1 deletion impairs fracture healing and inhibits both osteoclast and osteoblast differentiation.^111^ RANKL significantly upregulates TRPV2 expression in RAW264.7 cells, where it regulates osteoclastogenesis through calcium oscillations and NFATc1 activation.^112^ Additional studies demonstrate the involvement of TRPV3 and TRPV6 in osteoclast function and trabecular bone loss.^113^ Similar to TRPV2, TRPV4 contributes to Ca^2+^ signaling pathways and membrane potential depolarization. Expressed in both osteoblasts and osteoclasts, TRPV4 may mediate mechanical stress sensing and bone remodeling regulation.^114^ Parathyroid hormone enhances extracellular calcium entry via TRPV4 channels in osteoblast-like MG-63 cells through cAMP/PKA-dependent mechanisms, influencing cell migration.^115^ TRPV5 and TRPV6 represent the only highly calcium-selective channels in the TRPV family, primarily expressed in renal epithelial cells where they are designated epithelial calcium channels (ECaC1 and ECaC2).^116^ These channels participate in placental and skeletal calcium transport, being present in osteoblasts, osteoclasts and chondrocytes, where they maintain calcium homeostasis during embryonic and fetal development.^117^ In osteoclasts, TRPV5 predominantly localizes to the ruffled border membrane, suggesting a role in bone resorption modulation.^118,119^ TRPV6 exhibits the highest calcium permeability among TRPV channels,^120^ and its knockout induces osteopenia in mice by enhancing osteoclast differentiation and activity.^121^

TRPM4 and TRPM7 are members of the TRPM family, though neither has been previously associated with osteoclast function. TRPM4 channels represent calcium-activated non-selective cation channels exclusively permeable to monovalent ions (K^+^ and Na^+^), exhibiting widespread tissue distribution.^122^ In contrast, TRPM7 demonstrates significant involvement in osteogenesis, being crucial for human osteoblast proliferation and migration.^123,124^ Its expression in mesenchymal cells proves essential for bone formation through the regulation of chondrogenesis.^124^ TRPML1 (encoded by *MCOLN1*), also termed h-mucolipin-1, features six predicted transmembrane domains and functions as a calcium-permeable channel modulated by fluctuating Ca^2+^ concentrations.^125^ This protein relates directly to human mucolipidosis type IV (MLIV), a disorder characterized by lysosomal dysfunction and trafficking defects.^126^ In osteoclasts, the MROS/TRPML1/TFEB axis regulates autophagic processes.^127^ Lysosomal calcium depletion disrupts RANKL-mediated osteoclastogenesis similarly to TRPML1 deletion. *Trpml1*^*−/−*^ bone marrow-derived macrophages display significant lysosomal size abnormalities and impaired resorptive capacity, with *Trpml1*^*−/−*^ mice showing reduced osteoclast numbers and defective bone remodeling.^128^

#### Mitochondrial calcium uniporter

Mitochondria serve dual roles as intracellular calcium stores and energy conversion organelles. Excessive mitochondrial calcium accumulation triggers cell death and contributes to pathological conditions, including ischemia-reperfusion injury.^129^ The mitochondrial calcium uniporter (MCU) mediates calcium uptake into mitochondria and has emerged as a potential therapeutic target.^129,130^ In osteoclasts, MCU participates in differentiation processes, primarily through modulation of the Ca^2+^/NFATc1 signaling pathway.^131^

### Potassium channel

Potassium channels represent the most diverse and abundantly expressed ion channel family in living organisms. These channels regulate bidirectional potassium flux (K^+^) and modulate membrane polarization.^132^ Their involvement in osteoclast differentiation has been well established.^133–135^ Potassium channels are primarily classified into five major categories: (1) calcium-activated potassium channels, (2) inwardly rectifying potassium channels, (3) tandem pore domain potassium channels, (4) voltage-gated potassium channels, and (5) hyperpolarization-activated cyclic nucleotide-gated potassium channels.^136^

#### Calcium-activated potassium channel

Calcium-activated potassium channels occupy a unique position among potassium channels due to their crucial role in maintaining K^+^ homeostasis and regulating cell volume. These channels functionally couple elevated intracellular Ca^2+^ concentrations with membrane potential hyperpolarisation.^137^ The family comprises four distinct subfamilies: (1) the large-conductance calcium-activated channel subfamily M (KCNMA1, KCNMB1-4), (2) the large-conductance calcium-activated channel subfamily U (KCNU1), (3) the intermediate/small-conductance calcium-activated channel subfamily N (KCNN1-4), and (4) subfamily T (KCNT1-2).^138–142^

The intermediate-conductance Ca^2+^-activated K^+^ channel (IKCa, also known as KCa3.1), encoded by the *KCNN4* gene, is known to be expressed and functionally important in osteoclasts as well as in a variety of other non-excitable and neoplastic cells.^142^ Its functional mechanisms involve hormonal secretion, cell motility, proliferation, and regulation of Ca^2+^ influx and/or K^+^ efflux^143^ localized to the plasma membrane, KCNN4 modulates macrophage multinucleation through Ca^2+^ signaling regulation, a process essential for both osteoclast formation in bone and multinucleate giant cell development in chronic inflammatory conditions.^133^

The large-conductance Ca^2+^-activated K^+^ (BK) channels exhibit dual activation mechanisms: they can be triggered by calcium when all voltage sensors are in their resting state, and can also be activated by voltage independently of Ca^2+^ presence.^144^ In osteoclasts, BK channels regulate resorptive activity by controlling cathepsin K secretion. Genetic ablation of BK channels in murine models results in an osteoclast-autonomous osteopenia phenotype, particularly evident in juvenile female specimens. BK channel activation confers protection against osteopenia through modulation of mitochondrial calcium homeostasis and enhancement of mitophagy via the SLC25A5/ANT2-PINK1-PRKN pathway.^145^

#### Inwardly rectifying potassium channel

Inwardly rectifying potassium (IRK) channels are unique ion channels that permit unidirectional K^+^ flow along concentration gradients while preventing reverse flow. These channels consist of four subunits, each with two transmembrane domains and extracellular N-termini and intracellular C-termini. Their opening is regulated by ATP concentration,^146^ with C-terminal globulin structures binding ATP to control gating.^147^ The IRK channel family includes 16 KIR subunits (encoded by *KCNJ1-16*) classified into seven subfamilies (KIR1.x-KIR7.x).^148^ Patch-clamp studies show inwardly rectifying K^+^ currents dominate in rabbit osteoclasts, suggesting functional importance.^149^

#### Tandem pore domain potassium channel

Tandem pore domain potassium (K2P) channels include resting potassium channels with open structures, also referred to as leak channels with basal activation. These channels are abundant in both excitable and non-excitable cells, where they perform diverse functions.^150^ The 15 K2P channels are divided into six subfamilies: TWIK (K2P1, K2P6, K2P7), TREK (K2P2, K2P4, K2P10), TASK (K2P3, K2P9, K2P15), TALK (K2P5, K2P16, K2P17), THIK (K2P12, K2P13), and TRESK (K2P18).^151^ KCNK1 (also known as TWIK-1) serves as a negative regulator of osteoclast differentiation. Increased K^+^ influx resulting from its functional blockade may inhibit osteoclast differentiation by suppressing Ca^2+^ oscillations and the JNK-NFATc1 signaling axis.^134^

#### Voltage-gated potassium channel

Voltage-gated potassium channels contribute significantly to cellular excitability.^152^ These channels typically form homotetrameric structures, with each α-subunit containing six transmembrane domains. They often function in conjunction with β-subunits that modulate K^+^ channel gating. These non-integral β-subunits are oxidoreductases that co-assemble with α-subunit tetramers in the endoplasmic reticulum and remain tightly bound to them. Based on subunit distinctions, voltage-gated potassium channels can be classified into 10 subfamilies (Kv1-Kv10, corresponding to subfamilies A-S).^153^

The Kv1.3 channel (encoded by *KCNA3*) modulates bone resorption through regulation of RANKL-dependent osteoclastogenesis.^135^ KVβ2, an auxiliary subunit of Kv1.3, enhances channel function.^154^ Experimental evidence confirms that pharmacological blockade of Kv1.3 channels promotes extracellular matrix mineralization during osteoblast differentiation.^155^

#### Hyperpolarization-activated cyclic nucleotide-gated potassium channel

Hyperpolarisation-activated cyclic nucleotide-gated (HCN) channels belong to the pore-loop cation channel superfamily. In mammals, the HCN channel family consists of four members (HCN1-HCN4) predominantly expressed in cardiac and neural tissues.^156,157^ These channels are activated by membrane hyperpolarisation, exhibit permeability to both Na^+^ and K^+^ ions, and remain constitutively open near resting membrane potentials. Their activation is frequently potentiated through direct cyclic nucleotide binding, particularly cyclic adenosine monophosphate (cAMP).^158^ HCN channel activation attenuates zinc-dependent hyperpolarisation-induced osteoclast differentiation.^159^ Quantitative PCR analysis of RAW264.7 cells detected HCN1 mRNA, with HCN4 showing the highest expression levels among all subunits. HCN4 knockdown reduced the hyperpolarisation-activated current (*I*_h_) in osteoclasts and enhanced zinc-dependent osteoclastogenesis, though this effect was absent without zinc. Additionally, yellow-green light activation of Arch protein induced membrane hyperpolarisation, subsequently accelerating RANKL-mediated osteoclast differentiation.^159^

### Sodium channel

Voltage-gated sodium channels play crucial roles in physiological processes, including action potential generation and ion homeostasis.^160^

#### Voltage-gated sodium channel

The voltage-gated sodium channel NaV1.6 (encoded by *SCN8A*) has been identified in osteoclasts and is demonstrated to participate in bone resorption regulation.^161,162^ *Scn8a*-deficient mice displayed reduced trabecular and cortical bone mass, accompanied by elevated osteoclast activity.^162^

#### Epithelial sodium channel

Epithelial sodium channel (ENaC) participates in osteoclast differentiation and bone resorption regulation.^163^ This observation suggests its functional involvement in osteoclast activity and potentially identifies a novel ENaC-regulated signaling pathway modulating bone metabolism.

Acid-sensing ion channels (ASICs) constitute a class of cation-permeable protein complexes ubiquitously expressed in cell membranes. As members of the epithelial sodium channel/degenerin superfamily, they play crucial roles in pH sensing, pain perception, acid taste transduction, and other physiological processes.^164,165^ Among ASIC subtypes (ASIC1a, ASIC1b, ASIC2a, and ASIC3) expressed in rat osteoclasts, only ASIC1a demonstrates significant upregulation during acidosis. ASIC1a mediates acid-induced osteoclastogenesis through regulation of the transcription factor NFATc1 activation.^166^ Extracellular acidification-triggered ASIC1a activation promotes osteoclast migration and adhesion via modulation of the integrin/Pyk2/Src signaling pathway, mechanisms directly implicated in osteoporotic bone loss.^167^

### Zinc transporter

Zinc transporters are classified into two principal families: SLC39s/ZIPs and SLC30s/ZnTs, which mediate opposing directional zinc (Zn^2+^) transport across cellular and intracellular membranes.^168^ Zinc serves as an essential trace element for osteoclast function, demonstrating dual regulatory roles in bone remodeling. It promotes bone formation by enhancing mineralization through alkaline phosphatase and metalloenzyme regulation, while simultaneously inhibiting osteoclastogenesis and bone resorption via suppression of the NF-κB signaling pathway during osteoclast differentiation.^169^ The ZIP family comprises 14 members (ZIP1-ZIP14), encoded by *SLC39A1* to *SLC39A14* respectively.^170^ ZIP1 serves as the primary zinc uptake transporter, facilitating zinc accumulation and localizing predominantly to the plasma membrane.^171–174^ Within osteoclasts, ZIP1 exhibits pronounced colocalisation with actin in the sealing zone and significantly inhibits resorptive activity.^175^ Its overexpression impairs NF-κB binding capacity, as demonstrated by electrophoretic mobility shift assays.^175^ Additionally, ZIP1 regulates the NPY/NPY1R signaling pathway through MLT to enhance mesenchymal stem cell osteogenic differentiation and fracture healing.^176^ While ZIP2-ZIP12 remain unreported in osteoclasts or bone tissue, these isoforms maintain crucial roles in systemic zinc homeostasis. Their diverse functions include: (1) intestinal zinc absorption, (2) systemic zinc excretion, (3) zinc secretion from ER/Golgi to cytoplasm, (4) skeletal muscle glucose metabolism regulation, (5) breast cancer cell migration modulation, and (6) metallothionein level control.^177–180^ *Zip13*-deficient mice display dental, connective tissue and skeletal abnormalities resembling Ehlers-Danlos syndrome,^181^ featuring impaired maturation of osteoblasts, chondrocytes, odontoblasts, and fibroblasts, coupled with deficient TGF-β synthesis.^182,183^

The SLC30A family comprises cation diffusion facilitator proteins that mediate zinc efflux and intracellular compartmentalization.^184^ This zinc transporter (ZnT) family includes 10 members, with mutations associated with diabetes, growth retardation, osteopenia,^185^ Alzheimer’s disease,^186^ and Parkinsonism.^187^ ZnT5 plays crucial roles in osteoblast maturation and cardiac conduction system maintenance.^185^ Both ZnT5 and ZnT7 suppression increases cellular apoptosis, while their overexpression activates the PI3K/Akt pathway and inhibits high glucose-induced apoptosis.^188^

### Magnesium transporter

Magnesium (Mg) ranks as the second most abundant cellular cation after potassium, playing vital roles in numerous biological processes, including cell cycle regulation, ATPase activity, and metabolic control.^189^ The major magnesium transporter families comprise NIPA, NIPAL, Claudins, ACDP, SLC41, MMgT, Na^+^/Mg^2+^ exchangers, H^+^/Mg^2+^ exchangers, MRS2, and MAGT1/2.^190–194^ Magnesium is indispensable for proper bone development and mineralization.^195–197^

Magnesium stimulates osteoblast activity and participates in bone formation processes.^195^ Deficiency directly reduces bone density, with animal studies demonstrating that inadequate dietary intake promotes osteoporosis development.^196^ Prolonged Mg^2+^ exposure not only overactivates NF-κB signaling in macrophages, increasing osteoclast-like cell numbers, but also impairs bone maturation by suppressing hydroxyapatite precipitation.^196^ Magnesium deficiency affects bone through modulation of key calcium homeostasis regulators, PTH and 1,25(OH)₂D₃.^198,199^ MAGT1 represents a highly specific magnesium channel with functions extending beyond cellular magnesium homeostasis.^200^ Both MAGT1 and TRPM7 show upregulated expression during osteogenic differentiation, with their silencing accelerating this process partially through autophagy activation.^201^ Mitochondrial RNA splicing 2 (MRS2) functions as a Mg^2+^ transporter involved in mitochondrial magnesium homeostasis.^202^ Substantial evidence identifies TRPM6/7 (mediating Mg^2+^ influx), SLC41A1 (facilitating Mg^2+^ extrusion), and SLC41A3 (regulating mitochondrial Mg^2+^) as established magnesium transporters.^203–205^

### Copper transporter

Copper (Cu) serves as a redox-active trace metal in cellular processes, functioning as an essential component of various cuproenzymes. However, excessive labile Cu^2+^ (non-ceruloplasmin-bound copper in serum) exhibits cytotoxicity through Fenton or Haber–Weiss reaction-mediated generation of damaging hydroxyl radicals.^206^ Physiologically, copper promotes bone regeneration via three key mechanisms: (1) enhanced bone formation, (2) inflammation reduction, and (3) osteoclastogenesis inhibition.^207^

Research demonstrates that Ti6Al4V-Cu exerts multiple inhibitory effects in osteoporotic conditions: (1) suppressing macrophage activation, viability and pro-inflammatory cytokine secretion; (2) reducing osteoclast formation; and (3) down-regulating osteoclast differentiation-related genes and proteins.^208^ Conversely, Ti6Al4V-Cu upregulates bone extracellular matrix formation in osteoporotic osteoblasts.^208^

Copper ion transport relies on two distinct mechanisms: Cu-exporting ATPases (encoded by *ATP7A* and *ATP7B*) and copper transporters (encoded by the *SLC31A1* and *SLC31A2* genes). CTR1 (SLC31A1) is ubiquitously expressed and mediates Cu uptake in most cells,^209^ whereas CTR2 (SLC31A2) exhibits cell-specific expression, for instance, in specialized astrocytes harboring Cu^+^-storage vesicles enriched in the lateral ventricles.^210^ The regulation of ATP7A and ATP7B localization and function governs intracellular Cu^+^ distribution through multilayered mechanisms, with cellular Cu^+^ levels directly influencing both transporters.^211^ While no direct studies have examined copper ion channels in osteoclasts, alterations in Cu concentration mediated by these channels may disrupt osteoclast physiology.

### Iron transporter

Iron, an essential and versatile element for virtually all living organisms, plays critical roles in numerous vital biological processes. Cellular iron acquisition primarily occurs through transferrin receptor (TFRC)-mediated endocytosis of transferrin,^212^ while non-transferrin-bound iron uptake is facilitated by specific transporters including ferroportin (SLC40A1), divalent metal transporter 1 (SLC11A2), natural resistance-associated macrophage protein 1 (SLC11A1), ZIP14, L-type and T-type calcium channels, ZIP8, and TRPC6.^213^

Systemic iron homeostasis is regulated through hepcidin-ferroportin interactions, while cellular iron metabolism is controlled post-transcriptionally via iron-regulatory proteins and iron-responsive elements.^214^ Both iron overload and deficiency correlate with compromised bone integrity, indicating that optimal iron levels are essential for maintaining bone homeostasis.^215^ Substantial evidence demonstrates that dysregulated iron concentrations disrupt the differentiation and activity of both osteoclasts and osteoblasts, ultimately promoting bone loss.^215^ The transcription factor Nrf2 activates genes encoding iron transporters, and clinical-stage Nrf2 activators have been shown to inhibit osteoclast differentiation by modulating the iron-ornithine axis.^216^ The iron exporter SLC40A1, which is predominantly responsible for cellular iron efflux and plays a crucial role in systemic iron homeostasis, is expressed in multiple tissues, including the small intestine, liver, and macrophages.^217^ During osteoclast differentiation, transcript levels of transferrin receptor 1 (TFRC) and divalent metal transporter 1 (SLC11A2) are upregulated, while those of ferroportin (SLC40A1) and natural resistance-associated macrophage protein 1 (SLC11A1) are downregulated.^218^ These findings suggest that precise iron homeostasis is critical for the tight regulation of RANKL-mediated osteoclast development.

Ferroptosis, a significant form of regulated cell death, demonstrates important relationships with skeletal cells, including bone marrow mesenchymal stem cells,^219^ osteoblasts, osteocytes, and osteoclasts.^220,221^ Emerging evidence shows that advanced glycation end products trigger osteoblast ferroptosis, thereby exacerbating osteoporosis.^222^ In contrast, melatonin has been shown to suppress osteoblast ferroptosis and enhance osteogenic potential via activation of the Nrf-2/HO-1 signaling pathway.^223^ Mitochondrial ferritin plays a crucial regulatory role in cellular ferroptosis by sequestering iron ions and reducing mitochondrial ferrous iron toxicity.^224^ Iron ions promote osteoclast differentiation and bone resorption through reactive oxygen species generation.^225,226^ The bisphosphonate zoledronic acid (ZA) inhibits osteoclast proliferation by inducing ferroptosis, as demonstrated in RANKL-induced models showing reduced osteoclast viability and increased ferroptotic activity.^225^ During RANKL-induced differentiation, osteoclast ferroptosis is triggered by iron deprivation responses and ferritin phagocytosis. Mechanistically, RANKL stimulation under normoxic (but not hypoxic) conditions decreases iron levels via an iron starvation response characterized by elevated transferrin receptor 1 and reduced ferritin expression.^226^ These findings suggest that ferroptosis induction in osteoclasts may inhibit bone resorption, potentially offering a therapeutic strategy for bone formation disorders.

### PIEZO-type mechanosensitive ion channel

The mechanically gated PIEZO ion channel family represents the first identified class of mammalian mechanosensitive cation channels, comprising two members, Piezo1 and Piezo2.^1,227^

PIEZO, a mechanosensitive ion channel, plays a crucial role in maintaining bone homeostasis through multiple biological processes. This channel mediates osteoblast–osteoclast crosstalk in response to mechanical stimuli.^228^ Genetic studies demonstrate that mice with conditional *Piezo1* or *Piezo2* deletion in osteoblasts (*Runx2-Cre*) or osteoclasts (*Lyz2-Cre*) develop severe osteoporosis with frequent spontaneous fractures, particularly in *Piezo1*-deficient models.^229^ Mechanistically, mechanical loading activates *Piezo1* in osteoblasts, regulating YAP-dependent expression of type II and IX collagens, which subsequently modulate osteoclast differentiation.^228^ The induction of osteoclast formation from hematopoietic progenitors depends on both the amplitude and duration of shear stress; high-amplitude, long-duration stimulation proves most effective.^230^ Notably, low-amplitude mechanical stimulation activates PIEZO1 and SERCA2 while maintaining low extracellular ATP concentrations, thereby inhibiting osteoclast formation and resorption. In contrast, high-amplitude stimulation typically triggers bone-destructive responses.^230^ PIEZO1-mediated mechanosensing in pre-osteoclasts inhibits RANKL-induced osteoclastogenesis via the PP2A/Akt dephosphorylation pathway. However, this suppressive effect on bone resorption is diminished in periodontitis.^231^ Magnetic-aggregation-induced, bone-targeted nanocarriers activate Piezo1 and promote osteogenic–angiogenic coupling, thereby contributing to osteoporotic bone repair.^232^ Additionally, Piezo1 enhances bone formation by mediating M2 macrophage secretion and activating TGF-β1.^233^ Mechanical activation of *Piezo2*-expressing neurons in nociceptive nerves promotes orthodontic tooth movement and increases osteoclast numbers.^234^

### Ionotropic glutamate receptor

Ionotropic glutamate receptors belong to the ligand-gated ion channel family. Upon glutamate binding, these receptors open to permit the flow of ions (e.g., Na^+^, Ca^2+^) across the membrane, inducing rapid depolarization of the postsynaptic neuron. Glutamate ionotropic receptors include N-methyl-D-aspartate (NMDA), α-amino-3-hydroxy-5-methylisoxazole-4-propionic acid (AMPA), and kainate receptors.^235^ The NMDA and AMPA subtypes are encoded by the *GRIN* and *GRIA* gene families, respectively.^236^ NMDA glutamate receptors are expressed by osteoclast precursors and participate in the regulation of osteoclastogenesis via activation of the NF-κB pathway.^237^ Pharmacological blockade of NMDA receptors using MK-801 alters bone metabolism but does not impair the healing of artificial bone defects.^238^ Additionally, ebselen inhibits trabecular bone matrix degradation and osteoclast formation by targeting the NMDA receptor.^239^ The therapeutic potential of NMDA receptors for bone metabolic disorders warrants further investigation.

### Purinergic receptor

Purinergic receptors, which respond to purines and pyrimidines, are non-selective ligand-gated cation channels. They are classified into two principal families based on their ligands and signaling mechanisms: P1 receptors (activated by adenosine) and P2 receptors (activated by ATP, ADP, or UTP). P2 receptors are further subdivided into P2X (ligand-gated ion channels) and P2Y (G protein-coupled receptors, GPCRs). P2X receptors are activated by extracellular ATP, permitting cation influx (e.g., Na^+^, K^+^, and Ca^2+^).^240^ Seven P2X receptor subtypes (P2X1–P2X7) have been identified, which function as either homotrimeric or heterotrimeric complexes.^241^

Extracellular ATP promotes the differentiation of hematopoietic stem cells into myeloid progenitor and osteoclast precursor cells.^242^ Activation of P2X4 purinoceptors induces cation influx and membrane depolarization. Nucleotides released at sites of trauma or inflammation act through these receptors on osteoclasts to stimulate bone resorption.^243^ P2X5 is essential for ATP-mediated inflammasome activation and subsequent IL-1β production in osteoclasts.^244^ Analysis of alveolar bone demonstrated significantly reduced bone loss in *P2x5*^*−/−*^ mice compared to both *P2x7*^*−/−*^ and wild-type controls. Gene expression profiling of gingival tissue revealed markedly decreased levels of IL1b, IL6, IL17a, and Tnfsf11 expression in *P2x5*^*−/−*^ mice relative to controls.^245^ These findings suggest P2X5-mediated purinergic signaling represents a potential therapeutic target for inflammatory bone loss conditions.

Activation of P2X receptors may ameliorate osteoporosis by regulating the balance between osteoblasts and osteoclasts.^246^ ATP serves as a potent stimulator of rodent osteoclast activation and formation. At low concentrations (0.2-2 μmol/L), ATP enhances osteoclast formation and resorption, while higher concentrations (20-200 μmol/L) may adversely affect osteoblasts.^247^ Notably, extracellular ATP has been shown to decrease bone resorption, likely through P2X7-mediated cytotoxic effects on osteoclasts.^248^ High expression of P2X7 promotes spontaneous osteoclast fusion in vitro, with subsequent studies verifying that ATP accumulation stimulates this fusion process.^249^ Mechanistically, P2X7 receptors positively regulate osteoclast formation and bone resorption by activating the PI3K/Akt/GSK3β signaling pathway.^250^ Anti-P2X7 antibodies or oxidized ATP antagonists significantly inhibit the fusion of osteoclast precursors into multinucleated cells. For instance, certain P2X7 antagonists can prevent human blood monocyte formation of osteoclast-like cells in vitro.^251,252^ ATP acting on P2X7 promotes precursor fusion and differentiation, as well as cell apoptosis through downstream signaling pathways including PKC translocation,^253^ nuclear localization of NF-κB,^254^ and activation of NFATc1.^255^ Prolonged ATP exposure induces extensive internalization of P2X7 receptors, thereby blocking the ability of RAW 264.7 cells to fuse into multinucleated osteoclast-like cells.^256^ Furthermore, P2X7 receptor overexpression increases both the number of multinucleated osteoclasts and the expression of osteoclastogenesis-related proteins. Conversely, P2X7 receptor knockdown suppresses osteoclast differentiation by inhibiting autophagy and Ca^2+^/calcineurin signaling.^257^ In summary, P2X7 activation in osteoclasts is essential for cell fusion and plays a critical role in determining cell survival time and function. These findings establish P2X7 as a potential therapeutic target for osteoporosis.

## Part III anion transporter

### Chloride channel

**Fig. 4:**
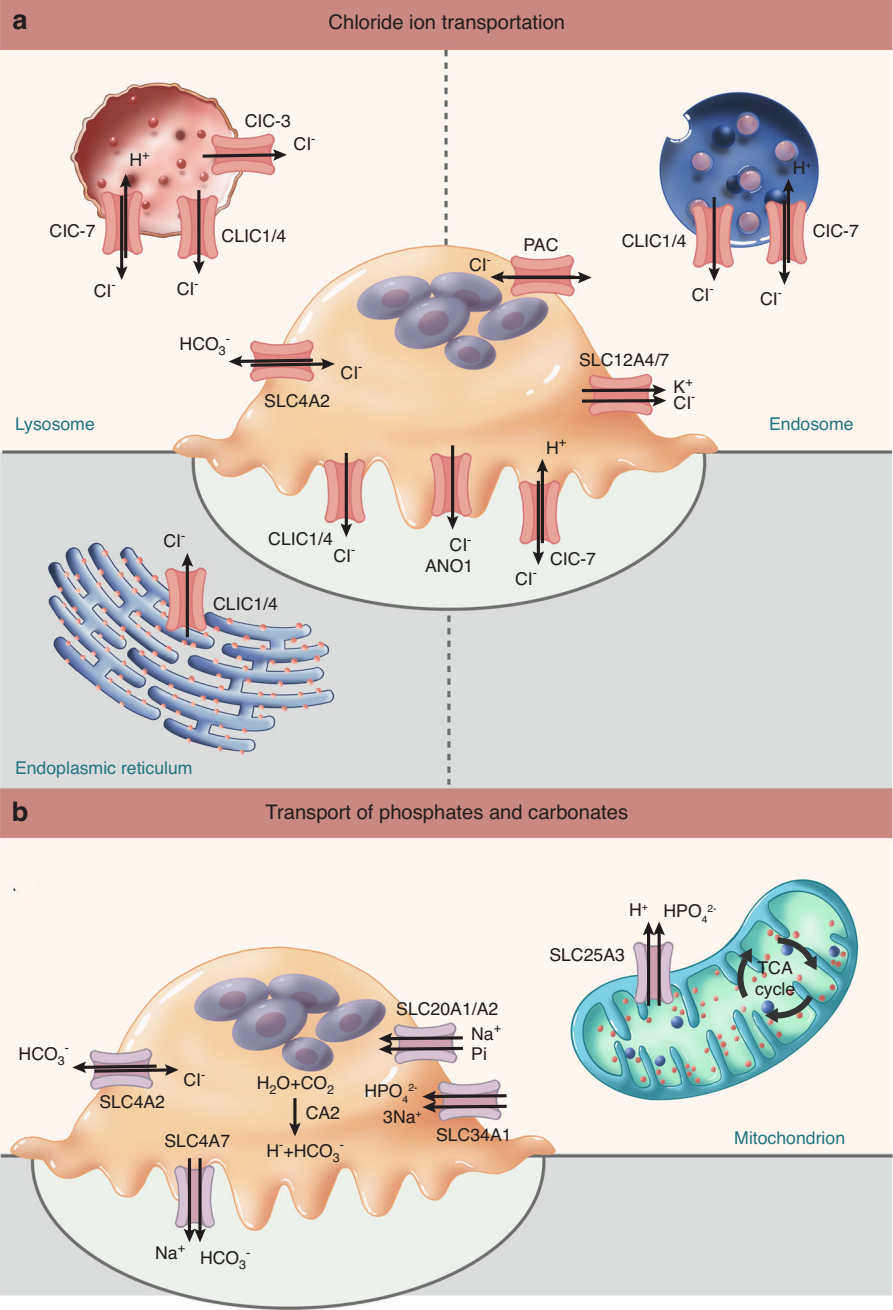
Chloride, phosphate, and carbonate transporters in osteoclasts. **a** Chloride transporters. Voltage-gated chloride channels (ClCs): ClC-7: Localizing to the ruffled border, endosomes, and lysosomes, the ClC-7/OSTM1 complex acts as a Cl^−^/H^+^ antiporter to regulate chloride concentration. ClC-3: Mediates acidification of intracellular organelles, facilitating bone resorption. Chloride intracellular channel 1 (CLIC1): Functions in intracellular vesicles, resorption lacunae, and organelles by modulating chloride dynamics, though its specific osteoclast roles remain under investigation. ANO1: As the key calcium-activated chloride channel (CaCC), it mediates chloride transport at the plasma membrane, thereby regulating bone resorption. Proton-activated chloride channel (PAC): Functions at the plasma membrane by sensing extracellular acidity to regulate pH, promote osteoclast fusion, and mediate resorption-related pain. Chloride/HCO_3_^−^ Exchanger (SLC4A2): Mediates electroneutral Cl^−^/HCO_3_^−^ exchange at the plasma membrane, maintaining electroneutrality and cytoplasmic pH during bone resorption. Potassium/Chloride Symporter (SLC12A4/7): Facilitates coupled K^+^/Cl^−^ transport at the plasma membrane, contributing to chloride homeostasis and osteoclast function. **b** Phosphate and carbonate transporters. Phosphate transporter: Mitochondrial Phosphate Transporter (SLC25A3): Localized in the inner mitochondrial membrane, it facilitates electroneutral phosphate uptake (coupled with H^+^/OH^−^ exchange), supporting oxidative phosphorylation, mitochondrial Ca²^+^ buffering, and energy supply for osteoclast activity. Sodium/Phosphate cotransporters: SLC20A1/A2: Mediates Na^+^-dependent inorganic phosphate (Pi) transport at the plasma membrane to maintain phosphate homeostasis. SLC34A1: Facilitates Na^+^-coupled phosphate uptake, supporting the metabolic and functional demands of osteoclasts. Cl^−^/HCO_3_^−^ Exchanger (SLC4A2): Mediates electroneutral HCO_3_^−^/Cl^−^ exchange at the plasma membrane to maintain electroneutrality, prevent cytoplasmic alkalinization, and supply Cl^−^ to ruffled border chloride channels, thereby supporting lacunar acidification and bone resorption. Electroneutral Na^+^/HCO_3_^−^ Cotransporter (SLC4A7): Localized to the ruffled border, where it mediates Na^+^-driven HCO_3_^−^ transport to regulate intracellular pH and promote osteoclast differentiation and survival. Carbonic Anhydrase 2 (CA2): Catalyzes the conversion of CO₂ and H₂O into H^+^ and HCO_3_^−^, supplying protons for V-ATPase and bicarbonate for transporters such as SLC4A2 and SLC4A7, thus integrating acid–base balance with bone resorption

Chloride channels are ubiquitously expressed in cellular and organelle membranes of organisms, playing crucial physiological and pathological roles in pH regulation, volume homeostasis, resting membrane potential maintenance, and modulation of membrane excitability.^258–260^ These channels contribute significantly to lysosomal acidification and osteoclastic bone resorption, demonstrating functional importance comparable to V-ATPase in osteoclast activity^5,6^ (Fig. 4a). The chloride channel family comprises several distinct classes: voltage-gated chloride channels (ClC), chloride intracellular channels (CLIC), calcium-activated chloride channels (CaCC), proton-activated chloride channels (PAC), volume-regulated anion channels, and the cAMP-dependent cystic fibrosis transmembrane conductance regulator (CFTR).^261^ This review will focus primarily on four subtypes: ClC, CLIC, CaCC, and PAC. Additionally, certain cotransporters including potassium/chloride symporters facilitate chloride ion movement in osteoclasts, which will be discussed in section “Potassium/chloride cotransporter (KCC)”.

#### Voltage-gated chloride channel (ClC)

Chloride channels of the ClC family are encoded by *CLCN* genes. Some members, such as the plasma membrane channel ClC-1, exhibit voltage-dependent gating, while others are intracellular H^+^/Cl^−^ exchangers with a complex dependence on membrane potential and ionic environment. These channels localize to either plasma membranes or intracellular organelles within osteoclasts.^262^

The ClC-7 chloride channel is encoded by the *CLCN7* gene in humans. Mutations in *CLCN7* cause two distinct forms of osteopetrosis: autosomal dominant osteopetrosis type II (OMIM 166600; ADOII) and autosomal recessive osteopetrosis (OMIM 611490, ARO).^263,264^ Notably, *CLCN7* mutations have been identified in a unique clinical phenotype combining osteopetrosis with renal tubular acidosis, renal stones, epilepsy, blindness and Alzheimer’s disease.^263,265,266^ Both mouse models and human studies demonstrate that loss of functional ClC-7 channels leads to osteopetrosis.^264^ At the cellular level, ClC-7 plays critical roles in regulating chloride ion concentration within endosomal and lysosomal compartments. This regulation is essential for proper vesicle trafficking, organelle acidification, and formation of the osteoclast ruffled border, which is required for acidification of the resorption lacuna and subsequent bone resorption.^267^ Functioning as a Cl^−^/H^+^ antiporter, ClC-7 typically forms a complex with OSTM1 and significantly influences both osteoclast differentiation and bone resorption activity. Mutations in either *CLCN7* or *OSTM1* disrupt normal endosomal/lysosomal function, impair ruffled border formation, and ultimately lead to defective bone resorption and pathological bone accumulation.^268^ The subcellular localization of the ClC-7/Ostm1 complex has important clinical implications.^268^ When this complex is absent from lysosomal membranes, patients develop neurodegeneration that does not respond to hematopoietic stem cell transplantation (HSCT).^269^ However, ARO patients without neurodegeneration may benefit from potentially curative HSCT.^269^ For mutations that specifically impair lysosomal membrane localization of the ClC-7/Ostm1 complex, pharmacological chaperone therapy represents a promising therapeutic strategy currently under investigation for lysosomal storage disorders.^270^ Beyond its role in bone resorption, ClC-7 contributes to several other physiological processes. The channel facilitates chloride accumulation within phagosomes, thereby enhancing hydrolase activity and promoting phagosome resolution.^271^ It also plays a crucial role in microglial phagocytic clearance, as demonstrated by zebrafish *clcn7* mutants that cannot properly process extracellular debris, including apoptotic cells and β-amyloid protein.^272^ Furthermore, *CLCN7* downregulation appears important for the development of mechanical and thermal hyperalgesia, with siRNA-mediated silencing of *Clcn7* in dorsal root ganglia exacerbating pain responses in naive mice.^273^ Dental development is also affected by ClC-7 dysfunction, with *Clcn7*^*−/−*^ mice exhibiting dental dysplasia due to impaired osteoclast activity.^274^ The channel regulates craniofacial bone and tooth development through the ClC-7/CTSK/TGF-β/BMP/SMAD signaling pathway.^275^ From a therapeutic perspective, antibodies targeting ClC-7 can inhibit acidification-induced chloride currents and reduce bone resorption activity in mouse osteoclasts, suggesting potential applications for osteoporosis treatment.^276^

ClC-3 channels are localized to intracellular organelles within murine osteoclasts and facilitate osteoclastic bone resorption in vitro by mediating organelle acidification.^277^ Both organelle acidity and bone resorption activity were significantly impaired in *Clcn3*^*−/−*^ osteoclasts. The channel’s role in osteodifferentiation appears mediated through the Runx2 pathway. Overexpressed ClC-3 protein co-localizes with TGF-β1 in intracellular organelles, and channel inhibition may promote endosomal acidification.^278^ Furthermore, ClC-3 may function as a mechanosensitive channel that regulates osteoblast differentiation via integrated signaling through Runx2, TGF-β1, and Wnt pathways.^279^

#### Chloride intracellular channel (CLIC)

The CLIC family comprises six evolutionarily conserved proteins in humans (CLIC1–CLIC6).^280^ All CLIC members exist in two distinct forms: a soluble cytoplasmic form and an integral membrane form. The membrane-associated form primarily localizes to the plasma membrane and intracellular organelle membranes, where it functions as an ion channel.^281^

During osteogenic differentiation, CLIC1 expression is downregulated specifically during early passages, potentially through eukaryotic translation elongation factor-mediated regulation.^282^ CLIC1 modulates macrophage function by regulating phagosomal acidification.^283^ Although highly expressed in intracellular vesicles and resorption lacunae of osteoclasts,^284^ the precise functional role of CLIC1 in these cells remains unclear. Notably, acidification signals appear to colocalize with ClC-7 rather than CLIC1 in osteoclasts. CLIC4 participates in diverse biological processes depending on its subcellular distribution, including cellular differentiation, apoptosis,^285^ membrane remodeling,^286^ actin cytoskeleton reorganization, and cell adhesion.^287^ While low mRNA levels of CLIC4 have been detected in human osteoclasts, its specific biological functions in these cells remain to be elucidated.^284^ Both CLIC1 and CLIC4 contribute to NLRP3 inflammasome regulation in LPS/ATP-stimulated bone marrow-derived macrophages.^288^ CLIC4 specifically reduces bone morphogenetic protein receptor II expression and signaling via an Arf6-dependent mechanism that both decreases gyrating clathrin recruitment and enhances receptor targeting to lysosomes.^289^

#### Calcium-activated chloride channel (CaCC)

CaCCs have been classified into three subfamilies based on their encoding genes, the chloride channel accessory family (CLCA1-4), the bestrophins family (BEST1-3), and the anoctamins family (ANO1-10).^290^ These channels are calcium-activated and mediate chloride ion transport across cellular membranes, with the direction of chloride movement being determined by both the channel’s properties and the intracellular chloride concentration.^291^ CaCCs exhibit widespread tissue distribution and have been detected in nearly all tissue types including retinal tissue, osteoblasts, neurons, secretory epithelia, skeletal muscle, immune cells, and platelets. The physiological functions of CaCCs vary according to their cellular and tissue localization, and include diverse processes such as bone ossification, neurotransmitter release, epithelial secretion, regulation of membrane excitability in cardiac muscle and neurons, as well as phagocytic activity.^291–295^

ANO1 represents a key member of the CaCC family. Studies using osteoclast-specific *Ano1* knockout mice revealed increased bone mass coupled with reduced bone resorption activity.^283^ ANO1 plays an essential role in bone resorption regulation by functionally connecting chloride channel activation with RANKL-RANK signaling pathways.^283^ Mutations in ANO5 cause gnathodiaphyseal dysplasia (GDD), characterized by thickened long bone and mandibular cortices, narrowed medullary cavities, and enhanced bone fragility.^296^ Mechanistically, ANO5 promotes osteoclast differentiation through AKT activation, primarily influencing osteoclast rather than osteoblast differentiation during normal bone remodeling.^297^ *Ano5* deficiency disrupts bone formation by inducing osteoclast apoptosis in GDD and activates autophagy in murine cranial osteoblasts via AMP-activated protein kinase (AMPK) and ATG9A pathways.^297,298^ ANO6 possesses ten transmembrane domains containing five acidic residues involved in calcium binding.^299^ This channel mediates diverse biological functions including cell migration, volume regulation, platelet activation/apoptosis,^295^ and glioma cell proliferation and invasion through ERK signaling modulation.^300–302^ Genetic deletion of *Ano6* causes skeletal deformities and mineralization defects in mice. *Ano6*^*−/−*^ osteoblasts lack Ca^2+^-activated anion currents, and the Na^+^/Ca^2+^ exchanger NCX1 requires ANO6 for efficient Ca^2+^ extrusion into the mineralizing bone matrix.^292^ While ANO6 function in osteoclasts remains uncharacterized, its involvement in macrophage apoptosis via intracellular Ca^2+^ (iCa^2+^) elevation may provide insights into potential roles in osteoclast biology and bone resorption regulation.

#### Proton-activated chloride channel (PAC)

PAC is an evolutionarily conserved membrane protein that functions as a chloride channel activated by extracellular protons.^303^ It precisely regulates extracellular and endosomal (early and late) pH by facilitating chloride ion transport across membranes.^304,305^ This pH-regulatory function is particularly critical in cellular processes that depend on precise pH control, such as osteoclast differentiation and activity. During osteoclast differentiation, PAC expression is induced via the RANKL/NFATc1 signaling pathway, a central regulatory cascade in osteoclastogenesis.^306^ Extracellular acidosis elicited robust PAC currents in osteoclasts. The acidic microenvironment of porous endplates, combined with enhanced PAC activation, promotes osteoclast fusion, contributing to low back pain underscoring PAC’s pivotal role in osteoclast-mediated bone resorption and pain signaling.^306,307^ PAC channel activation may also upregulate ST3GAL1 sialyltransferase expression and TLR2 sialylation during osteoclast fusion initiation.^306^ Consequently, elevated Pacc1 expression and PAC activity represent potential therapeutic targets for treating LBP and osteoclast-associated pain.^306^

### Phosphate transporter

Mitochondrial phosphate transport is mediated by phosphate carrier proteins and cotransporters. The phosphate carrier proteins are encoded by *SLC25A* family genes, which constitute a group of nuclear-encoded transporters predominantly localized to the inner mitochondrial membrane.^308^ These proteins characteristically contain six transmembrane segments.^308^ The phosphate cotransporters will be discussed in Section “Sodium/Phosphate cotransporter”.

SLC25A3 is a member of the SLC25A family that functions as an electroneutral phosphate transporter in mitochondria.^309^ It facilitates mitochondrial phosphate uptake through exchange with OH^−^ or cotransport with H^+^^309^ (Fig. 4b). Beyond its primary role in phosphate transport, SLC25A3 may also function as a copper transporter involved in cytochrome c oxidase complex biogenesis, a process essential for cellular energy production.^310^ In osteoclasts, SLC25A3 fulfills multiple critical functions, including meeting energy demands through oxidative phosphorylation, buffering mitochondrial matrix Ca^2+^ loads,^311^ regulating cellular apoptosis,^312^ and mediating mitochondrial permeability transition pore formation.^313^ Notably, METTL14-mediated targeting of SLC25A3 has been shown to restore mitochondrial reactive oxygen species levels and membrane potential in osteoblasts, which promotes osteoblast differentiation and contributes to the amelioration of osteoporosis.^314^

SLC25A4 has been identified as a novel modulator of mitochondrial dysfunction and apoptosis in specific cardiomyocyte subpopulations.^315^ SLC25A5 contributes to various cellular processes, including metabolism, lipid metabolism, cell growth and apoptosis.^316–318^ The dicarboxylate carrier SLC25A10 serves as a crucial regulator of human energy metabolism and redox homeostasis, facilitating the transport of dicarboxylate substrates that support multiple metabolic pathways, such as sulfur metabolism and gluconeogenesis.^319^ Additionally, SLC25A10 participates in fatty acid synthesis, glucose-stimulated insulin secretion and other physiological processes.^320,321^ SLC25A10 shows significantly higher expression levels in human osteosarcoma tissues compared to normal bone tissue.^322^ While no direct evidence currently links these three SLC25 family members to osteoclast function, their potential to indirectly influence osteoclast differentiation and activity through the modulation of cellular energy states and metabolic environments warrants further investigation.

### Bicarbonate transporter

Bicarbonate transporters are primarily encoded by the *SLC4* gene family, which comprises ten members (*SLC4A1-5* and *SLC4A7-11*).^323^ These transporters can be functionally categorized into several groups: Cl^−^/HCO₃^−^ exchangers (AE1-3; *SLC4A1-A3*), electrogenic Na^+^/HCO₃^−^ cotransporters (NBCe1/SLC4A4 and NBCe2/SLC4A5), electroneutral Na^+^/HCO₃^−^ cotransporters (NBCn1/SLC4A7, NBCn2/SLC4A10 and NCBE/SLC4A8), and Na^+^-driven Cl^−^/HCO₃^−^ exchangers.^323,324^ All bicarbonate transporter members share a conserved structural architecture with 10-14 transmembrane domains.^324^ Within this family, SLC4A2 and SLC4A7 have been identified as particularly crucial for osteoclast function and bone resorption processes.

SLC4A2 belongs to the Na^+^-independent HCO₃^−^/Cl^−^ anion exchanger subfamily and is widely expressed in multiple tissues, including the gastrointestinal tract, kidney and bone.^325,326^ During osteoclast differentiation and bone resorption, SLC4A2 expression is significantly upregulated.^327^ This upregulation is crucial for maintaining osteoclast electroneutrality and preventing cytoplasmic alkalinisation.^328^ The transporter facilitates bicarbonate secretion while simultaneously increasing cytoplasmic Cl^−^ concentration through HCO₃^−^/Cl^−^ exchange, thereby supplying chloride channels in the ruffled membrane (Fig. 4b). SLC4A2 deficiency in osteoclasts severely compromises lacunar acidification and bone resorption capacity. Beyond pH regulation, SLC4A2-mediated Cl^−^/HCO_3_^−^ exchange plays a vital role in calpain-dependent actin cytoskeleton regulation in osteoclasts.^329^ Studies using *Slc4a2*^−^/^−^mice demonstrate that SLC4A2 promotes optimal osteoclast differentiation while suppressing apoptosis. Both human and murine models show that SLC4A2 deficiency leads to osteopetrotic phenotypes.^330,331^

NBCn1 (encoded by *SLC4A7*), a member of the electroneutral Na^+^/HCO₃^−^ cotransporter family, is functionally critical for osteoclast differentiation and bone resorption (Fig. 4b). Immunofluorescence studies confirm its specific localization to the osteoclast ruffled border, with expression upregulation observed during both differentiation and resorption phases.^332^ At the ruffled border, NBCn1 mediates bicarbonate uptake from resorption lacunae, while at the basolateral membrane it facilitates bicarbonate efflux.^332^ This activity is regulated by colony-stimulating factor-1 (CSF-1), which enhances intracellular pH and promotes osteoclast survival.^333^ Recently, mTORC1 was shown to stimulate the intracellular transport of HCO₃^−^ to promote nucleotide synthesis through selective translational regulation of the sodium bicarbonate cotransporter SLC4A7 (ref. ^334^).

Both SLC4A2 and SLC4A7 are critical for osteoclast function, and their roles in maintaining intracellular pH and facilitating bone resorption underscore their potential as therapeutic targets for bone-related disorders.

### Nitrate transporter

Nitrate exhibits a broad spectrum of biological functions across eukaryotic, bacterial and plant cells.^335–337^ In humans, nitrate transport is principally linked to cellular ion homeostasis and NO metabolism.^338^ The Nrt1 and Nrt2 protein families constitute the high- and low-affinity nitrate transporter systems, respectively.^339,340^ However, nitrate transporters in human and animal cells remain less characterized compared to other ion channels or transport proteins.

Nitrate modulates osteoclast differentiation through NO signaling. Direct NO donors regulate both osteoblast and osteoclast activity, increasing bone mass in ovariectomised mice.^338^ Nitrosyl-cobinamide reduces osteoclast numbers in intact mice and prevents the ovariectomy-induced increase in osteoclasts. This occurs via two mechanisms: (1) reducing of the RANKL/osteoprotegerin gene expression ratio (a key regulator of osteoclast differentiation), and (2) directly inhibiting osteoclast differentiation, as demonstrated in vitro under excess RANKL conditions.^338^ In osteoblasts, NO exerts positive effects through the cGMP/PKG pathway,^338^ while in osteoclasts, cytoplasmic NO inhibits differentiation and bone resorption by suppressing acid secretion and cellular attachment.^341^ Consequently, inhibiting nitrate transporters may reduce NO production, thereby suppressing osteoclast differentiation and resorptive activity. Reduced NO bioavailability in bone tissue contributes to diabetes-induced osteoporosis,^342^ underscoring NO signaling’s importance in bone homeostasis. Despite these findings on NO’s role in osteoclasts, nitrate transporter identity and expression patterns remain poorly characterized.

## Part IV cotransporters, symporters, or antiporters

### Sodium/hydrogen exchanger

Sodium/hydrogen exchangers (NHEs) play a critical role in maintaining bone homeostasis and regulating osteoclast function. As members of the P-type ATPase superfamily, NHEs mediate the electroneutral exchange of one intracellular proton (H^+^) for one extracellular sodium ion (Na^+^).^343^ The genes encoding NHEs are classified within the SLC9 family, which is divided into three subgroups (A, B, and C).^344^ The SLC9A subgroup includes both plasmalemmal isoforms (NHE1-NHE5, encoded by *SLC9A1*-*SLC9A5*) and predominantly intracellular isoforms (NHE6-NHE9, encoded by *SLC9A6*-*SLC9A9*). The SLC9B subgroup contains two isoforms: NHA1 (*SLC9B1*) and NHA2 (*SLC9B2*). The SLC9C subgroup consists of a sperm-specific plasmalemmal NHE (*SLC9C1*) and a putative NHE (*SLC9C2*), though no functional data are currently available for the latter.^344^ Additionally, two regulatory cofactors (NHE-RF1 and NHE-RF2) are encoded by *SLC9A3R1* and *SLC9A3R2*, respectively.^344^

The human gene *SLC9A1* encodes the NHE1 isoform, which comprises a 500-amino acid membrane domain responsible for ion transport and a 315-amino acid cytoplasmic C-terminal regulatory domain.^343^ In most mammalian species, NHE1 is ubiquitously expressed across cell types, with notable exceptions being the macula densa and renal α- and β-intercalated cells.^345,346^ As the primary mediator of extracellular Na^+^/intracellular H^+^ exchange, NHE1 serves as the principal regulator of pH homeostasis in both normal and neoplastic cells.^347^ Extensive research has demonstrated NHE1’s critical involvement in cellular processes, including growth, proliferation, differentiation and apoptosis.^348^ Regarding bone resorption, NHE1 collaborates with V-ATPase and other key enzymes to mediate the acidification required for osteoclast activity. NHE6 shows predominant expression in osteoblast basolateral membranes, particularly in highly mineralized regions where it eliminates excess H^+^ generated during mineralization.^349^ Genetic studies reveal that *Nhe6* deficiency results in reduced bone volume in adult mice.^350^ NHE10 exhibits marked upregulation in osteoclasts following RANKL ligand stimulation and is essential for osteoclast differentiation and survival.^351^ Therapeutic studies suggest that anti-NHE10 monoclonal antibodies may serve as effective bone resorption inhibitors for targeted treatment of bone disorders.^352^ While NHE10’s osteoclastic role is well-established, the precise physiological functions of NHE1 and NHE6 in osteoclasts require further investigation.

The Na^+^/H^+^ exchanger SLC9B2 (NHA2) exhibits a distinctive structural organization among Na^+^/H^+^ exchangers, featuring 14 transmembrane segments with an additional N-terminal helix.^353^ This configuration creates a unique homodimer interface that potentially functions as a lipid-mediated regulatory switch.^353^ NHA2 shows significant enrichment in osteoclast plasma membranes, with particularly strong localization at the basolateral membrane of polarized osteoclasts.^354^ During RANKL-induced osteoclast differentiation, NHA2 demonstrates marked upregulation both in vitro and in vivo, where it may serve dual roles as both a plasma membrane transporter and mitochondrial cation-proton antiporter.^354^ This mitochondrial localization enables NHA2 to mediate Na^+^-dependent pH modulation and facilitates Na^+^-acetate-induced mitochondrial passive swelling.^355^ Functional studies confirm that *Nha2* RNA silencing effectively inhibits osteoclast differentiation and bone resorption activity.^356^

Sodium/hydrogen exchangers, particularly NHE1, NHE6, NHE10 and NHA2, perform diverse yet critical functions in bone biology. These membrane transporters regulate three fundamental processes: (1) maintenance of intracellular pH homeostasis, (2) modulation of ionic balance, and (3) control of osteoclast differentiation. Their coordinated activity is essential for normal bone resorption and remodeling. While the physiological roles of certain NHE isoforms (notably NHE1 and NHE10) in bone have been relatively well characterized, the specific functions of other family members (including NHE6 and NHE9) remain to be fully elucidated.

### Sodium/calcium exchanger

The sodium/calcium exchanger (NCX) family comprises three members (NCX1, NCX2 and NCX3), which collectively regulate cellular Ca^2+^ efflux and maintain intracellular Ca^2+^ homeostasis.^357^ Of these isoforms, NCX1 has been most extensively characterized for its functional role in osteoclast biology.

RANKL-induced differentiation of murine osteoclast precursors into mature osteoclasts significantly downregulated NCX1 expression, while NCX2 and NCX3 levels remained largely unchanged. In vitro studies demonstrate that although NCX1 is dispensable for osteoclast differentiation, its deficiency enhances resorptive activity, suggesting a critical role in modulating bone resorption and calcium homeostasis.^358^ Through Na^+^/Ca^2+^ exchange, osteoclasts exhibit rapid, sustained, yet reversible cytosolic Ca^2+^ elevation upon Na^+^ withdrawal. Furthermore, NCX1 couples H^+^ extrusion with Ca^2+^ flux via Na^+^ exchange.^359^ NCX1 disruption alters calcium homeostasis and promotes RANKL-induced osteoclast differentiation by activating the JNK/c-Fos/NFATc1 signaling pathway.^360^

### Sodium/phosphate cotransporter

Sodium/phosphate cotransporters are key molecules for osteoclasts to maintain bone resorption function and energy metabolism. They mainly mediate sodium ion-dependent inorganic phosphate (Pi) transport to provide osteoclasts with the energy substrates required for bone resorption, and are involved in cell polarization and functional regulation.^361,362^ Phosphate transporters are classified into three types: type I (SLC17 family), type II (SLC34 family), and type III (SLC20 family).^363,364^

Previous studies have confirmed the expression of type II and III phosphate transporters in the basolateral domain.^365,366^ NaPi-IIa (SLC34A1/NPT2) has been implicated in osteoclast function, though its role remains controversial. Early studies detected its expression in chicken, rabbit, and mouse osteoclasts. In these cells, NaPi-IIa exhibits a dynamic subcellular localization: it resides in intracellular vesicles in unpolarized osteoclasts, but redistributes to the basolateral membrane upon polarization on bone, colocalizing with NHE-1 and opposing the V-ATPase at the ruffled border.^361,362^ Functionally, NaPi-IIa was proposed to mediate uptake of bone-derived phosphate to support ATP production, with its inhibition reducing ATP levels and impairing bone resorption.^361^ This activity is regulated by PI3-K signaling, as wortmannin (PI3-K inhibitor) blocks phosphate uptake and NaPi-IIa trafficking. The inhibitor phosphonoformic acid also suppresses both transporter activity and bone resorption in a dose-dependent manner.^361^ However, a subsequent study challenged this paradigm, reporting extremely low NaPi-IIa mRNA in osteoclasts, with only transient detection in precursors and none in RAW 264.7 cell-derived osteoclasts.^367^ Consistently, NaPi-IIa knockout mice showed no defects in osteoclast differentiation or bone resorption compared to wild-type controls. These conflicting findings highlight the need to re-evaluate the essentiality of NaPi-IIa in osteoclast biology.^367^

The SLC20 family transporters, Pit-1 (SLC20A1) and Pit-2 (SLC20A2), are ubiquitously expressed sodium-dependent phosphate (Pi) transporters that serve as the principal Pi carriers in osteoclasts. Their expression remains stably high throughout osteoclast differentiation and is unaffected by RANKL signaling. Both Pit-1 and Pit-2 are readily detectable at the mRNA and protein levels in osteoclast precursors and mature osteoclasts, substantially exceeding the expression of NaPi-IIa.^367^ Functionally, SLC20 transporters preferentially import monovalent Pi and are pH-insensitive, allowing sustained Pi uptake even in the acidic extracellular milieu of polarized osteoclasts. They mediate the majority of sodium-dependent Pi transport in osteoclasts and can fully compensate for the loss of NaPi-IIa, maintaining ATP production and bone resorption activity.^367^ Notably, Pit-1 also exhibits transport-independent roles. Its reduced expression impairs precursor proliferation and mitosis, potentially through modulation of the p38 MAPK pathway, thereby influencing differentiation. In vivo, complete Pit-1 knockout is embryonically lethal, whereas mice with low Pit-1 expression (~15% residual) exhibit compensatory upregulation of Pit-2 in bone and normal mineralization, underscoring the functional redundancy between Pit-1 and Pit-2 in osteoclasts.^367^

### Potassium/chloride cotransporter (KCC)

Potassium/chloride cotransporter (KCC) is an electroneutral transporter responsible for the transmembrane transport of potassium and chloride ions.^368^ The KCC family comprises two distinct subgroups: (1) Na^+^-K^+^-2Cl^−^ cotransporters (NKCC), including NKCC1 (*SLC12A1*) and NKCC2 (*SLC12A2*), which mediate cation influx and increase intracellular Cl^−^ concentration; and (2) K^+^-Cl^−^ cotransporters (KCC1-4; *SLC12A4-7*), which promote Cl^−^ efflux and consequently decrease intracellular Cl^−^ levels.^369,370^

KCC is widely distributed across various tissues and plays crucial roles in multiple physiological processes, including cell volume regulation, chloride concentration homeostasis, epithelial secretion,^371^ renal reabsorption, and both bone ossification and resorption.^372^ In osteoclasts, KCC1 functions primarily in regulating intracellular chloride concentration, which directly facilitates H^+^ secretion through the ruffled border onto the bone surface. The KCC inhibitor DIOA was shown to significantly reduce bone resorption activity while having minimal effect on osteoclast numbers.^372^ These findings suggest that KCC1 modulates bone resorption through its regulation of H^+^ extrusion into the resorption lacuna. In contrast to KCC1, KCC2 and KCC3 have been predominantly studied in neurological disorders and tumor biology, with their potential roles in osteoclast function remaining largely unexplored.

## Part V Organic substance transporter

### Glucose transporter

Glucose transporters play a crucial role in maintaining stable glucose concentrations in circulation and facilitating cellular glucose uptake for both catabolic and anabolic processes.^373^ These transporters are essential for regulating proliferation, differentiation and tissue-specific cellular functions. Glucose transporters comprise two distinct families: the facilitative glucose transporter family (GLUT) and the Na^+^/glucose cotransporter family (SGLT). The GLUT family includes 14 members encoded by *SLC2A1-14*, whereas the SGLT family consists of three subtypes: SGLT1 (*SLC5A1*), SGLT2 (*SLC5A2*) and SGLT3 (*SLC5A4*).^374^

Glucose serves as the primary energy source for osteoclasts during bone resorption.^375^ The expression of GLUT1, a key glucose transporter, is markedly upregulated during osteoclast differentiation and bone resorption.^376^ Inhibition of HIF-1α, a crucial regulator of glucose metabolism, significantly reduces GLUT1 expression and impairs bone resorption activity, while having minimal effect on osteoclast differentiation.^375^ GLUT1-mediated glucose metabolism, involving both lactate production and oxidative phosphorylation, is essential for normal osteoclastogenesis.^377^ As energy supply is fundamental for all cellular processes, it is reasonable to propose that GLUT transporters contribute to bone resorption by regulating glucose uptake and subsequent energy provision in osteoclasts.

### (Pi)-linked G6P anti-porter

The phosphate (Pi)-linked glucose-6-phosphate (G6P) antiporters, encoded by the *SLC37* family, belong to the organophosphate-Pi antiporter (OPA) family. This family comprises four members (SLC37A1-4) that demonstrate ubiquitous tissue distribution.^378^

SLC37A2 is a macrophage-specific protein predominantly expressed in neutrophils and macrophages of the endoplasmic reticulum (ER).^379,380^ In osteoclasts, *SLC37A2* demonstrates particularly strong expression compared to other family members. This protein localizes to the limiting membrane of secretory lysosomes, dynamic tubular organelles essential for osteoclast-mediated bone resorption. Genetic ablation studies reveal that *Slc37a2*-deficient mice develop high bone mass due to uncoupled bone metabolism and impaired monosaccharide export from secretory lysosomes, a process critical for maintaining the bone-lining plasma membrane in osteoclasts.^381^ These findings underscore the pivotal role of SLC37A2 in regulating both secretory lysosome dynamics and monosaccharide transport during bone resorption. Consequently, SLC37A2 represents both a physiological component of osteoclast-specific secretory organelles and a promising therapeutic target for metabolic bone disorders.

### Nucleoside transporter

Nucleosides are essential cellular components that play critical roles in gene transcription and signaling pathways. Nucleoside transporters are classified into two families based on their transport mechanisms and structural characteristics: equilibrative nucleoside transporters (ENTs) and concentrative nucleoside transporters (CNTs).^382^ The ENT family comprises four members (ENT1-4, encoded by *SLC29A1-4*), which function as sodium-independent transporters with specific substrate selectivity. In contrast, the CNT family contains three members (CNT1-3, encoded by *SLC28A1-3*) that operate via sodium gradient-dependent transport mechanisms and exhibit bidirectional movement with defined substrate specificity.^382^

Both ENTs and CNTs play crucial roles in nucleotide synthesis, purinergic signaling, and cellular uptake of nucleoside-derived pharmaceuticals.^383^ Their importance in osteoclast differentiation has been experimentally verified, as disruption of either hENTs or hCNTs impairs gene transcription, protein synthesis, and terminal osteoclast differentiation.^384^ The dual ENT/CNT inhibitor SUKU-33 prevents adenosine uptake and suppresses osteoclast-related gene expression, thereby inhibiting osteoclastogenesis.^385^ Among these transporters, ENT1 serves as the principal equilibrative nucleoside transporter responsible for cytoplasmic uptake of purines and adenosine.^386^ Studies in both human and murine models demonstrate ENT1’s critical involvement in osteoclast differentiation and bone resorption.^387,388^ Pharmacological inhibition of ENT1 not only reduces adenosine uptake but also correlates with decreased bone mineral density,^385^ underscoring its fundamental role in bone metabolism.

### Glutamate transporter

Glutamate serves not only as a crucial neurotransmitter in the central nervous system but also plays a significant role in bone metabolism regulation. Glutamate transporters are categorized into three distinct groups: (1) high-affinity glutamate transporters comprising EAAC1 (SLC1A1), GLT1 (SLC1A2), GLAST (SLC1A3), EAAT4 (SLC1A6), and EAAT5 (SLC1A7); (2) neutral amino acid transporters (ASCT1/SLC1A4 and ASCT2/SLC1A5); and (3) vesicular glutamate transporters (VGLUT1-3)^389^.

Glutamate transporters function as active transport systems that clear glutamate from synaptic clefts, ensuring precise regulation of neurotransmission and maintaining synaptic integrity. Conversely, plasma membrane glutamine transporters facilitate cellular glutamine uptake to meet intracellular demands, with glutamine synthetase activity being particularly elevated in highly proliferative cells, such as metabolically active cells and malignant cells.^390^ In bone homeostasis, glutamine serves as a critical metabolic substrate, providing energy for protein and nucleic acid synthesis through the tricarboxylic acid cycle. This amino acid actively promotes the proliferation and differentiation of osteoblasts, chondrocytes and osteoclasts. The enzyme glutaminase catalyzes the deamination of glutamine to glutamate, and its deficiency in osteoblasts and chondrocytes results in impaired osteoblast formation and reduced bone mass, potentially leading to osteoporosis.^391^ Within osteoclasts, glutamine represents a vital metabolic fuel for biosynthetic processes. The transporter ASCT2 plays a pivotal role in glutaminolysis and mTOR signaling pathways. During osteoclast differentiation, ASCT2 expression is upregulated to facilitate glutamate import, serving both as a carbon source and to meet the heightened demand for oxidative phosphorylation and cysteine-related metabolic processes.^376^ Furthermore, ASCT2 may influence monocyte cell fusion through interactions with human endogenous retroviral syncytin-1, a mechanism essential for osteoclast fusion.^392^

The functional roles of GLAST and VGLUT1 in osteoclasts have been characterized. GLAST is predominantly localized to the osteoclast plasma membrane, where they regulate extracellular glutamate concentration. This regulation is critical for receptor binding and modulates intracellular glutamate signaling through complex mechanisms.^393^ VGLUT1, expressed in osteoclasts, facilitates L-glutamate secretion via transcytosis. Genetic ablation of VGLUT1 results in suppressed L-glutamate secretion and enhanced bone resorption activity.^394^ Glutamate mediates IL-17-induced osteoclast differentiation and participates in energy metabolism regulation. These findings suggest that targeting the IL-17-glutamate-energy metabolism axis may represent a novel therapeutic approach for osteoporosis treatment.^395^

### Cationic amino acids transporter

The cationic amino acid transporters, encoded by the *SLC3A* and *SLC7A* gene families,^396^ play crucial roles in numerous biological processes, including amino acid and carnitine uptake, multidrug resistance, and immune responses.^397,398^ The SLC7 family comprises two distinct subgroups: the cationic amino acid transporters (CATs, including SLC7A1-4 and SLC7A14) and the light/catalytic subunits of heteromeric amino acid transporters (HATs, encompassing L-type amino acid transporters (LATs, SLC7A5-13 and SLC7A15).^399^ These HATs form functional complexes with their associated heavy subunits (glycoproteins), either 4F2hc (SLC3A2) or rBAT (SLC3A1), which constitute the SLC3A family.^400^

CAT1 appears to enhance cell-cell fusion efficiency in osteoclasts.^401^ Predominantly expressed in osteosarcoma cells, CAT1 is associated with various malignant tumor characteristics. It regulates both tumor cell-macrophage interactions and macrophage functionality through multiple pathways. Cepharanthine, a potential CAT1 inhibitor, may represent a viable therapeutic strategy for osteosarcoma treatment.^402^

Amino acid substrates transported by LAT1 exert both direct and indirect beneficial effects on bone health, indicating a potential role for LAT1 in bone homeostasis.^403^ Encoded by *SLC7A5*, LAT1 suppresses osteoclastogenesis and maintains bone homeostasis via the mTORC1 pathway.^404^ Osteoclast-specific *Slc7a5* deletion in mice resulted in osteoclast activation and bone loss in vivo, while in vitro studies demonstrated enhanced osteoclastogenesis and impaired mTORC1 pathway activation in *Slc7a5*-deficient cells. Genetic activation of mTORC1 rescued both the excessive osteoclastogenesis and bone loss observed in *Slc7a5*^*−/−*^ mice. Furthermore, *Slc7a5* deficiency elevated the expression of NFATc1, a key regulator of osteoclast function, and promoted its nuclear accumulation, potentially through the canonical NF-κB pathway and Akt-GSK3β signaling axis, respectively. These findings establish the LAT1-mTORC1 axis as a critical regulator of bone resorption and homeostasis through NFATc1 modulation in osteoclasts, revealing a molecular link between amino acid metabolism and skeletal integrity.^404^

### Vitamin C transporter

Vitamin C is an essential nutrient for physiological development. In mammals, its uptake and distribution depend on specific transporter systems. Most cells express two distinct vitamin C transporter mechanisms: sodium-coupled vitamin C transporters (SVCTs), which specifically transport ascorbic acid (the reduced form of vitamin C), and GLUTs, which transport dehydroascorbic acid (the oxidized form of vitamin C).^405^ The SVCT family, encoded by the *SLC23A* gene, comprises four isoforms exhibiting distinct functional properties and tissue-specific expression patterns.^406^

The role of vitamin C in suppressing osteoclast differentiation through reduced expression of RANK, c-Fos and c-Jun has been established.^407^ However, recent studies demonstrate that vitamin C may also stimulate osteoclast differentiation by enhancing precursor cell formation, fusion, and longevity.^408^ During bone resorption, osteoclasts dissolve mineralized bone matrix at resorption sites, releasing substantial quantities of calcium and phosphate ions into the extracellular fluid. Current evidence suggests that Ca^2+^ and PO₄³^−^ ions are necessary for SVCT2 and osteopontin (OPN) expression in osteoblasts following osteoclastic degradation. Notably, PO₄³^−^ induces SVCT2 and OPN expression, as well as OPN promoter activity, in a dose-dependent manner.^409^ Nevertheless, the specific function of SLC23A2 in osteoclast differentiation and bone resorption remains unclear.

## Part VI ATP-binding cassette transporter

The ATP-binding cassette (ABC) transporter superfamily represents one of the largest classes of membrane proteins. Structurally and functionally, ABC transporters comprise four core domains: two transmembrane domains that form a unidirectional pore responsible for substrate efflux from the cytoplasm, and two nucleotide-binding domains which bind and hydrolyze ATP to provide energy.^410^ These transporters are classified into seven principal subfamilies (ABCA to ABCG) based on their functional characteristics, tissue distribution, and domain organization, with each subfamily exhibiting distinct expression patterns across different species and tissues.^410^ ABC transporters are distributed throughout various cellular membranes, including the plasma membrane and intracellular organelles, where the B subgroup localizes to mitochondria and the D subgroup to peroxisomes.^411^ They play crucial roles in multiple cellular processes such as signal transduction pathways, energy provision, metabolite elimination, multidrug resistance, cellular differentiation, cell migration, and cell fusion.^412^ The biological functions of ABC transporters are primarily mediated through ATP-dependent transmembrane efflux mechanisms, facilitating the transport of diverse substrates including pharmaceuticals, cyclic nucleotides,^413^ chloride ions, cholesterol, iron, and other organic anions.^414^

Several ABC transporters have been implicated in osteoclast biology. ABCB4 and ABCG1 function as lipid-related proteins that participate in osteoclastogenesis by affecting cell-cell fusion of mononuclear pre-osteoclasts.^415^ These ABC proteins integrated in the plasma membrane facilitate lipid transport between the outer and inner leaflets of the lipid bilayer. Specifically, ABCB4 and ABCG1 mediate phosphatidylethanolamine translocation to the external surface, significantly influencing osteoclast formation, particularly during the final cell-cell fusion stage of osteoclastogenesis. Knockdown of these transporters reduces osteoclast fusion and differentiation.^415^ ABCG1 additionally contributes to cholesterol homeostasis. High-density lipoprotein 3 (HDL3) upregulates ABCG1 expression and promotes cholesterol efflux from osteoclasts. Disruption of cholesterol homeostasis enhances osteoclast apoptosis.^416^ ABCC5 supports osteoclast formation and promotes breast cancer bone metastasis. It is overexpressed in breast cancer osseous metastases compared to primary tumors.^417^ Stable ABCC5 knockdown substantially reduced bone metastatic burden and osteolytic destruction in murine models. Conditioned media from ABCC5-deficient breast cancer cells failed to induce in vitro osteoclastogenesis, unlike media from ABCC5-expressing cells.^417^ This confirms ABCC5’s role in supporting osteoclast formation and facilitating breast cancer bone metastasis. While the functions of ABCB4, ABCG1, and ABCC5 in osteoclasts have been partially characterized, the roles of other abundant ABC transporters in osteoclast biology remain unclear and require further investigation.

## Perspective

### Abundant ion channels with functions

**Fig. 5:**
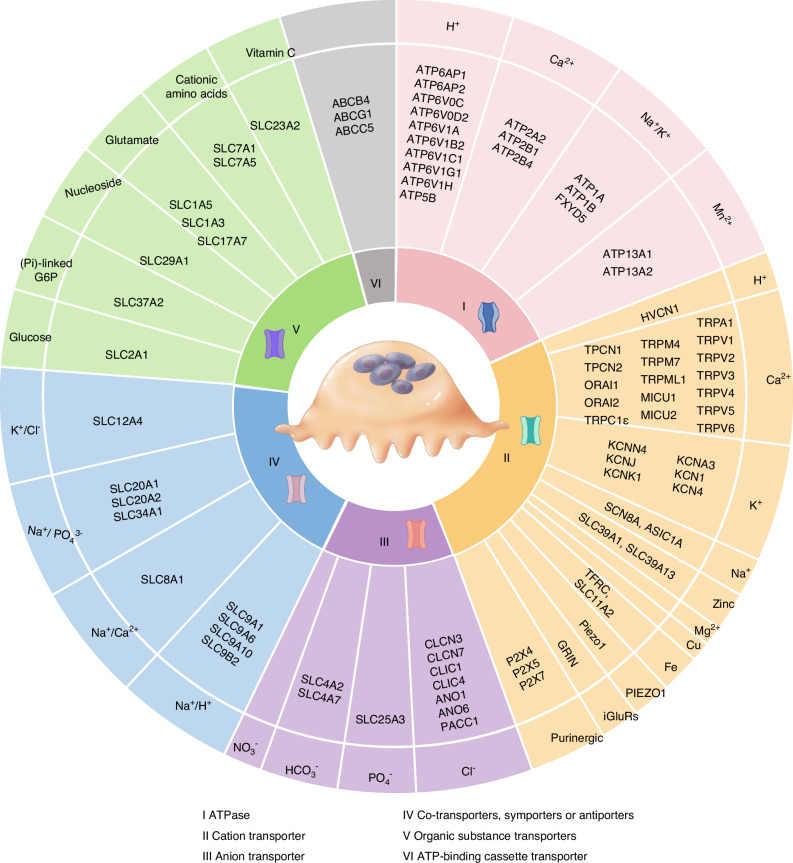
Ion Channels and Transporters with established functional roles in osteoclast biology. There are ~90 ion channels and transporters with well-established research in osteoclasts

Osteoclasts express a diverse repertoire of functionally essential ion channels and transporters. This review systematically analyzes approximately 90 genes with established functional roles in osteoclast biology (Table 1, Fig. 5).

**Table 1:** Summary Table of Ion Channels and Transporters with Established Functional Roles in Osteoclast Biology

Part	Section	Sub-section	Gene
Part I ATPase	Proton transporting ATPase	ATPase, lysosomal	*ATP6AP1, ATP6AP2, ATP6V0C, ATP6V0D2, ATP6V1A, ATP6V1B2, ATP6V1C1, ATP6V1G1, ATP6V1H*
		ATPase, mitochondrial	*ATP5B*
	Calcium transporting ATPase	Sarco/endoplasmic reticulum calcium ATPase	*ATP2A2*
		Plasma membrane calcium ATPase	*ATP2B1, ATP2B4*
	Sodium/potassium transporting ATPase		*ATP1A, ATP1B, FXYD*^*a*^
	Manganese transporting ATPase		*ATP13A1, ATP13A2*
Part II Cation transporter	Proton channel		*HVCN1*
	Calcium channel	Voltage gated calcium channel	*CACN*^*a*^
		Ligand gated calcium channel- IP3 receptor, ryanodine receptor, two-pore channel	*ITPR*^*a*^*, RYR*^*a*^*, TPCN1, TPCN2*
		Ligand gated calcium channel- calcium release activated calcium channel	*ORAI1, ORAI2*
		Ligand gated calcium channel- transient receptor potential channel	*TRPC1ε, TRPA1, TRPV1, TRPV2, TRPV3, TRPV4, TRPV5, TRPV6, TRPM4, TRPM7, TRPML1*
		Mitochondrial calcium uniporter	*MICU1, MICU2*
	Potassium channel	Calcium-activated potassium channel	*KCNN4*
		Inwardly rectifying potassium channel	*KCNJ*^*a*^
		Tandem pore domain potassium channel	*KCNK1*
		Voltage-gated potassium channel	*KCNA3*
		Hyperpolarization activated cyclic nucleotide-gated potassium channel	*HCN1, HCN4*
	Sodium channel	Voltage-gated sodium channel	*SCN8A*
		Epithelial sodium channel	*ASIC1A*
	Zinc transporter		*SLC39A1, SLC39A13*
	Magnesium transporter		^*b*^
	Copper transporter		^*b*^
	Iron transporter		*TFRC, SLC11A2*
	Piezo-type mechanosensitive ion channel		*PIEZO1*
	Ionotropic glutamate receptor		*GRIN*^*a*^
	Purinergic receptor		*P2X4, P2X5, P2X7*
Part III Anion transporter	Chloride channel	Voltage-gated chloride channel	*CLCN3, CLCN7*
		Chloride intracellular channel	*CLIC1, CLIC4*
		Calcium-activated chloride channel	*ANO1, ANO6*
		Proton-activated chloride channel	*PACC1*
	Phosphate transporter		*SLC25A3*
	Bicarbonate transporter		*SLC4A2, SLC4A7*
	Nitrate transporter		^*b*^
Part IV Cotransporters, symporters or antiporters	Sodium/hydrogen exchanger		*SLC9A1, SLC9A6, SLC9A10, SLC9B2*
	Sodium/calcium exchanger		*SLC8A1*
	Sodium/phosphate cotransporter		*SLC20A1, SLC20A2, SLC34A1*
	Potassium/chloride cotransporter		*SLC12A4*
Part V Organic substance transporters	Glucose transporter		*SLC2A1*
	(Pi)-linked G6P anti-porter		*SLC37A2*
	Nucleoside transporter		*SLC29A1*
	Glutamate transporter		*SLC1A5, SLC1A3, SLC17A7*
	Cationic amino acids transporter		*SLC7A1, SLC7A5*
	Vitamin C transporter		*SLC23A2*
Part VI ATP-binding cassette transporter			*ABCB4, ABCG1, ABCC5*

^a^Osteoclast-related researches focus on the entire channel protein rather than individual subunits

^b^Only translocated substances research about osteoclasts

Current research primarily concentrates on V-ATPase, cation channels (including hydrogen transporters, calcium channels and potassium channels), and anion channels such as chloride, and phosphate channels, with their functions in osteoclasts confirmed through human or animal models. Notably, V-ATPase and TRP channels have emerged as particularly prominent research targets, highlighting the critical importance of acidification and calcium transport in osteoclast-mediated bone resorption. These channels demonstrate intricate functional relationships, exemplified by the coordinated activity of V-ATPase and chloride channels at the ruffled border, where they synergistically transport H^+^ and Cl^−^ to establish and maintain the acidic microenvironment essential for bone resorption.

Although the ion channel and transporter families comprise an extensive array of members, current research on such molecules in osteoclasts remains limited to a few members. As such, there is substantial room for significant advancement in both the breadth and depth of relevant investigations. The transcriptional process represents a core step in the execution of gene function, and the gene expression levels of ion channels and transporters in osteoclasts can serve as a crucial basis for screening novel research targets. Meanwhile, the biological functions and regulatory mechanisms of numerous known ion channels and transporters in osteoclasts have not yet been fully elucidated, which needs more systematic and in-depth mechanistic studies.

### Locations beyond the ruffled border

**Fig. 6:**
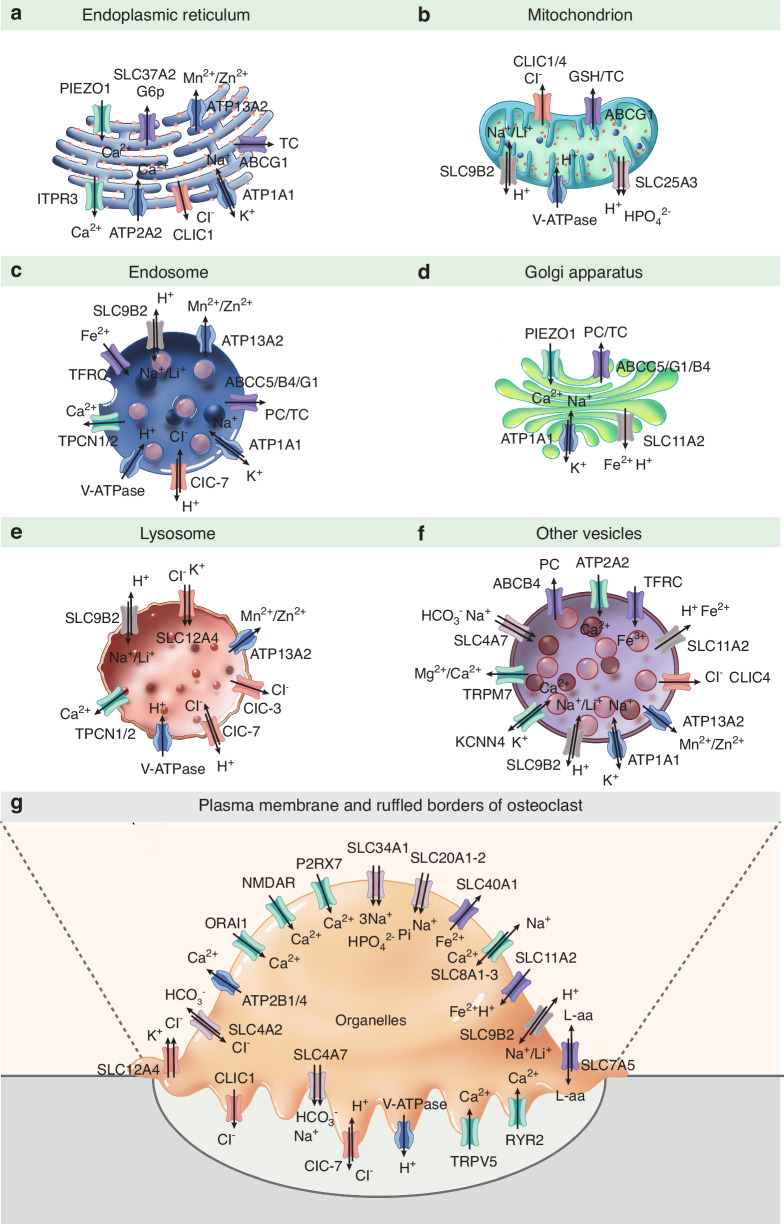
Localization and transport characteristics of ion channels and transporters in osteoclasts. **a** Endoplasmic reticulum membrane: ATP1A1, ATP2A2, CLIC1, ITPR3, PIEZO1, SLC37A2, ATP13A2, and ABCG1. **b** Mitochondrial membrane: V-ATPase, SLC25A3, SLC9B2, CLIC1/4, and ABCG1. **c** Endosome membrane: ClC-7, V-ATPase, ATP1A1, TPCN1/2, TFRC, SLC9B2, ATP13A2, and ABCC5/B4/G1. **d** Golgi apparatus membrane: ATP1A1, PIEZO1, SLC11A2, and ABCC5/G1/B4. **e** Lysosome membrane: ClC-7, ClC-3, V-ATPase, TPCN1/2, SLC9B2, SLC12A4, and ATP13A2. **f** Vesicle membrane: ATP1A1, KCNN4, ATP2A2, TRPM7, SLC4A7, ATP13A2, SLC9B2, TFRC, SLC11A2, CLIC4, and ABCB4. **g** Plasma membrane and ruffled border: Key channels and transporters essential for acid secretion and ion homeostasis. Data for organelle-localized channels/transporters were obtained from NCBI database (https://www.ncbi.nlm.nih.gov/); data for plasma membrane and ruffled border channels/transporters were derived from published literature

The diverse array of channels in osteoclasts is reflected in their ability to transport ions and other substances, as discussed in this review. In addition to their transport functions, their varied localization is also of significant interest. Only a few channels, such as V-ATPase, ClC-7, SLC4A7, CLIC1, TRPV5, and RYR2, are localized at the ruffled border, where they play a key role in maintaining acidification within the resorption bay. Organelles enriched with channels and transporters also contribute critically to osteoclast function. Some regulate pH in lysosomes and vesicles, while others are distributed across the plasma membrane or the membranes of organelles such as the endoplasmic reticulum, Golgi apparatus, and mitochondria. These molecules are also found on the nucleoplasmic membrane. The localization and transport characteristics of ion channels and transporters in osteoclasts are illustrated in Fig. 6. This spatial diversity may provide insights into the more complex functional roles of ion channels in osteoclasts.

### Ion coordination in osteoclast development and function

The development and functional regulation of osteoclasts constitute a complex process requiring dynamic coordination of multiple ion channels and transport proteins. These proteins establish an intricate regulatory network that governs osteoclast differentiation, polarization, bone resorption and apoptosis through modulation of ion flux, membrane potential, pH homeostasis and signal transduction.

#### Ion regulation in osteoclast development

Osteoclast precursors on the bone surface are stimulated by macrophage colony-stimulating factor and receptor activator of nuclear factor kappa-Β ligand (RANKL) secreted by osteoblast lineage cells, leading to activation of the immediate-early transcription factors NF-κB and c-Fos and driving osteoclastogenesis.^418^ The activation of RANK initiates osteoclastogenesis, triggering phosphoinositide-specific phospholipase C (PLC) activation, membrane hydrolysis of phosphatidylinositol 4,5-bisphosphate (PIP₂) to form diacylglycerol and inositol trisphosphate (IP₃), IP₃-mediated calcium ion (Ca^2+^) release, and subsequent activation of the calcium-dependent phosphatase calcineurin.^419^ The activated calcineurin dephosphorylates and thereby activates NFATc1. NFATc1 activates the expression of multiple genes related to osteoclastogenesis, including the membrane fusion-promoting factor DC-STAMP, the actin ring component β3 integrin, and bone-degrading hydrolases.^420,421^ Numerous ion channels participate in calcium transport within osteoclasts. The Ca^2+^ signal initiates the differentiation process. The CRAC channel, composed of ORAI1 and STIM1 (ORAI1/STIM1), mediates the influx of extracellular Ca^2+^^268^, enhancing the nuclear translocation of NFATc1. Ano5 enhances osteoclast differentiation through promoting Akt activation.^296,297^ KCNN4 regulates Ca^2+^ oscillation,^133^ promoting the fusion of macrophages and the formation of multinucleated osteoclasts. KCNK1 deficiency increases K^+^ influx and negatively regulates osteoclast differentiation by inhibiting Ca^2+^ oscillation and the JNK-NFATc1 axis.^134^

The mature osteoclast is characterized by the formation of a sealing zone and ruffled border. Osteoclasts adhere to bone surfaces via sealing zones, which demarcate resorption areas.^422^ Following the formation of the sealing zone, two specialized membrane domains develop: the ruffled border within the sealing zone and the functional secretory domain on the opposite side. Osteoclasts exhibit two distinct actin cytoskeletal structures: podosomes on non-mineralized substrates and sealing zones on mineralized extracellular matrices.^423^ The podosome consists of two functionally distinct actin subdomains: a core composed of branched actin filaments and an actin cloud. Through complete reorganization of these subdomains, mature bone-resorbing osteoclasts ultimately form the sealing zone. The TRPV channel participates in actin ring assembly, with SLC8A1 and SLC39A1 also playing regulatory roles.^118^ TRPV5 mediates Ca^2+^ influx, activating calcium-binding proteins and promoting actin ring formation. NCX1 (SLC8A1) regulates Na^+^/Ca^2+^ exchange and promotes osteoclast differentiation by modulating the JNK/c-Fos/NFATc1 signaling pathway.^360^ ZIP1 (SLC39A1) localizes near actin, inhibiting NF-κB activity to prevent excessive bone resorption.^175^

The cross-cell ion transport network and its regulation in bone resorption involve several key processes. Acidification represents an essential prerequisite for bone resorption, with multiple channels participating in this process. During acidification, TCIRG1 cooperates with Rab7/Rab27A GTPases to direct proton pumps to the ruffled membrane.^26^ The CLCN7-Ostm1 complex colocalizes with V-ATPase through vesicular transport, forming an acidification functional unit.^268^ Reduced V-ATPase activity induces cytoplasmic alkalization,^10^ while NHE10 (SLC9A10) maintains basolateral membrane pH,^351^ facilitating cellular detachment from the bone surface. RANKL-induced PACC1 regulates endosomal pH to promote lysosomal fusion. During calcium hydroxyapatite absorption, Ca^2+^ and Pi are transported intracellularly via TRPV5 and TRPV6 in the resorption lacuna, subsequently being excreted into the bloodstream through NCX1 (SLC8A1) and PMCA (ATP2B).^49,424,425^ SLC34A1 and SLC20A1/2 mediate Pi reabsorption for cellular metabolism.^364^ Low-amplitude mechanical stimulation activates PIEZO1, inducing Ca^2+^ release from the endoplasmic reticulum via the PLC-IP3 pathway.^228,230,426^ This mechanism sustains Ca^2+^ oscillations while preventing excessive osteoclast activation. Conversely, high-amplitude stimulation promotes bone destruction through the Piezo1-Ca^2+^-NF-κB axis.^229,230^ The apoptotic phase and bone remodeling coupling involve distinct regulatory mechanisms. Disruption of Ca^2+^ homeostasis initiates apoptosis. Following bone resorption completion, TRPV5-mediated Ca^2+^ influx diminishes while ATP2B4 pump activity increases, terminating intracellular Ca^2+^ oscillations and triggering caspase-dependent apoptosis.^427^

#### Ion balance of osteoclasts

The H^+^-Cl^−^ coacidification process involves three coordinated mechanisms: (1) charge balance between V-ATPase and CLC-7, (2) intracellular pH buffering by SLC4A2 and CA2, and (3) fine pH regulation by PAC and NHE10. V-ATPase pumps H^+^ into the resorption lacuna (pH~4.5) via ATP hydrolysis, with its electrogenic effect neutralized by the ClC-7-Ostm1 complex-mediated 2Cl^−^/H^+^ exchange.^353^ This complex is coexpressed through MITF transcription factor regulation. Mutations in either *TCIRG1* or *CLCN7* cause osteopetrosis due to failed lacunar acidification. By generating protons through the catalysis of CO₂ and H₂O to H^+^ and HCO_3_^−^, CA2 provides the substrate necessary for V-ATPase activity. SLC4A2 maintains intracellular pH stability through Cl^−^/HCO₃^−^ exchange, extruding HCO₃^−^ while importing Cl^−^. SLC4A2 deficiency increases osteoclast apoptosis and causes abnormal actin ring depolymerization, leading to osteopetrosis.^354^ RANKL/NFATc1-induced PAC regulates endosomal pH to enable lysosome-ruffled border membrane fusion. NHA2 (SLC9B2), localized to basolateral membranes, functions as a mitochondrial Na^+^/H^+^ exchanger that modulates both mitochondrial pH and Na^+^-dependent swelling, thereby meeting V-ATPase’s energy demands.^355^

The K^+^-Cl^−^ transport system regulates osteoclast membrane potential. The BK channel maintains membrane hyperpolarisation by mediating K^+^ efflux,^144^ thereby enhancing V-ATPase proton-pumping efficiency. KCC1 (SLC12A4) facilitates electroneutral K^+^-Cl^−^ cotransport,^369^ which reduces intracellular Cl^−^ concentration and indirectly supports CIC-7-mediated Cl^−^ influx into the resorption lacuna. Pharmacological inhibition of KCC1 with DIOA diminishes both H^+^ secretion and bone resorptive capacity.^372^

Ca^2+^ signaling regulation operates through two principal mechanisms: intracellular flux and extracellular excretion. TRPV5 localized to the apical membrane mediates Ca^2+^ influx from resorbed bone matrix, thereby modulating NFATc1 activity.^118^ Genetic ablation of *Trpv5* results in increased osteoclast numbers but impaired resorptive capacity.^118^ The purinergic receptor P2X7 responds to extracellular ATP by promoting Ca^2+^ influx and facilitating osteoclast fusion.^248,249^ NMDA receptors (GRIN family) maintain cellular viability through glutamate-dependent NF-κB activation. At the basolateral membrane,^237^ ATP2B1/4 extrudes Ca^2+^ via ATP hydrolysis,^45^ while NCX1 (SLC8A1) couples Ca^2+^ efflux to the Na^+^ electrochemical gradient, collectively preventing cytotoxic Ca^2+^ accumulation.^358–360^ Intracellular Ca^2+^ homeostasis is further buffered by endoplasmic reticulum^268^ and mitochondrial storage compartments,^129^ which regulate Ca^2+^ fluctuations.

## Potential therapeutic targets

The specific localization of ion channels and transporters on osteoclast membranes facilitates a complex signaling network that regulates bone resorption. This system is crucial for maintaining ionic homeostasis and offers a range of potential drug targets for treating bone metabolic diseases.

### Inhibitors targeting various V-ATPase subunits

The V-ATPase, a multi-subunit proton pump that is highly expressed in osteoclasts, is essential for bone resorption through its role in acidifying the resorption lacunae, representing a promising target for anti-osteoporotic therapy. Our group has previously reviewed inhibitors targeting various V-ATPase subunits.^10^ Broad-spectrum V-ATPase inhibitors, such as bafilomycin A1, concanamycin A, and archazolid, target the V₀ domain to block proton translocation.^428,429^ Although these compounds are potent inhibitors of bone resorption in vitro, their systemic use is limited by on-target toxicity, a consequence of the ubiquitous expression of V-ATPases. To improve specificity, several osteoclast-selective inhibitors have been developed. SB242784, an indole derivative structurally optimized from bafilomycin, exhibits nanomolar potency against the osteoclast V-ATPase and prevents bone loss in ovariectomised rats.^430^ Other selective agents, including FR167356, FR202126, and FR177995, suppress osteoclast resorption in a dose-dependent manner. Naturally derived molecules also show therapeutic potential.^431^ Diphyllin inhibits V-ATPase-dependent lysosomal acidification and impairs resorption by human osteoclasts.^432^ Similarly, saliphenylhalamide (saliPhe) attenuates particle-induced osteolysis in mice via a V₀-targeting mechanism that is distinct from that of macrolide inhibitors.^433,434^ Alternative strategies focus on disrupting interactions between V-ATPase subunits. KM91104 interferes with the a3–B2 interaction, impairing V-ATPase trafficking to the ruffled border without affecting cell viability.^435^ The flavonoid luteolin inhibits resorption by disrupting the a3–d2 interaction, which confirms that targeting subunit interactions is a viable therapeutic route.^436–438^

Recent years have witnessed significant progress in developing pharmacological agents that target V-ATPase, with several emerging compounds demonstrating therapeutic potential across various diseases. Notably, the macrocyclic lactone verucopeptin was identified as a selective V-ATPase inhibitor. It suppresses mTORC1 signaling by binding to the V1G subunit and shows promising activity against multidrug-resistant cancers.^439^ Conversely, nicotinamide mononucleotide (NMN) augments V-ATPase function by promoting the assembly of its V_1_ and V_0_ domains, thereby maintaining endosomal acidification and ameliorating lipotoxic cardiomyopathy in diabetic models.^440^ Beyond human therapeutics, the agricultural sector has seen the development of selective V-ATPase inhibitors. These include fluopicolide, which specifically targets oomycete pathogens,^441^ and periplocoside P, an insecticidal compound that acts on the A subunit.^442^

In summary, V-ATPase represents a promising target for inhibiting bone resorption. Although the development of osteoclast-specific inhibitors and subunit interaction disruptors has enhanced targeting precision, several challenges must be addressed in future research. Notably, the role of V-ATPase activation in bone remodeling requires clarification, particularly given the bone loss phenotypes associated with deficiencies in specific subunits such as subunit H. Consequently, next-generation drug development must not only focus on optimizing the selectivity, delivery, and safety profiles of V-ATPase inhibitors but also account for their potential effects on osteoblasts and systemic bone remodeling processes.

### Other ion channel modulators (Table 2)

#### TRP channels and calcium-related targets

The TRP channel family constitutes a promising target class. HC030031 specifically suppresses TRPA1 activity,^443^ whereas AMG-517 and AMG-9810 antagonize TRPV1.^444,445^ Tranilast inhibits TRPV2^446^, and the natural compound carvacrol activates TRPV3. ^447^ For TRPV4, both an inhibitor (RN-1734) and an activator (RN-1747) have been identified, enabling its bidirectional modulation.^448^ TRPV6 is blocked by phenyl-cyclohexyl-piperazine cis-22a,^449^ and TRPM7 is inhibited by NS85939.^450^ These agents show promise for managing pain, inflammation, and disorders of calcium homeostasis relevant to bone metabolism.

**Table 2:** Inhibitors and activators of channels and transporters^a^

Channel or Protein Name	Gene	Inhibitor	Activator	Reference
ATP1A1	*ATP1A1*		Mannitol and gemfibrozil	Ref. ^472^
SERCA	*ATP2A1-3*	Thapsigargin		Ref. ^44^
Cav1.3	*CACNA1D*		Ionomycin	Ref. ^82^
ATP2A2	*ATP2A2*	Thapsigargin		Ref. ^473^
SPCA1	*ATP2C1*	Bis(2-hydroxy-3-tert-butyl-5-methyl-phenyl)-methane (bis-phenol)		Ref. ^451^
ATP5D	*ATP5D*		Phoenixin 20	Ref. ^453^
ATP5E	*ATP5E*	Oligomycin A		Ref. ^452^
CLIC4	*CLIC4*	Amphotericin B and rapamycin		Ref. ^460^
KCa3.1	*KCNN4*	Dexamethasone		Ref. ^474^
Kv1.3	*KCNA3*	PAP-1		Ref. ^475^
Kv1.3	*KCNA3*	Fe_2_O_3_ nanoparticles		Ref. ^163^
MICU1	*MICU1*	Ruthenium Red	Spermine	Ref. ^476^
PIEZO1	*PIEZO1*	Ruthenium red, GsMTx4		Ref. ^426^
DMT1	*SLC11A2*	2-(3-carbamimidoylsulfanylmethyl-benzyl)-isothiourea		Ref. ^457^
KCC4	*SLC12A7*	(R)-( + )-[(2-n-butyl-6,7-dichloro-2-cyclopentyl-2,3-dihydro-1-oxo-1H-inden-5-yl)oxy]acetic acid		Ref. ^458^
KCC1	*SLC12A4*	R(+)-butylindazone (DIOA)		Ref. ^372^
ASCT2	*SLC1A5*	L-glutamyl nitroanilides, L-serine ester serine biphenyl-4-carboxylate		Ref. ^454^
SNAT1	*SLC38A1*	2-amino-4-bis(aryloxybenzyl)aminobutanoic acids		Ref. ^455^
AE2	*SLC4A2*	Calmidazolium		Ref. ^461^
CRTR	*SLC6A8*	β-guanidinoproprionic acid		Ref. ^456^
TPC2	*TPCN2*	Naringenin		Ref. ^465^
TRPA1	*TRPA1*	HC030031		Ref. ^443^
TRPC1	*TRPC1*	I-mfa		Ref. ^115^
TRPM7	*TRPM7*	NS8593		Ref. ^450^
TRPV1	*TRPV1*	AMG-517, AMG-9810		Ref.^444,445^
TRPV2	*TRPV2*	Tranilast		Ref. ^446^
TRPV3	*TRPV3*		Carvacrol	Ref. ^447^
TRPV4	*TRPV4*	RN-1734	RN-1747	Ref. ^448^
TRPV6	*TRPV6*	Phenyl-cyclohexyl-piperazine cis-22a		Ref. ^449^
CLCN7	*ClC-7*	PI(3,5)P2		Ref. ^459^
MDR3	*ABCB4*	Perfluorooctane sulfonate		Ref. ^462^
MRP1	*ABCC1*	Alisertib (MLN8237)		Ref. ^463^
ABCE1	*ABCE1*	miR-299-3p		Ref. ^464^
ASIC1a	*ASIC1A*	Hi1a		Ref. ^466^
ENTs and CNTs	*SLC28A1*, *SLC29A1*	SUKU-33		Ref. ^385^

^a^The inhibitors and activators targeting different V-ATPase subunits have been comprehensively reviewed previously^10^ and are therefore not included in the current table

Other calcium-handling proteins also offer important therapeutic targets. Thapsigargin inhibits the sarco/endoplasmic reticulum Ca²^+^-ATPase (SERCA), thereby affecting intracellular calcium storage and signallin.^44^ The Golgi pump SPCA1 (ATP2C1), which is involved in calcium and manganese transport, is blocked by bis-phenol derivatives.^451^ In mitochondrial energy metabolism, the key ATP synthase subunit ATP5E is sensitive to oligomycin A, which disrupts oxidative phosphorylation.^452^ Conversely, ATP5D is activated by phoenixin 20, suggesting a role in regulating energy metabolism.^453^

#### Solute Carrier (SLC) transporters

Pharmacological agents have been developed to target a range of SLC transporters. L-Glutamyl nitroanilides and serine ester derivatives inhibit ASCT2 (SLC1A5), a neutral amino acid transporter for glutamine, implicating its role in cancer metabolism.^454^ SNAT1 (SLC38A1), a system A neutral amino acid transporter, is blocked by 2-amino-4-bis(aryloxybenzyl)aminobutanoic acids.^455^ β-Guanidinoproprionic acid inhibits the creatine transporter CRTR (SLC6A8), thereby affecting cellular energy buffering capacity.^456^ A specific isothiourea derivative blocks DMT1 (SLC11A2), a divalent metal transporter.^457^ For chloride-coupled transporters such as KCC4, inhibitors including R( + )-DIOA and a cyclopentyl-indan acid derivative provide valuable tools for studying cation-chloride cotransport in cellular volume regulation and ion homeostasis.^458^

#### Chloride channels and exchangers

Chloride transport systems constitute a key target class. ClC-7 is inhibited by PI(3,5)P₂, linking it to phosphoinositide signaling.^459^ CLIC4 is inhibited by both amphotericin B and rapamycin, although the precise mechanisms require further elucidation.^460^ SLC4A2 is inhibited by calmidazolium, suggesting interplay with calcium signaling in its regulation.^461^

#### ABC transporters and specialized channels

Several ATP-binding cassette transporters represent important pharmacological targets. Perfluorooctane sulfonate inhibits MDR3 (ABCB4)^462^, while alisertib suppresses MRP1 (ABCC1)^463^. ABCE1, which functions in ribosome biogenesis and translation termination, is downregulated by miR-299-3p, demonstrating the potential of post-transcriptional regulation as a therapeutic strategy.^464^

Other significant channels and transporters of therapeutic interest include two-pore channel TPC2, which regulates endolysosomal function and is inhibited by the flavonoid naringenin.^465^ Acid-sensing ion channel ASIC1a is blocked by Hi1a, a spider venom-derived toxin that exhibits neuroprotective effects.^466^ SUKU-33 broadly inhibits nucleoside transporters (ENTs/CNTs), potentially influencing the efficacy of nucleoside-based therapies.^385^ The P2X7 receptor, a non-selective cation channel pivotal in inflammation and immunity, is antagonized by AZ11645373^467^ and JNJ-47965567.^468^

### Specificity of ion channel-targeting drugs in osteoclasts

The development of drugs targeting osteoclastic ion channels and transporters has yielded compounds with a wide spectrum of specificity, ranging from broad-acting agents to highly selective inhibitors. Despite significant progress, achieving true cellular and molecular specificity remains a central challenge in this field. The specificity of these pharmacological agents varies considerably, and most compounds exhibit broad inhibitory effects across multiple tissues, even though they are being investigated for bone-related applications.

#### Limited specificity of available compounds

Most available inhibitors exhibit broad-spectrum activity rather than selectivity for osteoclasts. This limitation is particularly evident for V-ATPase inhibitors such as bafilomycin A1, concanamycin A, and archazolid. These compounds primarily target the V0 domain, a structure common to V-ATPases found in diverse tissues. Consequently, their systemic administration often induces substantial on-target side effects due to the ubiquitous expression of V-ATPases in vital organs.

Similarly, the majority of inhibitors targeting other channels and transporters demonstrate broad activity profiles. For instance, thapsigargin non-selectively inhibits SERCA pumps across various cell types, and oligomycin A targets the essential process of oxidative phosphorylation by inhibiting ATP synthase. Furthermore, TRP channel inhibitors, including HC030031 (TRPA1), AMG-517/AMG-9810 (TRPV1), tranilast (TRPV2), and NS8593 (TRPM7), are being investigated for numerous applications beyond bone biology, primarily in the contexts of pain and inflammation.

#### Factors limiting specificity

The limited specificity of these pharmacological agents arises from several fundamental challenges:

##### Evolutionary Conservation

The functional domains targeted by many ion channels and transporters are highly conserved across different tissues, which inherently limits drug specificity. A key example is the V0 domain of V-ATPase, whose structural similarity across tissue-specific isoforms is exploited by broad-spectrum inhibitors such as bafilomycin A1. This challenge of evolutionary conservation also extends to calcium channels. Calcium channel blockers, including dihydropyridines (DHPs), phenylalkylamines (PAAs), and benzothiazepines (BTZs), are primarily designed to target cardiovascular tissues. However, osteoclasts also express abundant voltage-gated calcium channels. Consequently, the potential impact of these established cardiovascular drugs on bone metabolism, mediated through off-target effects on osteoclasts, represents a significant and unexplored area of research, underscoring a critical gap in our understanding of drug specificity.

##### Ubiquitous Expression

Molecular targets such as CLIC4, along with numerous SLC and ABC transporters, are expressed in a wide range of cell types. This ubiquitous presence poses a significant challenge to the selective targeting of osteoclast-specific functions.

##### Structural Homology Within Protein Families

The high degree of structural homology among members of protein families, such as TRP channels and SLC transporters, results in a common lack of selectivity. Consequently, inhibitors frequently interact with multiple family members rather than a single, specific target.

### Advancing ion channel-targeted therapeutics in osteoclasts

The development of targeted therapies for osteoclast-related bone diseases is progressing beyond conventional pharmacology, which is often limited by insufficient efficacy and systemic side effects. Current innovative strategies aim to enhance specificity and precision through novel targeting mechanisms and advanced delivery systems.

#### Approaches for improved specificity

Recent research has focused on developing agents with enhanced selectivity. For instance, the indole derivative SB242784, a structural analog of bafilomycin, demonstrates remarkable selectivity for the osteoclast V-ATPase. Similarly, benzamide-based compounds, including FR167356, FR202126, and FR177995, preferentially inhibit the plasma membrane V-ATPase in osteoclasts. Alternative strategies aim to disrupt osteoclast-specific protein-protein interactions rather than directly inhibit catalytic activity. Examples include KM91104, which interferes with the a3-B2 subunit interaction unique to osteoclast V-ATPase, and luteolin, which disrupts the a3-d2 interaction. Both compounds impair V-ATPase trafficking or function while offering potentially superior cellular specificity. Although most available compounds still require further optimization to achieve true osteoclast-specific targeting with minimal off-tissue effects, the development of these more selective agents represents a promising direction for future therapies.

#### Methodologies for drug discovery and delivery

Advances in nanotechnology hold the potential to revolutionize ion channel research in vitro and enable targeted therapeutic delivery through channel modulation in vivo^469^. Conventional pharmacological treatments for osteoporotic bone defects often exhibit limited efficacy, which can be attributed to adverse effects and imprecise delivery. The charged nature of many ion channels makes them amenable to targeting via magnetic aggregation carriers. For instance, a bone-targeting polymeric nanocarrier with magnetic aggregation properties has demonstrated specific targeting to Piezo1.^232^ Such systems could precisely transport activators like Yoda1 to bone defects for localized channel activation. This principle may be extended to design target-specific magnetic carriers for various ion channels, thereby enhancing drug selectivity and tissue targeting. Biomaterials such as cartilage-penetrating nanomaterials and hydrogels offer promising avenues for improving controlled drug release. Intra-articular injections tailored to osteoarthritic tissues could enhance local drug bioavailability while minimizing systemic side effects.^470^

Beyond conventional small-molecule drugs, alternative therapeutic approaches for osteoarthritis (OA) include siRNA, antisense oligonucleotides, adeno-associated virus (AAV) vectors, antibodies, and mRNA-based strategies.^471^ Although gene therapy using AAV vectors has raised concerns regarding potential toxicity at higher doses,^470^ innovative delivery methods may help mitigate these risks.

Furthermore, the rapid advancement of artificial intelligence is accelerating the analysis of ion channel abnormalities. AI enables deep computational simulations to predict and identify potential drug candidates. AI-assisted prediction of channel-drug interactions may overcome conventional screening limitations, despite the generation of numerous non-viable compounds during the process.

### Future perspectives and integrated strategies

The therapeutic potential of ion channel modulation extends beyond bone diseases. For instance, channels such as HCN play critical roles in neurological disorders like epilepsy and intellectual disability. The sensitivity of these conditions to altered channel currents identifies “cation leak” phenomena as promising research targets. Consequently, strategies developed for osteoclast channels may inform therapeutic approaches for sodium, potassium, and calcium channels implicated in neurological pathologies.

Several innovative strategies are converging to advance osteoclast-targeted therapies. The unique bone microenvironment enables targeted delivery through pH-sensitive prodrugs activated within the acidic resorption lacunae and bone-affinity conjugates that utilize bisphosphonates to enhance localization. Advances in structural biology, particularly cryo-electron microscopy, are facilitating rational drug design by elucidating the molecular determinants of specificity in complexes such as the a3-containing V-ATPase bound to selective inhibitors. Additionally, multi-target strategies that address functional redundancy among ion channels offer a promising alternative to single-target inhibition.

In conclusion, advancing osteoclast ion channel therapeutics necessitates an integrated approach combining microenvironment-targeted delivery, structure-guided optimization, and systems-level modulation. The convergence of these strategies, informed by cross-disciplinary insights from neurology and related fields, provides a comprehensive framework for developing specific and effective treatments for bone metabolic diseases with minimal off-target effects. Future success will depend on continued innovation in targeting technologies and a deepened understanding of osteoclast biology.

## References

[1] Williams, A. J. et al. Iodothyronine deiodinase enzyme activities in bone. *Bone* **43**, 126–134 (2008). 18468505 10.1016/j.bone.2008.03.019 PMC2681075

[2] Komarova, S. V., Dixon, S. J. & Sims, S. M. Osteoclast ion channels: potential targets for antiresorptive drugs. *Curr. Pharm. Des.* **7**, 637–654 (2001). 11375773 10.2174/1381612013397799

[3] Coxon, F. P. & Taylor, A. Vesicular trafficking in osteoclasts. *Semin. Cell Dev. Biol.* **19**, 424–433 (2008). 18768162 10.1016/j.semcdb.2008.08.004

[4] Francis, M. J. O. et al. ATPase pumps in osteoclasts and osteoblasts. *Int. J. Biochem. Cell Biol.* **34**, 459–476 (2002). 11906818 10.1016/s1357-2725(01)00142-x

[5] Henriksen, K. et al. Ion transporters involved in acidification of the resorption lacuna in osteoclasts. *Calcif. Tissue Int.* **83**, 230–242 (2008). 18787885 10.1007/s00223-008-9168-8

[6] Graves, A. R., Curran, P. K., Smith, C. L. & Mindell, J. A. The Cl-/H+ antiporter ClC-7 is the primary chloride permeation pathway in lysosomes. *Nature* **453**, 788–792 (2008). 18449189 10.1038/nature06907

[7] Michalak, A., Wdowikowska, A. & Janicka, M. Plant plasma membrane proton pump: one protein with multiple functions. *Cells*. **11**, 4052 (2022). 10.3390/cells11244052 PMC9777500 36552816

[8] Kuhlbrandt, W. Structure and mechanisms of F-Type ATP synthases. *Annu. Rev. Biochem.* **88**, 515–549 (2019). 30901262 10.1146/annurev-biochem-013118-110903

[9] Vasanthakumar, T. & Rubinstein, J. L. Structure and roles of V-type ATPases. *Trends Biochem.Sci.* **45**, 295–307 (2020). 32001091 10.1016/j.tibs.2019.12.007

[10] Duan, X., Yang, S., Zhang, L. & Yang, T. V-ATPases and osteoclasts: ambiguous future of V-ATPases inhibitors in osteoporosis. *Theranostics* **8**, 5379–5399 (2018). 30555553 10.7150/thno.28391 PMC6276090

[11] Hemken, P., Guo, X. L., Wang, Z. Q., Zhang, K. & Gluck, S. Immunologic evidence that vacuolar H^+^ ATPases with heterogeneous forms of Mr = 31 000 subunit have different membrane distributions in mammalian kidney. *J. Biol. Chem.* **267**, 9948–9957 (1992). 1533641

[12] Stransky, L., Cotter, K. & Forgac, M. The function of V-ATPases in cancer. *Physiol. Rev.* **96**, 1071–1091 (2016). 27335445 10.1152/physrev.00035.2015 PMC4982037

[13] Bouche, V. et al. Drosophila Mitf regulates the V-ATPase and the lysosomal-autophagic pathway. *Autophagy* **12**, 484–498 (2016). 26761346 10.1080/15548627.2015.1134081 PMC4835958

[14] Lee, B. S., Gluck, S. L. & Holliday, L. S. Interaction between vacuolar H^+^-ATPase and microfilaments during osteoclast activation. *J. Biol. Chem.* **274**, 29164–29171 (1999). 10506172 10.1074/jbc.274.41.29164

[15] Lee, S. et al. v-ATPase V0 subunit d2-deficient mice exhibit impaired osteoclast fusion and increased bone formation. *Nat. Med.* **12**, 1403–1409 (2006). 17128270 10.1038/nm1514

[16] Wu, H., Xu, G. & Li, Y. Atp6v0d2 is an essential component of the osteoclast-specific proton pump that mediates extracellular acidification in bone resorption. *J. Bone. Miner. Res.* **24**, 871–885 (2009). 19113919 10.1359/JBMR.081239 PMC2672205

[17] Sun-Wada, G. et al. Acidic endomembrane organelles are required for mouse postimplantation development. *Dev. Biol.* **228**, 315–325 (2000). 11112332 10.1006/dbio.2000.9963

[18] Bhargava, A. et al. Osteopetrosis mutation R444L causes endoplasmic reticulum retention and misprocessing of vacuolar H^+^-ATPase a3 subunit. *J. Biol. Chem.* **287**, 26829–26839 (2012). 22685294 10.1074/jbc.M112.345702 PMC3411020

[19] Xue, Y. et al. Dental abnormalities caused by novel compound heterozygous CTSK mutations. *J. Dent. Res.* **94**, 674–681 (2015). 25731711 10.1177/0022034515573964 PMC6728695

[20] Qin, A. et al. V-ATPases in osteoclasts: structure, function and potential inhibitors of bone resorption. *Int. J. Biochem. Cell Biol.* **44**, 1422–1435 (2012). 22652318 10.1016/j.biocel.2012.05.014

[21] Yang, D. et al. V-ATPase subunit ATP6AP1 (Ac45) regulates osteoclast differentiation, extracellular acidification, lysosomal trafficking, and protease exocytosis in osteoclast-mediated bone resorption. *J. Bone. Miner. Res.* **27**, 1695–1707 (2012). 22467241 10.1002/jbmr.1623 PMC3951719

[22] Chen, L. et al. Osteoclastic ATP6AP2 maintains beta-catenin levels to prevent hyper-osteoclastic activation and trabecular bone-loss. *J. Bone. Miner. Res.* **39**, 1821–1834 (2024). 39400061 10.1093/jbmr/zjae164

[23] Perdu, B. et al. Refined genomic localization of the genetic lesion in the osteopetrosis (op) rat and exclusion of three positional and functional candidate genes, Clcn7, Atp6v0c, and Slc9a3r2. *Calcif. Tissue Int.* **84**, 355–360 (2009). 19259722 10.1007/s00223-009-9229-7 PMC2718562

[24] Mattison, K. A. et al. ATP6V0C variants impair V-ATPase function causing a neurodevelopmental disorder often associated with epilepsy. *Brain* **146**, 1357–1372 (2023). 36074901 10.1093/brain/awac330 PMC10319782

[25] Kim, T. et al. ATP6v0d2 deficiency increases bone mass, but does not influence ovariectomy-induced bone loss. *Biochem. Biophys. Res. Commun.* **403**, 73–78 (2010). 21040703 10.1016/j.bbrc.2010.10.117 PMC3026595

[26] Guo, D. et al. Fluid shear stress changes cell morphology and regulates the expression of ATP6V1A and TCIRG1 mRNA in rat osteoclasts. *Mol. Med. Rep.* **3**, 173–178 (2010). 21472218 10.3892/mmr_00000236

[27] Lee, B. S., Holliday, L. S., Ojikutu, B., Krits, I. & Gluck, S. L. Osteoclasts express the B2 isoform of vacuolar H^+^-ATPase intracellularly and on their plasma membranes. *Am. J. Physiol.* **270**, C382–C388 (1996). 8772466 10.1152/ajpcell.1996.270.1.C382

[28] Bartkiewicz, M., Hernando, N., Reddy, S. V., Roodman, G. D. & Baron, R. Characterization of the osteoclast vacuolar H^+^-ATPase B-subunit. *Gene* **160**, 157–164 (1995). 7642089 10.1016/0378-1119(95)00228-x

[29] Chen, S. et al. Vacuolar H^+^-ATPase binding to microfilaments: regulation in response to phosphatidylinositol 3-kinase activity and detailed characterization of the actin-binding site in subunit B. *J. Biol. Chem.* **279**, 7988–7998 (2004). 14662773 10.1074/jbc.M305351200

[30] Feng, S. et al. Atp6v1c1 is an essential component of the osteoclast proton pump and in F-actin ring formation in osteoclasts. *Biochem. J.* **417**, 195–203 (2009). 18657050 10.1042/BJ20081073 PMC2773039

[31] McConnell, M. et al. Osteoclast proton pump regulator Atp6v1c1 enhances breast cancer growth by activating the mTORC1 pathway and bone metastasis by increasing V-ATPase activity. *Oncotarget* **8**, 47675–47690 (2017). 28504970 10.18632/oncotarget.17544 PMC5564597

[32] Tan, L. et al. Bivariate genome-wide association study implicates ATP6V1G1 as a novel pleiotropic locus underlying osteoporosis and age at menarche. *J. Clin. Endocrinol. Metab.* **100**, E1457–E1466 (2015). 26312577 10.1210/jc.2015-2095 PMC4702453

[33] Duan, X. et al. Deficiency of ATP6V1H causes bone loss by inhibiting bone resorption and bone formation through the TGF-beta1 pathway. *Theranostics* **6**, 2183–2195 (2016). 27924156 10.7150/thno.17140 PMC5135442

[34] Yang, S. et al. ATP6V1H deficiency impairs glucose tolerance by augmenting endoplasmic reticulum stress in high-fat diet-fed mice. *Arch. Biochem. Biophys.* **716**, 109116 (2022). 34990584 10.1016/j.abb.2022.109116

[35] Zhao, Z., Wang, X., Ma, Y. & Duan, X. Atp6v1h deficiency blocks bone loss in simulated microgravity mice through the Fos-Jun-Src-Integrin pathway. *Int. J. Mol. Sci*. **25**, 637 (2024). 10.3390/ijms25010637 PMC10779874 38203808

[36] Jonckheere, A. I., Smeitink, J. A. M. & Rodenburg, R. J. T. Mitochondrial ATP synthase: architecture, function and pathology. *J. Inherit. Metab. Dis.* **35**, 211–225 (2012). 21874297 10.1007/s10545-011-9382-9 PMC3278611

[37] Capaldi, R. A., Aggeler, R., Turina, P. & Wilkens, S. Coupling between catalytic sites and the proton channel in F1F0-type ATPases. *Trends Biochem.Sci.* **19**, 284–289 (1994). 8048168 10.1016/0968-0004(94)90006-x

[38] Xu, Y. et al. Targeted inhibition of ATP5B gene prevents bone erosion in collagen-induced arthritis by inhibiting osteoclastogenesis. *Pharmacol. Res.* **165**, 105458 (2021). 33515708 10.1016/j.phrs.2021.105458

[39] Griffiths, E. J. & Rutter, G. A. Mitochondrial calcium as a key regulator of mitochondrial ATP production in mammalian cells. *Biochim. Biophys. Acta* **1787**, 1324–1333 (2009). 19366607 10.1016/j.bbabio.2009.01.019

[40] Bagur, R. & Hajnoczky, G. Intracellular Ca^2+^ sensing: its role in calcium homeostasis and signaling. *Mol. Cell.* **66**, 780–788 (2017). 28622523 10.1016/j.molcel.2017.05.028 PMC5657234

[41] Di Leva, F., Domi, T., Fedrizzi, L., Lim, D. & Carafoli, E. The plasma membrane Ca^2+^ ATPase of animal cells: structure, function and regulation. *Arch. Biochem. Biophys.* **476**, 65–74 (2008). 18328800 10.1016/j.abb.2008.02.026

[42] Yang, Y. et al. Alteration of RANKL-induced osteoclastogenesis in primary cultured osteoclasts from SERCA2^+/-^ mice. *J. Bone. Miner. Res.* **24**, 1763–1769 (2009). 19419309 10.1359/jbmr.090420

[43] Wei, X. et al. Sigma-1 receptor attenuates osteoclastogenesis by promoting ER-associated degradation of SERCA2. *Embo Mol. Med.* **14**, e15373 (2022). 35611810 10.15252/emmm.202115373 PMC9260208

[44] Mentaverri, R., Kamel, S. & Brazier, M. Involvement of capacitive calcium entry and calcium store refilling in osteoclastic survival and bone resorption process. *Cell Calcium* **34**, 169–175 (2003). 12810059 10.1016/s0143-4160(03)00080-0

[45] Kim, H. J. et al. Plasma membrane calcium ATPase regulates bone mass by fine-tuning osteoclast differentiation and survival. *J. Cell. Biol.* **199**, 1145–1158 (2012). 23266958 10.1083/jcb.201204067 PMC3529522

[46] Go, W. & Korzh, V. Plasma membrane Ca^2+^ ATPase Atp2b1a regulates bone mineralization in zebrafish. *Bone* **54**, 48–57 (2013). 23353107 10.1016/j.bone.2013.01.026

[47] Perez-Canamas, A. et al. Sphingomyelin-induced inhibition of the plasma membrane calcium ATPase causes neurodegeneration in type A Niemann-Pick disease. *Mol. Psychiatry.* **22**, 711–723 (2017). 27620840 10.1038/mp.2016.148

[48] Oceandy, D., Buch, M. H., Cartwright, E. J. & Neyses, L. The emergence of plasma membrane calcium pump as a novel therapeutic target for heart disease. *Mini-Rev. Med. Chem* **6**, 583–588 (2006). 16719833 10.2174/138955706776876177

[49] Dong, X. et al. Hydroxyapatite nanoparticles induced calcium overload-initiated cancer cell-specific apoptosis through inhibition of PMCA and activation of calpain. *J. Mat. Chem. B.* **11**, 7609–7622 (2023). 10.1039/d3tb00542a 37403708

[50] Lubin, M. Intracellular potassium and macromolecular synthesis in mammalian cells. *Nature* **213**, 451–453 (1967). 6032224 10.1038/213451a0

[51] Fedosova, N. U., Habeck, M. & Nissen, P. Structure and Function of Na,K-ATPase-the sodium-potassium pump. *Compr. Physiol.* **12**, 2659–2679 (2021). 34964112 10.1002/cphy.c200018

[52] Baron, R., Neff, L., Roy, C., Boisvert, A. & Caplan, M. Evidence for a high and specific concentration of (Na^+^,K^+^)ATPase in the plasma membrane of the osteoclast. *Cell* **46**, 311–320 (1986). 2424614 10.1016/0092-8674(86)90748-8

[53] Xiong, S. et al. Immunization with Na^+^/K^+^ ATPase DR peptide prevents bone loss in an ovariectomized rat osteoporosis model. *Biochem. Pharmacol.* **156**, 281–290 (2018). 30134193 10.1016/j.bcp.2018.08.024

[54] Song, L., Li, X., Sun, Q. & Zhao, Y. Fxyd5 activates the NF‑kappaB pathway and is involved in chondrocytes inflammation and extracellular matrix degradation. *Mol. Med. Rep*. **25**, 134 (2022). 10.3892/mmr.2022.12650 PMC8908309 35191523

[55] de Tezanos Pinto, F. & Adamo, H. P. The strategic function of the P5-ATPase ATP13A2 in toxic waste disposal. *Neurochem. Int.* **112**, 108–113 (2018). 29169913 10.1016/j.neuint.2017.11.008

[56] Chen, P., Bornhorst, J. & Aschner, M. Manganese metabolism in humans. *Front. Biosci.* **23**, 1655–1679 (2018). 10.2741/4665 29293455

[57] Wells, E. M. et al. In vivo measurement of bone manganese and association with manual dexterity: a pilot study. *Environ. Res.* **160**, 35–38 (2018). 28961467 10.1016/j.envres.2017.09.016 PMC5962822

[58] Bae, Y. & Kim, M. Manganese supplementation improves mineral density of the spine and femur and serum osteocalcin in rats. *Biol. Trace Elem. Res.* **124**, 28–34 (2008). 18330520 10.1007/s12011-008-8119-6

[59] Luthen, F. et al. Influence of manganese ions on cellular behavior of human osteoblasts in vitro. *Biomol. Eng.* **24**, 531–536 (2007). 17884722 10.1016/j.bioeng.2007.08.003

[60] Barrioni, B. R. et al. Osteogenic potential of sol-gel bioactive glasses containing manganese. *J. Mater. Sci.-Mater. Med.* **30**, 86 (2019). 31302783 10.1007/s10856-019-6288-9

[61] Fujishiro, H. & Kambe, T. Manganese transport in mammals by zinc transporter family proteins, ZNT and ZIP. *J. Pharmacol. Sci.* **148**, 125–133 (2022). 34924116 10.1016/j.jphs.2021.10.011

[62] Zhang, R. et al. The endoplasmic reticulum ATP13A1 is essential for MAVS-mediated antiviral innate immunity. *Adv. Sci.* **9**, e2203831 (2022). 10.1002/advs.202203831 PMC9685455 36216581

[63] Kulicke, C. A. et al. The P5-type ATPase ATP13A1 modulates major histocompatibility complex I-related protein 1 (MR1)-mediated antigen presentation. *J. Biol. Chem.* **298**, 101542 (2022). 34968463 10.1016/j.jbc.2021.101542 PMC8808182

[64] Rentschler, G. et al. ATP13A2 (PARK9) polymorphisms influence the neurotoxic effects of manganese. *Neurotoxicology* **33**, 697–702 (2012). 22285144 10.1016/j.neuro.2012.01.007 PMC3997180

[65] Tan, J. et al. Regulation of intracellular manganese homeostasis by Kufor-Rakeb syndrome-associated ATP13A2 protein. *J. Biol. Chem.* **286**, 29654–29662 (2011). 21724849 10.1074/jbc.M111.233874 PMC3191006

[66] Daniel, G. et al. alpha-Synuclein-induced dopaminergic neurodegeneration in a rat model of Parkinson’s disease occurs independent of ATP13A2 (PARK9). *Neurobiol. Dis.* **73**, 229–243 (2015). 25461191 10.1016/j.nbd.2014.10.007

[67] Sakai, H. et al. Increases in intracellular pH facilitate endocytosis and decrease availability of voltage-gated proton channels in osteoclasts and microglia. *J. Physiol.* **591**, 5851–5866 (2013). 24081153 10.1113/jphysiol.2013.263558 PMC3872757

[68] Mori, H. et al. Regulatory mechanisms and physiological relevance of a voltage-gated H^+^ channel in murine osteoclasts: phorbol myristate acetate induces cell acidosis and the channel activation. *J. Bone. Miner. Res.* **18**, 2069–2076 (2003). 14606521 10.1359/jbmr.2003.18.11.2069

[69] Mori, H., Sakai, H., Morihata, H., Yamano, T. & Kuno, M. A voltage-gated H^+^ channel is a powerful mechanism for pH homeostasis in murine osteoclasts. *Kobe J. Med. Sci* **48**, 87–96 (2002). 12502906

[70] Bernheim, L. et al. A voltage-dependent proton current in cultured human skeletal muscle myotubes. *J. Physiol.* **470**, 313–333 (1993). 7508503 10.1113/jphysiol.1993.sp019860 PMC1143919

[71] Li, S. J., Zhao, Q., Zhou, Q. & Zhai, Y. Expression, purification, crystallization and preliminary crystallographic study of the carboxyl-terminal domain of the human voltage-gated proton channel Hv1. *Acta Crystallogr. Sect. F Struct. Biol. Cryst. Commun* **65**, 279–281 (2009). 19255483 10.1107/S1744309109003777 PMC2650464

[72] Capasso, M., DeCoursey, T. E. & Dyer, M. J. S. pH regulation and beyond: unanticipated functions for the voltage-gated proton channel, HVCN1. *Trends Cell Biol.* **21**, 20–28 (2011). 20961760 10.1016/j.tcb.2010.09.006 PMC3014425

[73] Asuaje, A. et al. The inhibition of voltage-gated H^+^ channel (HVCN1) induces acidification of leukemic Jurkat T cells promoting cell death by apoptosis. *Pflugers Arch.* **469**, 251–261 (2017). 28013412 10.1007/s00424-016-1928-0

[74] Carafoli, E. & Krebs, J. Why calcium? How calcium became the best communicator. *J. Biol. Chem.* **291**, 20849–20857 (2016). 27462077 10.1074/jbc.R116.735894 PMC5076498

[75] Berridge, M. J., Lipp, P. & Bootman, M. D. The versatility and universality of calcium signalling. *Nat. Rev. Mol. Cell Biol.* **1**, 11–21 (2000). 11413485 10.1038/35036035

[76] Okada, H., Okabe, K. & Tanaka, S. Finely-tuned calcium oscillations in osteoclast differentiation and bone resorption. *Int. J. Mol. Sci*. **22**, 180 (2020). 10.3390/ijms22010180 PMC7794828 33375370

[77] Catterall, W. A. Voltage-gated calcium channels. *Cold Spring Harbor Perspect. Biol.* **3**, a3947 (2011). 10.1101/cshperspect.a003947 PMC3140680 21746798

[78] Bennett, B. D., Alvarez, U. & Hruska, K. A. Receptor-operated osteoclast calcium sensing. *Endocrinology* **142**, 1968–1974 (2001). 11316762 10.1210/endo.142.5.8125

[79] Miyauchi, A. et al. Osteoclast cytosolic calcium, regulated by voltage-gated calcium channels and extracellular calcium, controls podosome assembly and bone resorption. *J. Cell. Biol.* **111**, 2543–2552 (1990). 1703539 10.1083/jcb.111.6.2543 PMC2116358

[80] Noh, A. L. S. M., Park, H., Zheng, T., Ha, H. & Yim, M. L-type Ca^2+^ channel agonist inhibits RANKL-induced osteoclast formation via NFATc1 down-regulation. *Life Sci.* **89**, 159–164 (2011). 21683712 10.1016/j.lfs.2011.05.009

[81] Fan, P. et al. Cav1.3 is upregulated in osteoporosis rat model and promotes osteoclast differentiation from preosteoclast cell line RAW264. *J. Cell. Physiol.* **234**, 12821–12827 (2019). 30741411 10.1002/jcp.27937

[82] Li, B. et al. Ionomycin ameliorates hypophosphatasia via rescuing alkaline phosphatase deficiency-mediated L-type Ca^2+^ channel internalization in mesenchymal stem cells. *Bone Res.* **8**, 19 (2020). 32351759 10.1038/s41413-020-0090-7 PMC7183511

[83] Datta, H. K., MacIntyre, I. & Zaidi, M. Intracellular calcium in the control of osteoclast function. I. Voltage-insensitivity and lack of effects of nifedipine, BAYK8644 and diltiazem. *Biochem. Biophys. Res. Commun.* **167**, 183–188 (1990). 1690001 10.1016/0006-291x(90)91748-h

[84] Striggow, F. & Ehrlich, B. E. Ligand-gated calcium channels inside and out. *Curr. Opin. Cell. Biol.* **8**, 490–495 (1996). 8791458 10.1016/s0955-0674(96)80025-1

[85] Rahman, T. et al. Two-pore channels provide insight into the evolution of voltage-gated Ca^2+^ and Na^+^ channels. *Sci. Signal.* **7**, ra109 (2014). 25406377 10.1126/scisignal.2005450 PMC4327855

[86] Berridge, M. J. Capacitative calcium entry. *Biochem. J.* **312**, 1–11 (1995). **Pt 1**. 7492298 10.1042/bj3120001 PMC1136219

[87] Inoue, R. TRP channels as a newly emerging non-voltage-gated Ca^2+^ entry channel superfamily. *Curr. Pharm. Des.* **11**, 1899–1914 (2005). 15974967 10.2174/1381612054021079

[88] Lee, H. C. & Aarhus, R. A derivative of NADP mobilizes calcium stores insensitive to inositol trisphosphate and cyclic ADP-ribose. *J. Biol. Chem.* **270**, 2152–2157 (1995). 7836444 10.1074/jbc.270.5.2152

[89] Calcraft, P. J. et al. NAADP mobilizes calcium from acidic organelles through two-pore channels. *Nature* **459**, 596–600 (2009). 19387438 10.1038/nature08030 PMC2761823

[90] Woll, K. A. & Van Petegem, F. Calcium-release channels: structure and function of IP(3) receptors and ryanodine receptors. *Physiol. Rev.* **102**, 209–268 (2022). 34280054 10.1152/physrev.00033.2020

[91] Morikawa, K., Goto, T., Tanimura, A., Kobayashi, S. & Maki, K. Distribution of inositol 1,4,5-trisphosphate receptors in rat osteoclasts. *Acta Histochem. Cytochem.* **41**, 7–13 (2008). 18493589 10.1267/ahc.07027 PMC2386513

[92] Yaroslavskiy, B. B., Turkova, I., Wang, Y., Robinson, L. J. & Blair, H. C. Functional osteoclast attachment requires inositol-1,4,5-trisphosphate receptor-associated cGMP-dependent kinase substrate. *Lab. Investig.* **90**, 1533–1542 (2010). 20567233 10.1038/labinvest.2010.120 PMC3114438

[93] Moonga, B. S. et al. Ca^2+^ influx through the osteoclastic plasma membrane ryanodine receptor. *Am. J. Physiol.-Renal Physiol.* **282**, F921–F932 (2002). 11934703 10.1152/ajprenal.00045.2000

[94] Ruas, M. et al. TPC1 has two variant isoforms, and their removal has different effects on endo-lysosomal functions compared to loss of TPC2. *Mol. Cell. Biol.* **34**, 3981–3992 (2014). 25135478 10.1128/MCB.00113-14 PMC4386455

[95] Wang, X. et al. TPC proteins are phosphoinositide-activated sodium-selective ion channels in endosomes and lysosomes. *Cell* **151**, 372–383 (2012). 23063126 10.1016/j.cell.2012.08.036 PMC3475186

[96] Notomi, T. et al. Role of lysosomal channel protein TPC2 in osteoclast differentiation and bone remodeling under normal and low-magnesium conditions. *J. Biol. Chem.* **292**, 20998–21010 (2017). 29084844 10.1074/jbc.M117.780072 PMC5743074

[97] Jha, A., Ahuja, M., Patel, S., Brailoiu, E. & Muallem, S. Convergent regulation of the lysosomal two-pore channel-2 by Mg^2+^, NAADP, PI(3,5)P(2) and multiple protein kinases. *EMBO J.* **33**, 501–511 (2014). 24502975 10.1002/embj.201387035 PMC3989630

[98] Prakriya, M. et al. Orai1 is an essential pore subunit of the CRAC channel. *Nature* **443**, 230–233 (2006). 16921383 10.1038/nature05122

[99] Hewavitharana, T., Deng, X., Soboloff, J. & Gill, D. L. Role of STIM and Orai proteins in the store-operated calcium signaling pathway. *Cell Calcium* **42**, 173–182 (2007). 17602740 10.1016/j.ceca.2007.03.009

[100] Zhou, Y. et al. The STIM-Orai coupling interface and gating of the Orai1 channel. *Cell Calcium* **63**, 8–13 (2017). 28087079 10.1016/j.ceca.2017.01.001 PMC5466457

[101] Robinson, L. J. et al. Gene disruption of the calcium channel Orai1 results in inhibition of osteoclast and osteoblast differentiation and impairs skeletal development. *Lab. Invest.* **92**, 1071–1083 (2012). 22546867 10.1038/labinvest.2012.72 PMC3387291

[102] Hwang, S. et al. Deletion of Orai1 alters expression of multiple genes during osteoclast and osteoblast maturation. *Cell Calcium* **52**, 488–500 (2012). 23122304 10.1016/j.ceca.2012.10.001 PMC3511630

[103] Hwang, S. & Putney, J. W. Orai1-mediated calcium entry plays a critical role in osteoclast differentiation and function by regulating activation of the transcription factor NFATc1. *FASEB J.* **26**, 1484–1492 (2012). 22198385 10.1096/fj.11-194399 PMC3316896

[104] Rychkov, G. Y. et al. Orai1- and Orai2-, but not Orai3-mediated I(CRAC) is regulated by intracellular pH. *J. Physiol.* **600**, 623–643 (2022). 34877682 10.1113/JP282502

[105] Inayama, M. et al. Orai1-Orai2 complex is involved in store-operated calcium entry in chondrocyte cell lines. *Cell Calcium* **57**, 337–347 (2015). 25769459 10.1016/j.ceca.2015.02.005

[106] Li, H. TRP channel classification. *Adv. Exp. Med. Biol.* **976**, 1–8 (2017). 28508308 10.1007/978-94-024-1088-4_1

[107] Ong, E. et al. A TRPC1 protein-dependent pathway regulates osteoclast formation and function. *J. Biol. Chem.* **288**, 22219–22232 (2013). 23770672 10.1074/jbc.M113.459826 PMC3829314

[108] Zhu, P. et al. TRPA1 aggravates osteoclastogenesis and osteoporosis through activating endoplasmic reticulum stress mediated by SRXN1. *Cell Death Dis.* **15**, 624 (2024). 39191723 10.1038/s41419-024-07018-5 PMC11349872

[109] Wen, W. et al. Expression and distribution of three transient receptor potential vanilloid(TRPV) channel proteins in human odontoblast-like cells. *J. Mol. Histol.* **48**, 367–377 (2017). 28905239 10.1007/s10735-017-9735-2

[110] Nishimura, H. et al. Transient receptor potential vanilloid 1 and 4 double knockout leads to increased bone mass in mice. *Bone Rep.* **12**, 100268 (2020). 32373678 10.1016/j.bonr.2020.100268 PMC7191598

[111] He, L. et al. TRPV1 deletion impaired fracture healing and inhibited osteoclast and osteoblast differentiation. *Sci. Rep.* **7**, 42385 (2017). 28225019 10.1038/srep42385 PMC5320507

[112] Kajiya, H. et al. RANKL-induced TRPV2 expression regulates osteoclastogenesis via calcium oscillations. *Cell Calcium* **48**, 260–269 (2010). 20980052 10.1016/j.ceca.2010.09.010

[113] Klein, S. et al. Modulation of transient receptor potential channels 3 and 6 regulates osteoclast function with impact on trabecular bone loss. *Calcif. Tissue Int.* **106**, 655–664 (2020). 32140760 10.1007/s00223-020-00673-8

[114] Jin, S. et al. Mechanical force modulates periodontal ligament stem cell characteristics during bone remodelling via TRPV4. *Cell Prolif.* **53**, e12912 (2020). 32964544 10.1111/cpr.12912 PMC7574874

[115] Pozo, A. et al. Cyclic adenosine monophosphate-dependent activation of transient receptor potential vanilloid 4 (TRPV4) channels in osteoblast-like MG-63 cells. *Cell. Signal.* **66**, 109486 (2020). 31778738 10.1016/j.cellsig.2019.109486

[116] Weber, K., Erben, R. G., Rump, A. & Adamski, J. Gene structure and regulation of the murine epithelial calcium channels ECaC1 and 2. *Biochem. Biophys. Res. Commun.* **289**, 1287–1294 (2001). 11741335 10.1006/bbrc.2001.6121

[117] Li, S. H. et al. TRPV5 and TRPV6 are expressed in placenta and bone tissues during pregnancy in mice. *Biotech. Histochem.* **94**, 244–251 (2019). 30916584 10.1080/10520295.2018.1548710

[118] van der Eerden, B. C. J. et al. The epithelial Ca^2+^ channel TRPV5 is essential for proper osteoclastic bone resorption. *Proc. Natl. Acad. Sci. USA* **102**, 17507–17512 (2005). 16291808 10.1073/pnas.0505789102 PMC1297662

[119] Luo, Z. et al. Alterations in the microenvironment and the effects produced of TRPV5 in osteoporosis. *J. Transl. Med.* **21**, 327 (2023). 37198647 10.1186/s12967-023-04182-8 PMC10190109

[120] Yelshanskaya, M. V., Nadezhdin, K. D., Kurnikova, M. G. & Sobolevsky, A. I. Structure and function of the calcium-selective TRP channel TRPV6. *J. Physiol.* **599**, 2673–2697 (2021). 32073143 10.1113/JP279024 PMC7689878

[121] Chen, F., Ni, B., Yang, Y. O., Ye, T. & Chen, A. Knockout of TRPV6 causes osteopenia in mice by increasing osteoclastic differentiation and activity. *Cell Physiol. Biochem.* **33**, 796–809 (2014). 24686448 10.1159/000358653

[122] Cho, C., Lee, Y., Kim, E., Hwang, E. M. & Park, J. Physiological functions of the TRPM4 channels via protein interactions. *BMB Rep.* **48**, 1–5 (2015). 25441424 10.5483/BMBRep.2015.48.1.252 PMC4345635

[123] Abed, E. & Moreau, R. Importance of melastatin-like transient receptor potential 7 and magnesium in the stimulation of osteoblast proliferation and migration by platelet-derived growth factor. *Am. J. Physiol. Cell Physiol.* **297**, C360–C368 (2009). 19474290 10.1152/ajpcell.00614.2008

[124] Shin, M. et al. Mesenchymal cell TRPM7 expression is required for bone formation via the regulation of chondrogenesis. *Bone* **166**, 116579 (2023). 36210025 10.1016/j.bone.2022.116579

[125] Treusch, S. et al. Caenorhabditis elegans functional orthologue of human protein h-mucolipin-1 is required for lysosome biogenesis. *Proc. Natl. Acad. Sci. USA* **101**, 4483–4488 (2004). 15070744 10.1073/pnas.0400709101 PMC384773

[126] Miedel, M. T. et al. Membrane traffic and turnover in TRP-ML1-deficient cells: a revised model for mucolipidosis type IV pathogenesis. *J. Exp. Med.* **205**, 1477–1490 (2008). 18504305 10.1084/jem.20072194 PMC2413042

[127] Park, H., Gholam-Zadeh, M., Suh, J. & Choi, H. Lycorine Attenuates Autophagy in Osteoclasts via an Axis of mROS/TRPML1/TFEB to Reduce LPS-Induced Bone Loss. *Oxidative Med. Cell. Longev.* **2019**, 8982147 (2019). 10.1155/2019/8982147 PMC6800915 31687088

[128] Erkhembaatar, M. et al. Lysosomal Ca^2+^ signaling is essential for osteoclastogenesis and bone remodeling. *J. Bone Miner. Res.* **32**, 385–396 (2017). 27589205 10.1002/jbmr.2986 PMC9850942

[129] Yamamoto, T. The molecular mechanisms of mitochondrial calcium uptake by calcium uniporter. *Yakugaku. Zasshi.* **141**, 491–499 (2021). 33790116 10.1248/yakushi.20-00204-1

[130] Williams, G. S. B., Boyman, L., Chikando, A. C., Khairallah, R. J. & Lederer, W. J. Mitochondrial calcium uptake. *Proc. Natl. Acad. Sci. USA* **110**, 10479–10486 (2013). 23759742 10.1073/pnas.1300410110 PMC3696793

[131] Hu, W., Yu, Y., Sun, Y., Yuan, F. & Zhao, F. MiR-25 overexpression inhibits titanium particle-induced osteoclast differentiation via down-regulation of mitochondrial calcium uniporter in vitro. *J. Orthop. Surg. Res.* **17**, 133 (2022). 35241114 10.1186/s13018-022-03030-7 PMC8895597

[132] Ward, K. M. & Wareham, A. C. Changes in membrane potential and potassium and sodium activities during postnatal development of mouse skeletal muscle. *Exp. Neurol.* **89**, 554–568 (1985). 4029335 10.1016/0014-4886(85)90006-8

[133] Kang, H. et al. Kcnn4 is a regulator of macrophage multinucleation in bone homeostasis and inflammatory disease. *Cell Rep* **8**, 1210–1224 (2014). 25131209 10.1016/j.celrep.2014.07.032 PMC4471813

[134] Yeon, J. et al. KCNK1 inhibits osteoclastogenesis by blocking the Ca2+ oscillation and JNK-NFATc1 signaling axis. *J. Cell Sci.* **128**, 3411–3419 (2015). 26208638 10.1242/jcs.170738

[135] Valverde, P., Kawai, T. & Taubman, M. A. Selective blockade of voltage-gated potassium channels reduces inflammatory bone resorption in experimental periodontal disease. *J. Bone. Miner. Res.* **19**, 155–164 (2004). 14753747 10.1359/JBMR.0301213

[136] Gonzalez, C. et al. K^+^ channels: function-structural overview. *Compr. Physiol.* **2**, 2087–2149 (2012). 23723034 10.1002/cphy.c110047

[137] Dudem, S., Sergeant, G. P., Thornbury, K. D. & Hollywood, M. A. Calcium-activated K^+^ channels (K(Ca)) and therapeutic implications. *Handb Exp Pharmacol* **267**, 379–416 (2021). 33945030 10.1007/164_2021_459

[138] Wilson, H. A. & Chused, T. M. Lymphocyte membrane potential and Ca^2+^-sensitive potassium channels described by oxonol dye fluorescence measurements. *J. Cell. Physiol.* **125**, 72–81 (1985). 2413058 10.1002/jcp.1041250110

[139] Brenner, R., Jegla, T. J., Wickenden, A., Liu, Y. & Aldrich, R. W. Cloning and functional characterization of novel large conductance calcium-activated potassium channel beta subunits, hKCNMB3 and hKCNMB4. *J. Biol. Chem.* **275**, 6453–6461 (2000). 10692449 10.1074/jbc.275.9.6453

[140] Ghatta, S., Nimmagadda, D., Xu, X. & O’Rourke, S. T. Large-conductance, calcium-activated potassium channels: structural and functional implications. *Pharmacol. Ther.* **110**, 103–116 (2006). 16356551 10.1016/j.pharmthera.2005.10.007

[141] Weatherall, K. L., Goodchild, S. J., Jane, D. E. & Marrion, N. V. Small conductance calcium-activated potassium channels: from structure to function. *Prog. Neurobiol.* **91**, 242–255 (2010). 20359520 10.1016/j.pneurobio.2010.03.002

[142] Jensen, B. S. et al. Characterization of the cloned human intermediate-conductance Ca^2+^-activated K^+^ channel. *Am J Physiol.* **275**, C848–C856 (1998). 9730970 10.1152/ajpcell.1998.275.3.C848

[143] Wu, S., Huang, Y. & Liao, Y. Effects of ibandronate sodium, a nitrogen-containing bisphosphonate, on intermediate-conductance calcium-activated potassium channels in osteoclast precursor cells (RAW 264.7). *J. Membr. Biol.* **248**, 103–115 (2015). 25362532 10.1007/s00232-014-9747-8

[144] Latorre, R. & Brauchi, S. Large conductance Ca^2+^-activated K^+^ (BK) channel: activation by Ca^2+^ and voltage. *Biol. Res.* **39**, 385–401 (2006). 17106573 10.4067/s0716-97602006000300003

[145] Jiang, L. et al. Activation of BK channels prevents diabetes-induced osteopenia by regulating mitochondrial Ca^2+^ and SLC25A5/ANT2-PINK1-PRKN-mediated mitophagy. *Autophagy* **20**, 2388–2404 (2024). 38873928 10.1080/15548627.2024.2367184 PMC11572260

[146] Quayle, J. M., Nelson, M. T. & Standen, N. B. ATP-sensitive and inwardly rectifying potassium channels in smooth muscle. *Physiol. Rev.* **77**, 1165–1232 (1997). 9354814 10.1152/physrev.1997.77.4.1165

[147] Kubo, Y., Baldwin, T. J., Jan, Y. N. & Jan, L. Y. Primary structure and functional expression of a mouse inward rectifier potassium channel. *Nature* **362**, 127–133 (1993). 7680768 10.1038/362127a0

[148] Hibino, H. et al. Inwardly rectifying potassium channels: their structure, function, and physiological roles. *Physiol. Rev.* **90**, 291–366 (2010). 20086079 10.1152/physrev.00021.2009

[149] Kelly, M. E., Dixon, S. J. & Sims, S. M. Inwardly rectifying potassium current in rabbit osteoclasts: a whole-cell and single-channel study. *J. Membr. Biol.* **126**, 171–181 (1992). 1593616 10.1007/BF00231915

[150] Kamuene, J. M., Xu, Y. & Plant, L. D. The pharmacology of two-pore domain potassium channels. *Handb. Exp. Pharmacol.* **267**, 417–443 (2021). 33880623 10.1007/164_2021_462

[151] Feliciangeli, S., Chatelain, F. C., Bichet, D. & Lesage, F. The family of K2P channels: salient structural and functional properties. *J. Physiol.* **593**, 2587–2603 (2015). 25530075 10.1113/jphysiol.2014.287268 PMC4500345

[152] Feinshreiber, L., Singer-Lahat, D., Ashery, U. & Lotan, I. Voltage-gated potassium channel as a facilitator of exocytosis. *Ann. N.Y. Acad. Sci.* **1152**, 87–92 (2009). 19161379 10.1111/j.1749-6632.2008.03997.x

[153] Gulbis, J. M., Mann, S. & MacKinnon, R. Structure of a voltage-dependent K^+^ channel beta subunit. *Cell* **97**, 943–952 (1999). 10399921 10.1016/s0092-8674(00)80805-3

[154] McCormack, K. et al. Genetic analysis of the mammalian K^+^ channel beta subunit Kvbeta 2 (Kcnab2). *J. Biol. Chem.* **277**, 13219–13228 (2002). 11825900 10.1074/jbc.M111465200

[155] Yan, L. et al. Fe2O3 nanoparticles suppress Kv1.3 channels via affecting the redox activity of Kvbeta2 subunit in Jurkat T cells. *Nanotechnology* **26**, 505103 (2015). 26584910 10.1088/0957-4484/26/50/505103

[156] Tu, H. et al. Hyperpolarization-activated, cyclic nucleotide-gated cation channels: roles in the differential electrophysiological properties of rat primary afferent neurons. *J. Neurosci. Res.* **76**, 713–722 (2004). 15139030 10.1002/jnr.20109

[157] Wilson, C. M., Stecyk, J. A. W., Couturier, C. S., Nilsson, G. E. & Farrell, A. P. Phylogeny and effects of anoxia on hyperpolarization-activated cyclic nucleotide-gated channel gene expression in the heart of a primitive chordate, the Pacific hagfish (Eptatretus stoutii). *J. Exp. Biol.* **216**, 4462–4472 (2013). 23997200 10.1242/jeb.094912

[158] Herrmann, S., Schnorr, S. & Ludwig, A. HCN channels-modulators of cardiac and neuronal excitability. *Int. J. Mol. Sci.* **16**, 1429–1447 (2015). 25580535 10.3390/ijms16011429 PMC4307311

[159] Notomi, T. et al. Zinc-induced effects on osteoclastogenesis involves activation of hyperpolarization-activated cyclic nucleotide modulated channels via changes in membrane potential. *J. Bone. Miner. Res.* **30**, 1618–1626 (2015). 25762086 10.1002/jbmr.2507

[160] Hiraoka, M. Metabolic pathways for ion homeostasis and persistent Na^+^ current. *J. Cardiovasc. Electrophysiol.* **17**, S124–S126 (2006). 16686666 10.1111/j.1540-8167.2006.00393.x

[161] Byers, M. R., Rafie, M. M. & Westenbroek, R. E. Dexamethasone effects on Na(v)1.6 in tooth pulp, dental nerves, and alveolar osteoclasts of adult rats. *Cell. Tissue. Res.* **338**, 217–226 (2009). 19763626 10.1007/s00441-009-0842-6

[162] Rolvien, T. et al. Severe bone loss and multiple fractures in SCN8A-related epileptic encephalopathy. *Bone* **103**, 136–143 (2017). 28676440 10.1016/j.bone.2017.06.025

[163] Hu, S. et al. Role of epithelial sodium channel in rat osteoclast differentiation and bone resorption. *Nan Fang Yi Ke Da Xue Xue Bao* **36**, 1148–1152 (2016). 27578589

[164] Dohke, T. et al. Regional osteoporosis due to osteoclast activation as a trigger for the pain-like behaviors in tail-suspended mice. *J. Orthop. Res.* **35**, 1226–1236 (2017). 27431941 10.1002/jor.23373

[165] Catarsi, S., Babinski, K. & Seguela, P. Selective modulation of heteromeric ASIC proton-gated channels by neuropeptide FF. *Neuropharmacology* **41**, 592–600 (2001). 11587714 10.1016/s0028-3908(01)00107-1

[166] Li, X. et al. Acid-sensing ion channel 1a is involved in acid-induced osteoclastogenesis by regulating activation of the transcription factor NFATc1. *FEBS Lett.* **587**, 3236–3242 (2013). 23994523 10.1016/j.febslet.2013.08.017

[167] Li, X., Ye, J., Xu, M., Zhao, M. & Yuan, F. Evidence that activation of ASIC1a by acidosis increases osteoclast migration and adhesion by modulating integrin/Pyk2/Src signaling pathway. *Osteoporos. Int.* **28**, 2221–2231 (2017). 28462470 10.1007/s00198-017-4017-0

[168] Baltaci, A. K. & Yuce, K. Zinc transporter proteins. *Neurochem. Res.* **43**, 517–530 (2018). 29243032 10.1007/s11064-017-2454-y

[169] McCall, K. A., Huang, C. & Fierke, C. A. Function and mechanism of zinc metalloenzymes. *J. Nutr.* **130**, 1437S–1446S (2000). 10801957 10.1093/jn/130.5.1437S

[170] Astudillo, C., Fernandez, A. C., Blair, M. W. & Cichy, K. A. The Phaseolus vulgaris ZIP gene family: identification, characterization, mapping, and gene expression. *Front. Plant Sci.* **4**, 286 (2013). 23908661 10.3389/fpls.2013.00286 PMC3726863

[171] Dufner-Beattie, J., Huang, Z. L., Geiser, J., Xu, W. & Andrews, G. K. Mouse ZIP1 and ZIP3 genes together are essential for adaptation to dietary zinc deficiency during pregnancy. *Genesis* **44**, 239–251 (2006). 16652366 10.1002/dvg.20211

[172] Franklin, R. B. et al. Human ZIP1 is a major zinc uptake transporter for the accumulation of zinc in prostate cells. *J. Inorg. Biochem.* **96**, 435–442 (2003). 12888280 10.1016/s0162-0134(03)00249-6 PMC4465841

[173] Eide, D. J. The SLC39 family of metal ion transporters. *Pflugers Arch.* **447**, 796–800 (2004). 12748861 10.1007/s00424-003-1074-3

[174] Schweigel-Rontgen, M. The families of zinc (SLC30 and SLC39) and copper (SLC31) transporters. *Curr. Top. Membr.* **73**, 321–355 (2014). 24745988 10.1016/B978-0-12-800223-0.00009-8

[175] Khadeer, M. A., Sahu, S. N., Bai, G., Abdulla, S. & Gupta, A. Expression of the zinc transporter ZIP1 in osteoclasts. *Bone* **37**, 296–304 (2005). 16005272 10.1016/j.bone.2005.04.035

[176] Shang, J. et al. Role of Zip1 in the regulation of NPY expression by MLT to promote fracture healing in rats. *Eur. J. Histochem*. **64**, 3183 (2020). 10.4081/ejh.2020.3183 PMC7731576 33334091

[177] Wang, F. et al. Zinc-stimulated endocytosis controls activity of the mouse ZIP1 and ZIP3 zinc uptake transporters. *J. Biol. Chem.* **279**, 24631–24639 (2004). 15054103 10.1074/jbc.M400680200

[178] Kagara, N., Tanaka, N., Noguchi, S. & Hirano, T. Zinc and its transporter ZIP10 are involved in invasive behavior of breast cancer cells. *Cancer Sci* **98**, 692–697 (2007). 17359283 10.1111/j.1349-7006.2007.00446.x PMC11159674

[179] Kelleher, S. L. et al. Mapping the zinc-transporting system in mammary cells: molecular analysis reveals a phenotype-dependent zinc-transporting network during lactation. *J. Cell. Physiol.* **227**, 1761–1770 (2012). 21702047 10.1002/jcp.22900 PMC3207005

[180] Chowanadisai, W. Comparative genomic analysis of slc39a12/ZIP12: insight into a zinc transporter required for vertebrate nervous system development. *PloS One* **9**, e111535 (2014). 25375179 10.1371/journal.pone.0111535 PMC4222902

[181] Bin, B. et al. Biochemical characterization of human ZIP13 protein: a homo-dimerized zinc transporter involved in the spondylocheiro dysplastic Ehlers-Danlos syndrome. *J. Biol. Chem.* **286**, 40255–40265 (2011). 21917916 10.1074/jbc.M111.256784 PMC3220551

[182] Fukada, T. et al. The zinc transporter SLC39A13/ZIP13 is required for connective tissue development; its involvement in BMP/TGF-beta signaling pathways. *PloS One* **3**, e3642 (2008). 18985159 10.1371/journal.pone.0003642 PMC2575416

[183] Thambiayya, K., Kaynar, A. M., St Croix, C. M. & Pitt, B. R. Functional role of intracellular labile zinc in pulmonary endothelium. *Pulm. Circ.* **2**, 443–451 (2012). 23372928 10.4103/2045-8932.105032 PMC3555414

[184] Hara, T. et al. Physiological roles of zinc transporters: molecular and genetic importance in zinc homeostasis. *J. Physiol. Sci.* **67**, 283–301 (2017). 28130681 10.1007/s12576-017-0521-4 PMC10717645

[185] Inoue, K. et al. Osteopenia and male-specific sudden cardiac death in mice lacking a zinc transporter gene, Znt5. *Hum. Mol. Genet.* **11**, 1775–1784 (2002). 12095919 10.1093/hmg/11.15.1775

[186] Lyubartseva, G., Smith, J. L., Markesbery, W. R. & Lovell, M. A. Alterations of zinc transporter proteins ZnT-1, ZnT-4 and ZnT-6 in preclinical Alzheimer’s disease brain. *Brain. Pathol.* **20**, 343–350 (2010). 19371353 10.1111/j.1750-3639.2009.00283.x PMC3175637

[187] Prakash, A., Bharti, K. & Majeed, A. B. A. Zinc: indications in brain disorders. *Fundam. Clin. Pharmacol.* **29**, 131–149 (2015). 25659970 10.1111/fcp.12110

[188] Zhang, X. et al. Zinc transporter 5 and zinc transporter 7 induced by high glucose protects peritoneal mesothelial cells from undergoing apoptosis. *Cell. Signal.* **25**, 999–1010 (2013). 23275032 10.1016/j.cellsig.2012.12.013

[189] Romani, A. M. P. Cellular magnesium homeostasis. *Arch. Biochem. Biophys.* **512**, 1–23 (2011). 21640700 10.1016/j.abb.2011.05.010 PMC3133480

[190] Romani, A. Regulation of magnesium homeostasis and transport in mammalian cells. *Arch. Biochem. Biophys.* **458**, 90–102 (2007). 16949548 10.1016/j.abb.2006.07.012

[191] Lopez Martinez, J., Sanchez Castilla, M., Garcia De Lorenzo, Y., Mateos, A. & Culebras Fernandez, J. M. Magnesium: metabolism and requirements. *Nutr. Hosp.* **12**, 4–14 (1997). 9147537

[192] Rubin, H. The membrane, magnesium, mitosis (MMM) model of cell proliferation control. *Magnes. Res.* **18**, 268–274 (2005). 16548142

[193] Jensen, P. E., Gibson, L. C. & Hunter, C. N. ATPase activity associated with the magnesium-protoporphyrin IX chelatase enzyme of Synechocystis PCC6803: evidence for ATP hydrolysis during Mg^2+^ insertion, and the MgATP-dependent interaction of the ChlI and ChlD subunits. *Biochem. J.* **339**, 127–134 (1999). **Pt 1**. 10085236 PMC1220136

[194] Garfinkel, L. & Garfinkel, D. Magnesium regulation of the glycolytic pathway and the enzymes involved. *Magnesium* **4**, 60–72 (1985). 2931560

[195] Leidi, M., Dellera, F., Mariotti, M. & Maier, J. A. M. High magnesium inhibits human osteoblast differentiation in vitro. *Magnes. Res.* **24**, 1–6 (2011). 21421455 10.1684/mrh.2011.0271

[196] Castiglioni, S., Cazzaniga, A., Albisetti, W. & Maier, J. A. M. Magnesium and osteoporosis: current state of knowledge and future research directions. *Nutrients* **5**, 3022–3033 (2013). 23912329 10.3390/nu5083022 PMC3775240

[197] Qiao, W. et al. TRPM7 kinase-mediated immunomodulation in macrophage plays a central role in magnesium ion-induced bone regeneration. *Nat. Commun.* **12**, 2885 (2021). 34001887 10.1038/s41467-021-23005-2 PMC8128914

[198] Astor, M. C. et al. Hypomagnesemia and functional hypoparathyroidism due to novel mutations in the Mg-channel TRPM6. *Endocr. Connect.* **4**, 215–222 (2015). 26273099 10.1530/EC-15-0066 PMC4566842

[199] Risco, F. & Traba, M. L. Bone-specific binding sites for 1,25(OH)2D3 in magnesium deficiency. *J. Physiol. Biochem.* **60**, 199–203 (2004). 15700766 10.1007/BF03167029

[200] Wolf, F. I. & Trapani, V. MagT1: a highly specific magnesium channel with important roles beyond cellular magnesium homeostasis. *Magnes. Res.* **24**, S86–S91 (2011). 21947671 10.1684/mrh.2011.0288

[201] Castiglioni, S., Romeo, V., Locatelli, L., Cazzaniga, A. & Maier, J. A. M. TRPM7 and MagT1 in the osteogenic differentiation of human mesenchymal stem cells in vitro. *Sci. Rep.* **8**, 16195 (2018). 30385806 10.1038/s41598-018-34324-8 PMC6212439

[202] Gregan, J., Kolisek, M. & Schweyen, R. J. Mitochondrial Mg^2+^ homeostasis is critical for group II intron splicing in vivo. *Genes. Dev.* **15**, 2229–2237 (2001). 11544180 10.1101/gad.201301 PMC312778

[203] Cabezas-Bratesco, D. et al. The different roles of the channel-kinases TRPM6 and TRPM7. *Curr. Med. Chem.* **22**, 2943–2953 (2015). 26179995 10.2174/0929867322666150716115644

[204] Yu, N. et al. SLC41A1 knockdown inhibits angiotensin II-induced cardiac fibrosis by preventing Mg^2+^ efflux and Ca^2+^ signaling in cardiac fibroblasts. *Arch. Biochem. Biophys.* **564**, 74–82 (2014). 25263961 10.1016/j.abb.2014.09.013

[205] de Baaij, J. H. F. et al. Identification of SLC41A3 as a novel player in magnesium homeostasis. *Sci. Rep.* **6**, 28565 (2016). 27349617 10.1038/srep28565 PMC4923877

[206] Klimaczewski, C. V. et al. Peumus boldus attenuates copper-induced toxicity in Drosophila melanogaster. *Biomed. Pharmacother.* **97**, 1–8 (2018). 29080449 10.1016/j.biopha.2017.09.130

[207] Li, D., Gao, Z., Li, Q., Liu, X. & Liu, H. Cuproptosis-a potential target for the treatment of osteoporosis. *Front. Endocrinol.* **14**, 1135181 (2023). 10.3389/fendo.2023.1135181 PMC10196240 37214253

[208] Xu, X. et al. Copper-modified Ti6Al4 V suppresses inflammatory response and osteoclastogenesis while enhancing extracellular matrix formation for osteoporotic bone regeneration. *ACS Biomater. Sci. Eng.* **4**, 3364–3373 (2018). 33435071 10.1021/acsbiomaterials.8b00736

[209] Ohrvik, H. & Thiele, D. J. How copper traverses cellular membranes through the mammalian copper transporter 1, Ctr1. *Ann. N.Y. Acad. Sci.* **1314**, 32–41 (2014). 24697869 10.1111/nyas.12371 PMC4158275

[210] Leary, S. C. & Ralle, M. Advances in visualization of copper in mammalian systems using X-ray fluorescence microscopy. *Curr. Opin. Chem. Biol.* **55**, 19–25 (2020). 31911338 10.1016/j.cbpa.2019.12.002 PMC7237281

[211] Lutsenko, S. Dynamic and cell-specific transport networks for intracellular copper ions. *J. Cell Sci*. **134**, jcs240523 (2021). 10.1242/jcs.240523 PMC8627558 34734631

[212] Ponka, P., Beaumont, C. & Richardson, D. R. Function and regulation of transferrin and ferritin. *Semin. Hematol.* **35**, 35–54 (1998). 9460808

[213] Knutson, M. D. Non-transferrin-bound iron transporters. *Free. Radic. Biol. Med.* **133**, 101–111 (2019). 30316781 10.1016/j.freeradbiomed.2018.10.413

[214] Hentze, M. W., Muckenthaler, M. U., Galy, B. & Camaschella, C. Two to tango: regulation of mammalian iron metabolism. *Cell* **142**, 24–38 (2010). 20603012 10.1016/j.cell.2010.06.028

[215] Balogh, E., Paragh, G. & Jeney, V. Influence of iron on bone homeostasis. *Pharmaceuticals.* **11**, 107 (2018). 10.3390/ph11040107 PMC6316285 30340370

[216] Dong, Y. et al. A clinical-stage Nrf2 activator suppresses osteoclast differentiation via the iron-ornithine axis. *Cell Metab* **36**, 1679–1695 (2024). 38569557 10.1016/j.cmet.2024.03.005

[217] Donovan, A. et al. Positional cloning of zebrafish ferroportin1 identifies a conserved vertebrate iron exporter. *Nature* **403**, 776–781 (2000). 10693807 10.1038/35001596

[218] Xie, W., Lorenz, S., Dolder, S. & Hofstetter, W. Extracellular iron is a modulator of the differentiation of osteoclast lineage cells. *Calcif. Tissue Int.* **98**, 275–283 (2016). 26615413 10.1007/s00223-015-0087-1

[219] Lin, F. et al. Mesenchymal stem cells protect against ferroptosis via exosome-mediated stabilization of SLC7A11 in acute liver injury. *Cell Death Dis* **13**, 271 (2022). 35347117 10.1038/s41419-022-04708-w PMC8960810

[220] Sun, F., Zhou, J. L., Liu, Z. L., Jiang, Z. W. & Peng, H. Dexamethasone induces ferroptosis via P53/SLC7A11/GPX4 pathway in glucocorticoid-induced osteonecrosis of the femoral head. *Biochem. Biophys. Res. Commun.* **602**, 149–155 (2022). 35276555 10.1016/j.bbrc.2022.02.112

[221] Yang, Y. et al. Targeting ferroptosis suppresses osteocyte glucolipotoxicity and alleviates diabetic osteoporosis. *Bone Res.* **10**, 26 (2022). 35260560 10.1038/s41413-022-00198-w PMC8904790

[222] Ge, W. et al. Advanced glycation end products promote osteoporosis by inducing ferroptosis in osteoblasts. *Mol. Med. Rep*. **25**, 140 (2022). 10.3892/mmr.2022.12656 PMC8908347 35211757

[223] Ma, H. et al. Melatonin suppresses ferroptosis induced by high glucose via activation of the Nrf2/HO-1 signaling pathway in type 2 diabetic osteoporosis. *Oxidative Med. Cell. Longev.* **2020**, 9067610 (2020). 10.1155/2020/9067610 PMC7732386 33343809

[224] Wang, X. et al. Mitochondrial ferritin deficiency promotes osteoblastic ferroptosis via mitophagy in type 2 diabetic osteoporosis. *Biol. Trace Elem. Res.* **200**, 298–307 (2022). 33594527 10.1007/s12011-021-02627-z

[225] Qu, X., Sun, Z., Wang, Y. & Ong, H. S. Zoledronic acid promotes osteoclasts ferroptosis by inhibiting FBXO9-mediated p53 ubiquitination and degradation. *Peerj* **9**, e12510 (2021). 35003915 10.7717/peerj.12510 PMC8684721

[226] Ni, S. et al. Hypoxia inhibits RANKL-induced ferritinophagy and protects osteoclasts from ferroptosis. *Free. Radic. Biol. Med.* **169**, 271–282 (2021). 33895289 10.1016/j.freeradbiomed.2021.04.027

[227] Coste, B. et al. Piezo1 and Piezo2 are essential components of distinct mechanically activated cation channels. *Science* **330**, 55–60 (2010). 20813920 10.1126/science.1193270 PMC3062430

[228] Wang, L. et al. Mechanical sensing protein PIEZO1 regulates bone homeostasis via osteoblast-osteoclast crosstalk. *Nat. Commun.* **11**, 282 (2020). 31941964 10.1038/s41467-019-14146-6 PMC6962448

[229] Hendrickx, G. et al. Piezo1 inactivation in chondrocytes impairs trabecular bone formation. *J. Bone. Miner. Res.* **36**, 369–384 (2021). 33180356 10.1002/jbmr.4198

[230] Bratengeier, C., Liszka, A., Hoffman, J., Bakker, A. D. & Fahlgren, A. High shear stress amplitude in combination with prolonged stimulus duration determine induction of osteoclast formation by hematopoietic progenitor cells. *FASEB J.* **34**, 3755–3772 (2020). 31957079 10.1096/fj.201901458R

[231] Shindo, S. et al. Piezo1 protects against inflammatory bone loss via a unique Ca(2+)-independent mechanism in osteoclasts. *Front. Immunol.* **16**, 1661538 (2025). 10.3389/fimmu.2025.1661538 PMC12507952 41080556

[232] Guan, H. et al. Magnetic aggregation-induced bone-targeting nanocarrier with effects of Piezo1 activation and osteogenic-angiogenic coupling for osteoporotic bone repair. *Adv. Mater.* **36**, e2312081 (2024). 38102981 10.1002/adma.202312081

[233] Cai, G. et al. Piezo1-mediated M2 macrophage mechanotransduction enhances bone formation through secretion and activation of transforming growth factor-beta1. *Cell Prolif.* **56**, e13440 (2023). 36880296 10.1111/cpr.13440 PMC10472522

[234] Wang, S. et al. Nociceptor neurons facilitate orthodontic tooth movement via Piezo2 in mice. *J. Dent. Res*. **104**, 890-899 (2025). 10.1177/00220345251317429 PMC12319647 40071303

[235] Wyllie, D. J. A. & Bowie, D. Ionotropic glutamate receptors: structure, function and dysfunction. *J. Physiol.* **600**, 175–179 (2022). 35028955 10.1113/JP282389

[236] Kelly, J. B. & Zhang, H. Contribution of AMPA and NMDA receptors to excitatory responses in the inferior colliculus. *Hear. Res.* **168**, 35–42 (2002). 12117507 10.1016/s0378-5955(02)00372-6

[237] Merle, B., Itzstein, C., Delmas, P. D. & Chenu, C. NMDA glutamate receptors are expressed by osteoclast precursors and involved in the regulation of osteoclastogenesis. *J. Cell. Biochem.* **90**, 424–436 (2003). 14505357 10.1002/jcb.10625

[238] Kiyohara, S. et al. Effects of N-methyl-d-aspartate receptor antagonist MK-801 (dizocilpine) on bone homeostasis in mice. *J. Oral Biosci.* **62**, 131–138 (2020). 32289529 10.1016/j.job.2020.03.003

[239] Baek, J. M., Kim, J., Yoon, K., Oh, J. & Lee, M. S. Ebselen is a potential anti-osteoporosis agent by suppressing receptor activator of nuclear factor kappa-B ligand-induced osteoclast differentiation in vitro and lipopolysaccharide-induced inflammatory bone destruction in vivo. *Int. J. Biol. Sci.* **12**, 478–488 (2016). 27019631 10.7150/ijbs.13815 PMC4807414

[240] Coddou, C., Yan, Z., Obsil, T., Huidobro-Toro, J. P. & Stojilkovic, S. S. Activation and regulation of purinergic P2X receptor channels. *Pharmacol. Rev.* **63**, 641–683 (2011). 21737531 10.1124/pr.110.003129 PMC3141880

[241] Schmid, R. & Evans, R. J. ATP-gated P2X receptor channels: molecular insights into functional roles. *Annu. Rev. Physiol.* **81**, 43–62 (2019). 30354932 10.1146/annurev-physiol-020518-114259

[242] Barbosa, C. M. V. et al. Differentiation of hematopoietic stem cell and myeloid populations by ATP is modulated by cytokines. *Cell Death Dis.* **2**, e165 (2011). 21633388 10.1038/cddis.2011.49 PMC3168991

[243] Naemsch, L. N., Weidema, A. F., Sims, S. M., Underhill, T. M. & Dixon, S. J. P2X(4) purinoceptors mediate an ATP-activated, non-selective cation current in rabbit osteoclasts. *J. Cell Sci.* **112**, 4425–4435 (1999). **Pt 23**. 10564660 10.1242/jcs.112.23.4425

[244] Kim, H. et al. The purinergic receptor P2X5 regulates inflammasome activity and hyper-multinucleation of murine osteoclasts. *Sci. Rep.* **7**, 196 (2017). 28298636 10.1038/s41598-017-00139-2 PMC5427844

[245] Kim, H. et al. The purinergic receptor P2X5 contributes to bone loss in experimental periodontitis. *BMB Rep.* **51**, 468–473 (2018). 30103845 10.5483/BMBRep.2018.51.9.126 PMC6177510

[246] Agrawal, A. & Jorgensen, N. R. Extracellular purines and bone homeostasis. *Biochem. Pharmacol.* **187**, 114425 (2021). 33482152 10.1016/j.bcp.2021.114425

[247] Morrison, M. S., Turin, L., King, B. F., Burnstock, G. & Arnett, T. R. ATP is a potent stimulator of the activation and formation of rodent osteoclasts. *J. Physiol.* **511**, 495–500 (1998). **Pt 2**. 9706025 10.1111/j.1469-7793.1998.495bh.x PMC2231120

[248] Surprenant, A., Rassendren, F., Kawashima, E., North, R. A. & Buell, G. The cytolytic P2Z receptor for extracellular ATP identified as a P2X receptor (P2X7). *Science* **272**, 735–738 (1996). 8614837 10.1126/science.272.5262.735

[249] Brandao-Burch, A., Key, M. L., Patel, J. J., Arnett, T. R. & Orriss, I. R. The P2X7 receptor is an important regulator of extracellular ATP levels. *Front. Endocrinol.* **3**, 41 (2012). 10.3389/fendo.2012.00041 PMC3355863 22654865

[250] Lu, J. et al. New mechanistic understanding of osteoclast differentiation and bone resorption mediated by P2X7 receptors and PI3K-Akt-GSK3beta signaling. *Cell. Mol. Biol. Lett.* **29**, 100 (2024). 38977961 10.1186/s11658-024-00614-5 PMC11232284

[251] Agrawal, A. et al. The effects of P2X7 receptor antagonists on the formation and function of human osteoclasts in vitro. *Purinergic Signal* **6**, 307–315 (2010). 21103214 10.1007/s11302-010-9181-z PMC2947658

[252] Gartland, A., Buckley, K. A., Bowler, W. B. & Gallagher, J. A. Blockade of the pore-forming P2X7 receptor inhibits formation of multinucleated human osteoclasts in vitro. *Calcif. Tissue Int.* **73**, 361–369 (2003). 12874700 10.1007/s00223-002-2098-y

[253] Armstrong, S., Pereverzev, A., Dixon, S. J. & Sims, S. M. Activation of P2X7 receptors causes isoform-specific translocation of protein kinase C in osteoclasts. *J. Cell Sci.* **122**, 136–144 (2009). 19066285 10.1242/jcs.031534

[254] Korcok, J., Raimundo, L. N., Ke, H. Z., Sims, S. M. & Dixon, S. J. Extracellular nucleotides act through P2X7 receptors to activate NF-kappaB in osteoclasts. *J. Bone. Miner. Res.* **19**, 642–651 (2004). 15005852 10.1359/JBMR.040108

[255] Ma, Y. et al. The effect of P2X7R-mediated Ca^2+^ signaling in OPG-induced osteoclasts adhesive structure damage. *Exp. Cell Res.* **383**, 111555 (2019). 31415763 10.1016/j.yexcr.2019.111555

[256] Hiken, J. F. & Steinberg, T. H. ATP downregulates P2X7 and inhibits osteoclast formation in RAW cells. *Am. J. Physiol. Cell Physiol.* **287**, C403–C412 (2004). 15070812 10.1152/ajpcell.00361.2003

[257] Ma, Y. et al. P2X7 receptor knockdown suppresses osteoclast differentiation by inhibiting autophagy and Ca^2+^/calcineurin signaling. *Mol. Med. Rep*. **25**, 160 (2022). 10.3892/mmr.2022.12677 PMC8941524 35266012

[258] Braun, A. P. & Schulman, H. Distinct voltage-dependent gating behaviours of a swelling-activated chloride current in human epithelial cells. *J. Physiol.* **495**, 743–753 (1996). **Pt 3**. 8887780 10.1113/jphysiol.1996.sp021630 PMC1160779

[259] Berndt, N., Hoffmann, S., Benda, J. & Holzhutter, H. The influence of the chloride currents on action potential firing and volume regulation of excitable cells studied by a kinetic model. *J. Theor. Biol.* **276**, 42–49 (2011). 21295041 10.1016/j.jtbi.2011.01.022

[260] Gelband, C. H., Greco, P. G. & Martens, J. R. Voltage-dependent chloride channels: invertebrates to man. *J. Exp. Zool.* **275**, 277–282 (1996). 8759924 10.1002/(SICI)1097-010X(19960701)275:4<277::AID-JEZ5>3.0.CO;2-M

[261] Nilius, B. & Droogmans, G. Amazing chloride channels: an overview. *Acta Physiol. Scand.* **177**, 119–147 (2003). 12558550 10.1046/j.1365-201X.2003.01060.x

[262] Jentsch, T. J., Neagoe, I. & Scheel, O. CLC chloride channels and transporters. *Curr. Opin. Neurobiol.* **15**, 319–325 (2005). 15913981 10.1016/j.conb.2005.05.002

[263] Pangrazio, A. et al. Molecular and clinical heterogeneity in CLCN7-dependent osteopetrosis: report of 20 novel mutations. *Hum. Mutat.* **31**, E1071–E1080 (2010). 19953639 10.1002/humu.21167

[264] Kornak, U. et al. Loss of the ClC-7 chloride channel leads to osteopetrosis in mice and man. *Cell* **104**, 205–215 (2001). 11207362 10.1016/s0092-8674(01)00206-9

[265] Piret, S. E. et al. Autosomal dominant osteopetrosis associated with renal tubular acidosis is due to a CLCN7 mutation. *Am. J. Med. Genet. A* **170**, 2988–2992 (2016). 27540713 10.1002/ajmg.a.37755 PMC5132132

[266] Majumdar, A., Capetillo-Zarate, E., Cruz, D., Gouras, G. K. & Maxfield, F. R. Degradation of Alzheimer’s amyloid fibrils by microglia requires delivery of ClC-7 to lysosomes. *Mol. Biol. Cell.* **22**, 1664–1676 (2011). 21441306 10.1091/mbc.E10-09-0745 PMC3093319

[267] Neutzsky-Wulff, A. V., Karsdal, M. A. & Henriksen, K. Characterization of the bone phenotype in ClC-7-deficient mice. *Calcif. Tissue Int.* **83**, 425–437 (2008). 18958510 10.1007/s00223-008-9185-7

[268] Lange, P. F., Wartosch, L., Jentsch, T. J. & Fuhrmann, J. C. ClC-7 requires Ostm1 as a beta-subunit to support bone resorption and lysosomal function. *Nature* **440**, 220–223 (2006). 16525474 10.1038/nature04535

[269] Di Zanni, E. et al. Pathobiologic mechanisms of neurodegeneration in osteopetrosis derived from structural and functional analysis of 14 ClC-7 mutants. *J. Bone. Miner. Res.* **36**, 531–545 (2021). 33125761 10.1002/jbmr.4200

[270] Parenti, G., Andria, G. & Valenzano, K. J. Pharmacological chaperone therapy: preclinical development, clinical translation, and prospects for the treatment of lysosomal storage disorders. *Mol. Ther.* **23**, 1138–1148 (2015). 25881001 10.1038/mt.2015.62 PMC4817787

[271] Wu, J. Z. et al. ClC-7 drives intraphagosomal chloride accumulation to support hydrolase activity and phagosome resolution. *J. Cell. Biol*. **222**, e202208155 (2023). 10.1083/jcb.202208155 PMC10072274 37010469

[272] Iyer, H. & Talbot, W. S. The Cl- transporter ClC-7 is essential for phagocytic clearance by microglia. *J. Cell Sci*. **137**, jcs261616 (2024). 10.1242/jcs.261616 PMC10911276 38294065

[273] Cai, Y. et al. Downregulation of chloride voltage-gated channel 7 contributes to hyperalgesia following spared nerve injury. *J. Biol. Chem.* **300**, 107779 (2024). 39276933 10.1016/j.jbc.2024.107779 PMC11490881

[274] Wang, H. et al. ClC-7 Deficiency impairs tooth development and eruption. *Sci. Rep.* **6**, 19971 (2016). 26829236 10.1038/srep19971 PMC4734291

[275] Zhang, Y. et al. ClC-7 Regulates the pattern and early development of craniofacial bone and tooth. *Theranostics* **9**, 1387–1400 (2019). 30867839 10.7150/thno.29761 PMC6401512

[276] Ohgi, K., Okamoto, F., Kajiya, H., Sakagami, R. & Okabe, K. Antibodies against ClC7 inhibit extracellular acidification-induced Cl^-^ currents and bone resorption activity in mouse osteoclasts. *Naunyn. Schmiedebergs. Arch. Pharmacol.* **383**, 79–90 (2011). 21061117 10.1007/s00210-010-0576-8

[277] Okamoto, F. et al. Intracellular ClC-3 chloride channels promote bone resorption in vitro through organelle acidification in mouse osteoclasts. *Am. J. Physiol. Cell Physiol.* **294**, C693–C701 (2008). 18234851 10.1152/ajpcell.00251.2007

[278] Wang, H. et al. Chloride channel ClC-3 promotion of osteogenic differentiation through Runx2. *J. Cell. Biochem.* **111**, 49–58 (2010). 20506205 10.1002/jcb.22658

[279] Wang, H. et al. ClC-3 chloride channel functions as a mechanically sensitive channel in osteoblasts. *Biochem. Cell. Biol.* **93**, 558–565 (2015). 26436462 10.1139/bcb-2015-0018

[280] Al Khamici, H. et al. Members of the chloride intracellular ion channel protein family demonstrate glutaredoxin-like enzymatic activity. *PloS One* **10**, e115699 (2015). 25581026 10.1371/journal.pone.0115699 PMC4291220

[281] Singh, H. Two decades with dimorphic chloride intracellular channels (CLICs). *FEBS Lett.* **584**, 2112–2121 (2010). 20226783 10.1016/j.febslet.2010.03.013

[282] Sun, H. J. et al. A proteomic analysis during serial subculture and osteogenic differentiation of human mesenchymal stem cell. *J. Orthop. Res.* **24**, 2059–2071 (2006). 16947300 10.1002/jor.20273

[283] Jiang, L. et al. Intracellular chloride channel protein CLIC1 regulates macrophage function through modulation of phagosomal acidification. *J. Cell Sci.* **125**, 5479–5488 (2012). 22956539 10.1242/jcs.110072 PMC3561857

[284] Schaller, S. et al. The chloride channel inhibitor NS3736 [corrected] prevents bone resorption in ovariectomized rats without changing bone formation. *J. Bone. Miner. Res.* **19**, 1144–1153 (2004). 15176998 10.1359/JBMR.040302

[285] Proutski, I., Karoulias, N. & Ashley, R. H. Overexpressed chloride intracellular channel protein CLIC4 (p64H1) is an essential component of novel plasma membrane anion channels. *Biochem. Biophys. Res. Commun.* **297**, 317–322 (2002). 12237120 10.1016/s0006-291x(02)02199-x

[286] Singh, H. & Ashley, R. H. CLIC4 (p64H1) and its putative transmembrane domain form poorly selective, redox-regulated ion channels. *Mol. Membr. Biol.* **24**, 41–52 (2007). 17453412 10.1080/09687860600927907

[287] Argenzio, E. et al. CLIC4 regulates cell adhesion and beta1 integrin trafficking. *J. Cell Sci.* **127**, 5189–5203 (2014). 25344254 10.1242/jcs.150623

[288] Domingo-Fernandez, R., Coll, R. C., Kearney, J., Breit, S. & O’Neill, L. A. J. The intracellular chloride channel proteins CLIC1 and CLIC4 induce IL-1beta transcription and activate the NLRP3 inflammasome. *J. Biol. Chem.* **292**, 12077–12087 (2017). 28576828 10.1074/jbc.M117.797126 PMC5519359

[289] Abdul-Salam, V. B. et al. CLIC4/Arf6 pathway. *Circ. Res.* **124**, 52–65 (2019). 30582444 10.1161/CIRCRESAHA.118.313705 PMC6325770

[290] Hartzell, H. C., Yu, K., Xiao, Q., Chien, L. & Qu, Z. Anoctamin/TMEM16 family members are Ca^2+^-activated Cl- channels. *J. Physiol.* **587**, 2127–2139 (2009). 19015192 10.1113/jphysiol.2008.163709 PMC2697287

[291] Kamaleddin, M. A. Molecular, biophysical, and pharmacological properties of calcium-activated chloride channels. *J. Cell. Physiol.* **233**, 787–798 (2018). 28121009 10.1002/jcp.25823

[292] Ehlen, H. W. A. et al. Inactivation of anoctamin-6/Tmem16f, a regulator of phosphatidylserine scrambling in osteoblasts, leads to decreased mineral deposition in skeletal tissues. *J. Bone. Miner. Res.* **28**, 246–259 (2013). 22936354 10.1002/jbmr.1751

[293] Oh, U. & Jung, J. Cellular functions of TMEM16/anoctamin. *Pflugers Arch.* **468**, 443–453 (2016). 26811235 10.1007/s00424-016-1790-0 PMC4751194

[294] Ousingsawat, J. et al. Anoctamin 6 mediates effects essential for innate immunity downstream of P2X7 receptors in macrophages. *Nat. Commun.* **6**, 6245 (2015). 25651887 10.1038/ncomms7245

[295] Liu, G. et al. Involvement of Ca^2+^ activated Cl- channel Ano6 in platelet activation and apoptosis. *Cell Physiol. Biochem.* **37**, 1934–1944 (2015). 26584292 10.1159/000438554

[296] Shaibani, A., Khan, S. & Shinawi, M. Autosomal dominant ANO5-related disorder associated with myopathy and gnathodiaphyseal dysplasia. *Neurol. Genet.* **7**, e612 (2021). 34291158 10.1212/NXG.0000000000000612 PMC8290902

[297] Liu, X. et al. Ano5 deficiency disturbed bone formation by inducing osteoclast apoptosis in Gnathodiaphyseal dysplasia. *Exp. Cell Res*. **447**, 114493 (2025). 10.1016/j.yexcr.2025.114493 40049314

[298] Zhang, S. et al. Anoctamin5 deficiency enhances ATG9A-dependent autophagy, inducing osteogenesis and gnathodiaphyseal dysplasia-like bone formation. *JCI Insight.* **10**, e189817 (2025). 10.1172/jci.insight.189817 PMC12016930 40067389

[299] Ishihara, K., Suzuki, J. & Nagata, S. Role of Ca^2+^ in the stability and function of TMEM16F and 16K. *Biochemistry* **55**, 3180–3188 (2016). 27227820 10.1021/acs.biochem.6b00176

[300] Jacobsen, K. S. et al. The role of TMEM16A (ANO1) and TMEM16F (ANO6) in cell migration. *Pflugers Arch.* **465**, 1753–1762 (2013). 23832500 10.1007/s00424-013-1315-z PMC3898376

[301] Sirianant, L., Ousingsawat, J., Wanitchakool, P., Schreiber, R. & Kunzelmann, K. Cellular volume regulation by anoctamin 6: Ca^2+^, phospholipase A2 and osmosensing. *Pflugers Arch.* **468**, 335–349 (2016). 26438191 10.1007/s00424-015-1739-8

[302] Xuan, Z., Wang, Y. & Xie, J. ANO6 promotes cell proliferation and invasion in glioma through regulating the ERK signaling pathway. *Oncotargets Ther.* **12**, 6721–6731 (2019). 10.2147/OTT.S211725 PMC6708391 31692479

[303] Yang, J. et al. PAC, an evolutionarily conserved membrane protein, is a proton-activated chloride channel. *Science* **364**, 395–399 (2019). 31023925 10.1126/science.aav9739 PMC7305803

[304] Ruan, Z., Osei-Owusu, J., Du, J., Qiu, Z. & Lu, W. Structures and pH-sensing mechanism of the proton-activated chloride channel. *Nature* **588**, 350–354 (2020). 33149300 10.1038/s41586-020-2875-7 PMC7773282

[305] Osei-Owusu, J. et al. Proton-activated chloride channel PAC regulates endosomal acidification and transferrin receptor-mediated endocytosis. *Cell Rep.* **34**, 108683 (2021). 33503418 10.1016/j.celrep.2020.108683 PMC7869721

[306] Xue, P. et al. Proton-activated chloride channel increases endplate porosity and pain in a mouse spine degeneration model. *J. Clin. Invest*. **134**, e168155 (2024). 10.1172/JCI168155 PMC11473161 39196784

[307] Pan, D. et al. Senescence of endplate osteoclasts induces sensory innervation and spinal pain. *Elife*. **12**, RP92889 (2024). 10.7554/eLife.92889 PMC11186630 38896465

[308] Palmieri, F. The mitochondrial transporter family (SLC25): physiological and pathological implications. *Pflugers Arch.* **447**, 689–709 (2004). 14598172 10.1007/s00424-003-1099-7

[309] Seifert, E. L., Ligeti, E., Mayr, J. A., Sondheimer, N. & Hajnoczky, G. The mitochondrial phosphate carrier: role in oxidative metabolism, calcium handling and mitochondrial disease. *Biochem. Biophys. Res. Commun.* **464**, 369–375 (2015). 26091567 10.1016/j.bbrc.2015.06.031 PMC8011645

[310] Boulet, A. et al. The mammalian phosphate carrier SLC25A3 is a mitochondrial copper transporter required for cytochrome c oxidase biogenesis. *J. Biol. Chem.* **293**, 1887–1896 (2018). 29237729 10.1074/jbc.RA117.000265 PMC5808751

[311] Wei, A., Liu, T. & O’Rourke, B. Dual effect of phosphate transport on mitochondrial Ca^2+^ dynamics. *J. Biol. Chem.* **290**, 16088–16098 (2015). 25963147 10.1074/jbc.M114.628446 PMC4481211

[312] Junkun, L. et al. Curcumin downregulates phosphate carrier and protects against doxorubicin-induced cardiomyocyte apoptosis. *Biomed. Res. Int.* **2016**, 1980763 (2016). 27127780 10.1155/2016/1980763 PMC4835619

[313] Kwong, J. Q. et al. Genetic deletion of the mitochondrial phosphate carrier desensitizes the mitochondrial permeability transition pore and causes cardiomyopathy. *Cell Death Differ.* **21**, 1209–1217 (2014). 24658400 10.1038/cdd.2014.36 PMC4085527

[314] Wang, P. et al. METTL14-mediated methylation of SLC25A3 mitigates mitochondrial damage in osteoblasts, leading to the improvement of osteoporosis. *Exp. Gerontol.* **194**, 112496 (2024). 38897394 10.1016/j.exger.2024.112496

[315] Zhou, T. et al. Single-cell transcriptomics in MI identify Slc25a4 as a new modulator of mitochondrial malfunction and apoptosis-associated cardiomyocyte subcluster. *Sci. Rep.* **14**, 9274 (2024). 38654053 10.1038/s41598-024-59975-8 PMC11039722

[316] Le, J., Chen, Y., Yang, W., Chen, L. & Ye, J. Metabolic basis of solute carrier transporters in treatment of type 2 diabetes mellitus. *Acta Pharm. Sin. B.* **14**, 437–454 (2024). 38322335 10.1016/j.apsb.2023.09.004 PMC10840401

[317] Zhu, S. et al. Slc25a5 regulates adipogenesis by modulating ERK signaling in OP9 cells. *Cell. Mol. Biol. Lett.* **27**, 11 (2022). 35109789 10.1186/s11658-022-00314-y PMC8903613

[318] Correction to Down-regulated lncRNA SLC25A5-AS1 facilitates cell growth and inhibits apoptosis via miR-19a-3p/PTEN/PI3K/AKT signalling pathway in gastric cancer. *J. Cell. Mol. Med*. **27**, 3215–3216 (2023). 10.1111/jcmm.17830 PMC10568661 37723981

[319] Zhou, X., Paredes, J. A., Krishnan, S., Curbo, S. & Karlsson, A. The mitochondrial carrier SLC25A10 regulates cancer cell growth. *Oncotarget* **6**, 9271–9283 (2015). 25797253 10.18632/oncotarget.3375 PMC4496216

[320] Huypens, P. et al. The dicarboxylate carrier plays a role in mitochondrial malate transport and in the regulation of glucose-stimulated insulin secretion from rat pancreatic beta cells. *Diabetologia* **54**, 135–145 (2011). 20949348 10.1007/s00125-010-1923-5

[321] Lin, Y. et al. The hyperglycemia-induced inflammatory response in adipocytes: the role of reactive oxygen species. *J. Biol. Chem.* **280**, 4617–4626 (2005). 15536073 10.1074/jbc.M411863200

[322] Wang, G. et al. SLC25A10 performs an oncogenic role in human osteosarcoma. *Oncol. Lett.* **20**, 2 (2020). 32774476 10.3892/ol.2020.11863 PMC7405602

[323] Dong, J., Zhong, J., Xu, Y., Ma, Y. & Duan, X. Research progress on structure, function and disease correlation of solute carrier family 4. *Sheng Li Xue Bao* **75**, 137–150 (2023). 36859843

[324] Choi, I. SLC4A transporters. *Curr. Top. Membr.* **70**, 77–103 (2012). 23177984 10.1016/B978-0-12-394316-3.00003-X PMC4768801

[325] Cai, L. et al. Dietary sodium enhances the expression of SLC4 family transporters, IRBIT, L-IRBIT, and PP1 in rat kidney: Insights into the molecular mechanism for renal sodium handling. *Front. Physiol.* **14**, 1154694 (2023). 37082243 10.3389/fphys.2023.1154694 PMC10111226

[326] Urso, K., Charles, J. F., Shull, G. E., Aliprantis, A. O. & Balestrieri, B. Anion exchanger 2 regulates dectin-1-dependent phagocytosis and killing of Candida albicans. *PloS One* **11**, e158893 (2016). 10.1371/journal.pone.0158893 PMC4938408 27391897

[327] Wu, J., Glimcher, L. H. & Aliprantis, A. O. HCO3-/Cl- anion exchanger SLC4A2 is required for proper osteoclast differentiation and function. *Proc. Natl. Acad. Sci. USA* **105**, 16934–16939 (2008). 18971331 10.1073/pnas.0808763105 PMC2579356

[328] Coury, F. et al. SLC4A2-mediated Cl^-^/HCO3^-^ exchange activity is essential for calpain-dependent regulation of the actin cytoskeleton in osteoclasts. *Proc. Natl. Acad. Sci. USA* **110**, 2163–2168 (2013). 23341620 10.1073/pnas.1206392110 PMC3568349

[329] Xue, J., Ikegawa, S. & Guo, L. SLC4A2, another gene involved in acid-base balancing machinery of osteoclasts, causes osteopetrosis. *Bone* **167**, 116603 (2023). 36343920 10.1016/j.bone.2022.116603

[330] Xue, J. et al. SLC4A2 deficiency causes a new type of osteopetrosis. *J. Bone. Miner. Res.* **37**, 226–235 (2022). 34668226 10.1002/jbmr.4462

[331] Josephsen, K. et al. Targeted disruption of the Cl^-^/HCO3^-^ exchanger Ae2 results in osteopetrosis in mice. *Proc. Natl. Acad. Sci. USA* **106**, 1638–1641 (2009). 19164575 10.1073/pnas.0811682106 PMC2635809

[332] Riihonen, R., Nielsen, S., Vaananen, H. K., Laitala-Leinonen, T. & Kwon, T. Degradation of hydroxyapatite in vivo and in vitro requires osteoclastic sodium-bicarbonate co-transporter NBCn1. *Matrix Biol.* **29**, 287–294 (2010). 20079835 10.1016/j.matbio.2010.01.003

[333] Bouyer, P. et al. Colony-stimulating factor-1 increases osteoclast intracellular pH and promotes survival via the electroneutral Na/HCO3 cotransporter NBCn1. *Endocrinology* **148**, 831–840 (2007). 17068143 10.1210/en.2006-0547

[334] Ali, E. S. et al. The mTORC1-SLC4A7 axis stimulates bicarbonate import to enhance de novo nucleotide synthesis. *Mol. Cell.* **82**, 3284–3298 (2022). 35772404 10.1016/j.molcel.2022.06.008 PMC9444906

[335] Galvan, A. & Fernandez, E. Eukaryotic nitrate and nitrite transporters. *Cell. Mol. Life Sci.* **58**, 225–233 (2001). 11289304 10.1007/PL00000850 PMC11146504

[336] Lundberg, J. O., Weitzberg, E., Cole, J. A. & Benjamin, N. Nitrate, bacteria and human health. *Nat. Rev. Microbiol.* **2**, 593–602 (2004). 15197394 10.1038/nrmicro929

[337] Wang, Y., Cheng, Y., Chen, K. & Tsay, Y. Nitrate transport, signaling, and use efficiency. *Annu. Rev. Plant Biol.* **69**, 85–122 (2018). 29570365 10.1146/annurev-arplant-042817-040056

[338] Kalyanaraman, H. et al. A novel, direct NO donor regulates osteoblast and osteoclast functions and increases bone mass in ovariectomized mice. *J. Bone. Miner. Res.* **32**, 46–59 (2017). 27391172 10.1002/jbmr.2909 PMC5199609

[339] Frommer, W. B., Hummel, S. & Rentsch, D. Cloning of an Arabidopsis histidine transporting protein related to nitrate and peptide transporters. *FEBS Lett.* **347**, 185–189 (1994). 8033999 10.1016/0014-5793(94)00533-8

[340] Quesada, A. et al. PCR-identification of a Nicotiana plumbaginifolia cDNA homologous to the high-affinity nitrate transporters of the crnA family. *Plant Mol. Biol.* **34**, 265–274 (1997). 9207842 10.1023/a:1005872816881

[341] Dong, S. S., Williams, J. P., Jordan, S. E., Cornwell, T. & Blair, H. C. Nitric oxide regulation of cGMP production in osteoclasts. *J. Cell. Biochem.* **73**, 478–487 (1999). 10733342

[342] Jeddi, S., Yousefzadeh, N., Kashfi, K. & Ghasemi, A. Role of nitric oxide in type 1 diabetes-induced osteoporosis. *Biochem. Pharmacol.* **197**, 114888 (2022). 34968494 10.1016/j.bcp.2021.114888

[343] Li, X., Augustine, A., Chen, S. & Fliegel, L. Stop codon polymorphisms in the human SLC9A1 gene disrupt or compromise Na^+^/H^+^ exchanger function. *PloS One* **11**, e162902 (2016). 10.1371/journal.pone.0162902 PMC5026351 27636896

[344] Fuster, D. G. & Alexander, R. T. Traditional and emerging roles for the SLC9 Na^+^/H^+^ exchangers. *Pflugers Arch.* **466**, 61–76 (2014). 24337822 10.1007/s00424-013-1408-8

[345] Biemesderfer, D., Reilly, R. F., Exner, M., Igarashi, P. & Aronson, P. S. Immunocytochemical characterization of Na^+^-H^+^ exchanger isoform NHE-1 in rabbit kidney. *Am. J. Physiol.* **263**, F833–F840 (1992). 1279986 10.1152/ajprenal.1992.263.5.F833

[346] Petrecca, K., Atanasiu, R., Grinstein, S., Orlowski, J. & Shrier, A. Subcellular localization of the Na^+^/H^+^ exchanger NHE1 in rat myocardium. *Am. J. Physiol.* **276**, H709–H717 (1999). 9950874 10.1152/ajpheart.1999.276.2.H709

[347] Amith, S. R. & Fliegel, L. Regulation of the Na^+^/H^+^ exchanger (NHE1) iN Breast Cancer Metastasis. *Cancer Res.* **73**, 1259–1264 (2013). 23393197 10.1158/0008-5472.CAN-12-4031

[348] Guissart, C. et al. Mutation of SLC9A1, encoding the major Na^+^/H^+^ exchanger, causes ataxia-deafness Lichtenstein-Knorr syndrome. *Hum. Mol. Genet.* **24**, 463–470 (2015). 25205112 10.1093/hmg/ddu461

[349] Liu, L., Schlesinger, P. H., Slack, N. M., Friedman, P. A. & Blair, H. C. High capacity Na^+^/H^+^ exchange activity in mineralizing osteoblasts. *J. Cell. Physiol.* **226**, 1702–1712 (2011). 21413028 10.1002/jcp.22501 PMC4458346

[350] Schnyder, D. et al. Deletion of the sodium/hydrogen exchanger 6 causes low bone volume in adult mice. *Bone* **153**, 116178 (2021). 34508879 10.1016/j.bone.2021.116178

[351] Lee, S. H. et al. NHE10, an osteoclast-specific member of the Na^+^/H^+^ exchanger family, regulates osteoclast differentiation and survival [corrected]. *Biochem. Biophys. Res. Commun.* **369**, 320–326 (2008). 18269914 10.1016/j.bbrc.2008.01.168

[352] Mine, Y. et al. Inhibition of RANKL-dependent cellular fusion in pre-osteoclasts by amiloride and an NHE10-specific monoclonal antibody. *Cell Biol. Int.* **39**, 696–709 (2015). 25612314 10.1002/cbin.10447

[353] Matsuoka, R. et al. Author Correction: Structure, mechanism and lipid-mediated remodeling of the mammalian Na^+^/H^+^ exchanger NHA2. *Nat. Struct. Mol. Biol.* **30**, 565 (2023). 37029209 10.1038/s41594-023-00970-4 PMC10113147

[354] Hofstetter, W., Siegrist, M., Simonin, A., Bonny, O. & Fuster, D. G. Sodium/hydrogen exchanger NHA2 in osteoclasts: subcellular localization and role in vitro and in vivo. *Bone* **47**, 331–340 (2010). 20441802 10.1016/j.bone.2010.04.605

[355] Battaglino, R. A. et al. NHA-oc/NHA2: a mitochondrial cation-proton antiporter selectively expressed in osteoclasts. *Bone* **42**, 180–192 (2008). 17988971 10.1016/j.bone.2007.09.046 PMC3593247

[356] Ha, B. G. et al. Proteomic profile of osteoclast membrane proteins: identification of Na^+^/H^+^ exchanger domain containing 2 and its role in osteoclast fusion. *Proteomics* **8**, 2625–2639 (2008). 18600791 10.1002/pmic.200701192

[357] Quednau, B. D., Nicoll, D. A. & Philipson, K. D. The sodium/calcium exchanger family-SLC8. *Pflugers Arch.* **447**, 543–548 (2004). 12734757 10.1007/s00424-003-1065-4

[358] Albano, G. et al. Increased bone resorption by osteoclast-specific deletion of the sodium/calcium exchanger isoform 1 (NCX1). *Pflugers Arch.* **469**, 225–233 (2017). 27942992 10.1007/s00424-016-1923-5

[359] Moonga, B. S. et al. Identification and characterization of a sodium/calcium exchanger, NCX-1, in osteoclasts and its role in bone resorption. *Biochem. Biophys. Res. Commun.* **283**, 770–775 (2001). 11350050 10.1006/bbrc.2001.4870

[360] Li, T. et al. NCX1 disturbs calcium homeostasis and promotes RANKL-induced osteoclast differentiation by regulating JNK/c-Fos/NFATc1 signaling pathway in multiple myeloma. *Clin. Exper. Med.* **23**, 1581–1596 (2023). 36251145 10.1007/s10238-022-00905-1 PMC10460717

[361] Gupta, A., Guo, X. L., Alvarez, U. M. & Hruska, K. A. Regulation of sodium-dependent phosphate transport in osteoclasts. *J. Clin. Invest.* **100**, 538–549 (1997). 9239400 10.1172/JCI119563 PMC508220

[362] Gupta, A. et al. Identification of the type II Na^+^-Pi cotransporter (Npt2) in the osteoclast and the skeletal phenotype of Npt2^−/−^ mice. *Bone* **29**, 467–476 (2001). 11704500 10.1016/s8756-3282(01)00601-9

[363] Reimer, R. J. & Edwards, R. H. Organic anion transport is the primary function of the SLC17/type I phosphate transporter family. *Pflugers Arch.* **447**, 629–635 (2004). 12811560 10.1007/s00424-003-1087-y

[364] Forster, I. C., Hernando, N., Biber, J. & Murer, H. Phosphate transporters of the SLC20 and SLC34 families. *Mol. Aspects Med.* **34**, 386–395 (2013). 23506879 10.1016/j.mam.2012.07.007

[365] Roman-Fernandez, A. et al. The phospholipid PI(3,4)P(2) is an apical identity determinant. *Nat. Commun.* **9**, 5041 (2018). 30487552 10.1038/s41467-018-07464-8 PMC6262019

[366] Gassama-Diagne, A. et al. Phosphatidylinositol-3,4,5-trisphosphate regulates the formation of the basolateral plasma membrane in epithelial cells. *Nat. Cell Biol.* **8**, 963–970 (2006). 16921364 10.1038/ncb1461

[367] Albano, G. et al. Sodium-dependent phosphate transporters in osteoclast differentiation and function. *PloS One* **10**, e125104 (2015). 10.1371/journal.pone.0125104 PMC4409223 25910236

[368] Belenky, M. A. et al. Cell-type-specific distribution of chloride transporters in the rat suprachiasmatic nucleus. *Neuroscience* **165**, 1519–1537 (2010). 19932740 10.1016/j.neuroscience.2009.11.040 PMC2815043

[369] Fujii, T., Fujita, K., Takeguchi, N. & Sakai, H. Function of K^+^-Cl^-^ cotransporters in the acid secretory mechanism of gastric parietal cells. *Biol. Pharm. Bull.* **34**, 810–812 (2011). 21628876 10.1248/bpb.34.810

[370] Brauer, M., Frei, E., Claes, L., Grissmer, S. & Jager, H. Influence of K-Cl cotransporter activity on activation of volume-sensitive Cl^-^ channels in human osteoblasts. *Am. J. Physiol. Cell Physiol.* **285**, C22–C30 (2003). 12637262 10.1152/ajpcell.00289.2002

[371] Jalali, R. et al. The role of Na:K:2Cl cotransporter 1 (NKCC1/SLC12A2) in dental epithelium during enamel formation in mice. *Front. Physiol.* **8**, 924 (2017). 29209227 10.3389/fphys.2017.00924 PMC5702478

[372] Kajiya, H., Okamoto, F., Li, J., Nakao, A. & Okabe, K. Expression of mouse osteoclast K-Cl Co-transporter-1 and its role during bone resorption. *J. Bone. Miner. Res.* **21**, 984–992 (2006). 16813519 10.1359/jbmr.060407

[373] Zhao, F. & Keating, A. F. Functional properties and genomics of glucose transporters. *Curr. Genom.* **8**, 113–128 (2007). 10.2174/138920207780368187 PMC2435356 18660845

[374] Mueckler, M. & Thorens, B. The SLC2 (GLUT) family of membrane transporters. *Mol. Aspects Med.* **34**, 121–138 (2013). 23506862 10.1016/j.mam.2012.07.001 PMC4104978

[375] Indo, Y. et al. Metabolic regulation of osteoclast differentiation and function. *J. Bone. Miner. Res.* **28**, 2392–2399 (2013). 23661628 10.1002/jbmr.1976

[376] Scalise, M., Pochini, L., Pingitore, P., Hedfalk, K. & Indiveri, C. Cysteine is not a substrate but a specific modulator of human ASCT2 (SLC1A5) transporter. *FEBS Lett.* **589**, 3617–3623 (2015). 26492990 10.1016/j.febslet.2015.10.011

[377] Li, B. et al. Both aerobic glycolysis and mitochondrial respiration are required for osteoclast differentiation. *FASEB J.* **34**, 11058–11067 (2020). 32627870 10.1096/fj.202000771R

[378] Bartoloni, L. & Antonarakis, S. E. The human sugar-phosphate/phosphate exchanger family SLC37. *Pflugers Arch.* **447**, 780–783 (2004). 12811562 10.1007/s00424-003-1105-0

[379] Kim, J. Y., Tillison, K., Zhou, S., Wu, Y. & Smas, C. M. The major facilitator superfamily member Slc37a2 is a novel macrophage-specific gene selectively expressed in obese white adipose tissue. *Am. J. Physiol.-Endocrinol. Metab.* **293**, E110–E120 (2007). 17356011 10.1152/ajpendo.00404.2006

[380] Pan, C. et al. SLC37A1 and SLC37A2 are phosphate-linked, glucose-6-phosphate antiporters. *PloS One* **6**, e23157 (2011). 21949678 10.1371/journal.pone.0023157 PMC3176764

[381] Ng, P. Y. et al. Sugar transporter Slc37a2 regulates bone metabolism in mice via a tubular lysosomal network in osteoclasts. *Nat. Commun.* **14**, 906 (2023). 36810735 10.1038/s41467-023-36484-2 PMC9945426

[382] Young, J. D., Yao, S. Y. M., Baldwin, J. M., Cass, C. E. & Baldwin, S. A. The human concentrative and equilibrative nucleoside transporter families, SLC28 and SLC29. *Mol. Aspects Med.* **34**, 529–547 (2013). 23506887 10.1016/j.mam.2012.05.007

[383] Huang, W., Zeng, X., Shi, Y. & Liu, M. Functional characterization of human equilibrative nucleoside transporter 1. *Protein Cell* **8**, 284–295 (2017). 27995448 10.1007/s13238-016-0350-x PMC5359181

[384] Katsuyama, S. et al. Identification of a novel compound that inhibits osteoclastogenesis by suppressing nucleoside transporters. *FEBS Lett.* **590**, 1152–1162 (2016). 27001232 10.1002/1873-3468.12146

[385] Vasko, B. et al. Inhibitor selectivity of CNTs and ENTs. *Xenobiotica* **49**, 840–851 (2019). 30022699 10.1080/00498254.2018.1501832

[386] Griffith, D. A. & Jarvis, S. M. Nucleoside and nucleobase transport systems of mammalian cells. *Biochim. Biophys. Acta* **1286**, 153–181 (1996). 8982282 10.1016/s0304-4157(96)00008-1

[387] He, W. & Cronstein, B. The roles of adenosine and adenosine receptors in bone remodeling. *Front. Biosci. (Elite Ed.)* **3**, 888–895 (2011). 21622100 10.2741/e297

[388] Hinton, D. J. et al. Aberrant bone density in aging mice lacking the adenosine transporter ENT1. *PloS One* **9**, e88818 (2014). 24586402 10.1371/journal.pone.0088818 PMC3929493

[389] Kanai, Y. & Hediger, M. A. The glutamate/neutral amino acid transporter family SLC1: molecular, physiological and pharmacological aspects. *Pflugers Arch.* **447**, 469–479 (2004). 14530974 10.1007/s00424-003-1146-4

[390] Broer, A., Rahimi, F. & Broer, S. Deletion of amino acid transporter ASCT2 (SLC1A5) reveals an essential role for transporters SNAT1 (SLC38A1) and SNAT2 (SLC38A2) to sustain glutaminolysis in cancer cells. *J. Biol. Chem.* **291**, 13194–13205 (2016). 27129276 10.1074/jbc.M115.700534 PMC4933233

[391] Yu, Y. et al. Glutamine metabolism regulates proliferation and lineage allocation in skeletal stem cells. *Cell Metab.* **29**, 966–978 (2019). 30773468 10.1016/j.cmet.2019.01.016 PMC7062112

[392] Soe, K. et al. Involvement of human endogenous retroviral syncytin-1 in human osteoclast fusion. *Bone* **48**, 837–846 (2011). 21111077 10.1016/j.bone.2010.11.011

[393] Mason, D. J. & Huggett, J. F. Glutamate transporters in bone. *J. Musculoskelet. Neuronal Interact.* **2**, 406–414 (2002). 15758408

[394] Morimoto, R. et al. Secretion of L-glutamate from osteoclasts through transcytosis. *EMBO J.* **25**, 4175–4186 (2006). 16957773 10.1038/sj.emboj.7601317 PMC1570443

[395] Peng, R. et al. IL-17 promotes osteoclast-induced bone loss by regulating glutamine-dependent energy metabolism. *Cell Death Dis.* **15**, 111 (2024). 38316760 10.1038/s41419-024-06475-2 PMC10844210

[396] Fotiadis, D., Kanai, Y. & Palacin, M. The SLC3 and SLC7 families of amino acid transporters. *Mol. Aspects Med.* **34**, 139–158 (2013). 23506863 10.1016/j.mam.2012.10.007

[397] Zhang, C. et al. SLC3A2 N-glycosylation and Golgi remodeling regulate SLC7A amino acid exchangers and stress mitigation. *J. Biol. Chem.* **299**, 105416 (2023). 37918808 10.1016/j.jbc.2023.105416 PMC10698284

[398] You, S., Han, X., Xu, Y. & Yao, Q. Research progress on the role of cationic amino acid transporter (CAT) family members in malignant tumors and immune microenvironment. *Amino Acids* **55**, 1213–1222 (2023). 37572157 10.1007/s00726-023-03313-1

[399] Verrey, F. et al. CATs and HATs: the SLC7 family of amino acid transporters. *Pflugers Arch.* **447**, 532–542 (2004). 14770310 10.1007/s00424-003-1086-z

[400] Saier, M. H. J., Yen, M. R., Noto, K., Tamang, D. G. & Elkan, C. The Transporter Classification Database: recent advances. *Nucleic Acids. Res.* **37**, D274–D278 (2009). 19022853 10.1093/nar/gkn862 PMC2686586

[401] Kondo, T., Ikeda, K. & Matsuo, K. Detection of osteoclastic cell-cell fusion through retroviral vector packaging. *Bone* **35**, 1120–1126 (2004). 15542037 10.1016/j.bone.2004.06.011

[402] Liao, Y. et al. Single-cell profiling of SLC family transporters: uncovering the role of SLC7A1 in osteosarcoma. *J. Transl. Med.* **23**, 103 (2025). 39844299 10.1186/s12967-025-06086-1 PMC11752724

[403] Kandasamy, P., Gyimesi, G., Kanai, Y. & Hediger, M. A. Amino acid transporters revisited: New views in health and disease. *Trends Biochem. Sci.* **43**, 752–789 (2018). 30177408 10.1016/j.tibs.2018.05.003

[404] Ozaki, K. et al. The L-type amino acid transporter LAT1 inhibits osteoclastogenesis and maintains bone homeostasis through the mTORC1 pathway. *Sci. Signal*. **12**, 589 (2019). 10.1126/scisignal.aaw3921 PMC7285885 31289211

[405] Rivas, C. I. et al. Vitamin C transporters. *J. Physiol. Biochem.* **64**, 357–375 (2008). 19391462 10.1007/BF03174092

[406] Eck, P. et al. Comparison of the genomic structure and variation in the two human sodium-dependent vitamin C transporters, SLC23A1 and SLC23A2. *Hum. Genet.* **115**, 285–294 (2004). 15316768 10.1007/s00439-004-1167-x

[407] Hie, M. & Tsukamoto, I. Vitamin C-deficiency stimulates osteoclastogenesis with an increase in RANK expression. *J. Nutr. Biochem.* **22**, 164–171 (2011). 20444587 10.1016/j.jnutbio.2010.01.002

[408] Ragab, A. A., Lavish, S. A., Banks, M. A., Goldberg, V. M. & Greenfield, E. M. Osteoclast differentiation requires ascorbic acid. *J. Bone. Miner. Res.* **13**, 970–977 (1998). 9626628 10.1359/jbmr.1998.13.6.970

[409] Wu, X., Itoh, N., Taniguchi, T., Nakanishi, T. & Tanaka, K. Requirement of calcium and phosphate ions in expression of sodium-dependent vitamin C transporter 2 and osteopontin in MC3T3-E1 osteoblastic cells. *Biochim. Biophys. Acta* **1641**, 65–70 (2003). 12788230 10.1016/s0167-4889(03)00065-x

[410] Xiong, J., Feng, J., Yuan, D., Zhou, J. & Miao, W. Tracing the structural evolution of eukaryotic ATP-binding cassette transporter superfamily. *Sci. Rep.* **5**, 16724 (2015). 26577702 10.1038/srep16724 PMC4649718

[411] Schaedler, T. A. et al. Structures and functions of mitochondrial ABC transporters. *Biochem. Soc. Trans.* **43**, 943–951 (2015). 26517908 10.1042/BST20150118

[412] Brodeur, G. M. Knowing your ABCCs: novel functions of ABCC transporters. *J. Natl. Cancer. Inst.* **103**, 1207–1208 (2011). 21799181 10.1093/jnci/djr277 PMC3156804

[413] Guidotti, G. ATP transport and ABC proteins. *Chem. Biol* **3**, 703–706 (1996). 8939684 10.1016/s1074-5521(96)90244-6

[414] Dean, M., Hamon, Y. & Chimini, G. The human ATP-binding cassette (ABC) transporter superfamily. *J. Lipid Res.* **42**, 1007–1017 (2001). 11441126

[415] Irie, A., Yamamoto, K., Miki, Y. & Murakami, M. Phosphatidylethanolamine dynamics are required for osteoclast fusion. *Sci. Rep.* **7**, 46715 (2017). 28436434 10.1038/srep46715 PMC5402267

[416] Huang, X. et al. HDL impairs osteoclastogenesis and induces osteoclast apoptosis via upregulation of ABCG1 expression. *Acta Biochim. Biophys. Sin.* **50**, 853–861 (2018). 30060101 10.1093/abbs/gmy081

[417] Mourskaia, A. A. et al. ABCC5 supports osteoclast formation and promotes breast cancer metastasis to bone. *Breast Cancer Res.* **14**, R149 (2012). 23174366 10.1186/bcr3361 PMC4053136

[418] Kong, Y. Y. et al. Activated T cells regulate bone loss and joint destruction in adjuvant arthritis through osteoprotegerin ligand. *Nature* **402**, 304–309 (1999). 10580503 10.1038/46303

[419] Dougall, W. C. et al. RANK is essential for osteoclast and lymph node development. *Genes. Dev.* **13**, 2412–2424 (1999). 10500098 10.1101/gad.13.18.2412 PMC317030

[420] Negishi-Koga, T. & Takayanagi, H. Ca^2+^-NFATc1 signaling is an essential axis of osteoclast differentiation. *Immunol. Rev.* **231**, 241–256 (2009). 19754901 10.1111/j.1600-065X.2009.00821.x

[421] Matsuo, K. et al. Nuclear factor of activated T-cells (NFAT) rescues osteoclastogenesis in precursors lacking c-Fos. *J. Biol. Chem.* **279**, 26475–26480 (2004). 15073183 10.1074/jbc.M313973200

[422] Takito, J., Inoue, S. & Nakamura, M. The sealing zone in osteoclasts: a self-organized structure on the bone. *Int. J. Mol. Sci*. **19**, 984 (2018). 10.3390/ijms19040984 PMC5979552 29587415

[423] Saltel, F., Chabadel, A., Bonnelye, E. & Jurdic, P. Actin cytoskeletal organisation in osteoclasts: a model to decipher transmigration and matrix degradation. *Eur. J. Cell Biol.* **87**, 459–468 (2008). 18294724 10.1016/j.ejcb.2008.01.001

[424] Nijenhuis, T., Hoenderop, J. G. J. & Bindels, R. J. M. TRPV5 and TRPV6 in Ca^2+^ (re)absorption: regulating Ca^2+^ entry at the gate. *Pflugers Arch.* **451**, 181–192 (2005). 16044309 10.1007/s00424-005-1430-6

[425] Neubert, P. et al. NCX1 represents an ionic Na+ sensing mechanism in macrophages. *PloS Biol.* **18**, e3000722 (2020). 32569301 10.1371/journal.pbio.3000722 PMC7307728

[426] Mousawi, F. et al. Chemical activation of the Piezo1 channel drives mesenchymal stem cell migration via inducing ATP release and activation of P2 receptor purinergic signaling. *Stem. Cells.* **38**, 410–421 (2020). 31746084 10.1002/stem.3114 PMC7064961

[427] Wacquier, B., Combettes, L., Van Nhieu, G. T. & Dupont, G. Interplay between intracellular Ca^2+^ oscillations and Ca^2+^-stimulated mitochondrial metabolism. *Sci. Rep.* **6**, 19316 (2016). 26776859 10.1038/srep19316 PMC4725975

[428] Kartner, N. & Manolson, M. F. Novel techniques in the development of osteoporosis drug therapy: the osteoclast ruffled-border vacuolar H^+^-ATPase as an emerging target. *Expert. Opin. Drug Discov.* **9**, 505–522 (2014). 24749538 10.1517/17460441.2014.902155

[429] Osteresch, C. et al. The binding site of the V-ATPase inhibitor apicularen is in the vicinity of those for bafilomycin and archazolid. *J. Biol. Chem.* **287**, 31866–31876 (2012). 22815478 10.1074/jbc.M112.372169 PMC3442520

[430] Price, P. A., June, H. H., Buckley, J. R. & Williamson, M. K. SB 242784, a selective inhibitor of the osteoclastic V-H^+^ATPase, inhibits arterial calcification in the rat. *Circ. Res.* **91**, 547–552 (2002). 12242274 10.1161/01.res.0000033987.22436.50

[431] Niikura, K., Takano, M. & Sawada, M. A novel inhibitor of vacuolar ATPase, FR167356, which can discriminate between osteoclast vacuolar ATPase and lysosomal vacuolar ATPase. *Br. J. Pharmacol.* **142**, 558–566 (2004). 15148249 10.1038/sj.bjp.0705812 PMC1574973

[432] Sorensen, M. G., Henriksen, K., Neutzsky-Wulff, A. V., Dziegiel, M. H. & Karsdal, M. A. Diphyllin, a novel and naturally potent V-ATPase inhibitor, abrogates acidification of the osteoclastic resorption lacunae and bone resorption. *J. Bone. Miner. Res.* **22**, 1640–1648 (2007). 17576165 10.1359/jbmr.070613

[433] Lebreton, S., Jaunbergs, J., Roth, M. G., Ferguson, D. A. & De Brabander, J. K. Evaluating the potential of vacuolar ATPase inhibitors as anticancer agents and multigram synthesis of the potent salicylihalamide analog saliphenylhalamide. *Bioorg. Med. Chem. Lett.* **18**, 5879–5883 (2008). 18657422 10.1016/j.bmcl.2008.07.003 PMC2593418

[434] Xie, X. et al. Salicylihalamide A inhibits the V0 sector of the V-ATPase through a mechanism distinct from bafilomycin A1. *J. Biol. Chem.* **279**, 19755–19763 (2004). 14998996 10.1074/jbc.M313796200

[435] Kartner, N. et al. Inhibition of osteoclast bone resorption by disrupting vacuolar H^+^-ATPase a3-B2 subunit interaction. *J. Biol. Chem.* **285**, 37476–37490 (2010). 20837476 10.1074/jbc.M110.123281 PMC2988353

[436] Shin, D., Kim, M., Lee, S., Kim, T. & Kim, S. Inhibitory effects of luteolin on titanium particle-induced osteolysis in a mouse model. *Acta Biomater.* **8**, 3524–3531 (2012). 22583904 10.1016/j.actbio.2012.05.002

[437] Kim, T. et al. The effects of luteolin on osteoclast differentiation, function in vitro and ovariectomy-induced bone loss. *J. Nutr. Biochem.* **22**, 8–15 (2011). 20233653 10.1016/j.jnutbio.2009.11.002

[438] Lee, J. et al. Inhibitory effect of luteolin on osteoclast differentiation and function. *Cytotechnology* **61**, 125–134 (2009). 20162352 10.1007/s10616-010-9253-5 PMC2825295

[439] Wang, Y. et al. Pharmacological targeting of vacuolar H^+^-ATPase via subunit V1G combats multidrug-resistant cancer. *Cell Chem. Biol.* **27**, 1359–1370 (2020). 32649904 10.1016/j.chembiol.2020.06.011

[440] Wang, S. et al. Glycolysis-mediated activation of v-ATPase by nicotinamide mononucleotide ameliorates lipid-induced cardiomyopathy by repressing the CD36-TLR4 axis. *Circ. Res.* **134**, 505–525 (2024). 38422177 10.1161/CIRCRESAHA.123.322910 PMC10906217

[441] Dai, T. et al. Unveiling Vacuolar H^+^-ATPase subunit a as the primary target of the pyridinylmethyl-benzamide fungicide, fluopicolide. *J. Agric. Food. Chem.* **72**, 1527–1538 (2024). 38193425 10.1021/acs.jafc.3c08485

[442] Zhang, X. et al. A key amino acid substitution of vacuolar-type H^+^-ATPases A subunit (VATP-A) confers selective toxicity of a potential botanical insecticide, periplocoside P (PSP), in Mythimna separata and Spodoptera exigua. *Insect. Biochem. Mol. Biol.* **179**, 104277 (2025). 39961394 10.1016/j.ibmb.2025.104277

[443] Song, M. et al. Elevated intracellular Ca^2+^ functions downstream of mitodysfunction to induce Wallerian-like degeneration and necroptosis in organophosphorus-induced delayed neuropathy. *Toxicology* **504**, 153812 (2024). 38653376 10.1016/j.tox.2024.153812

[444] Bai, J., Liu, F., Wu, L., Wang, Y. & Li, X. Attenuation of TRPV1 by AMG-517 after nerve injury promotes peripheral axonal regeneration in rats. *Mol. Pain.* **14**, 2070385906 (2018). 10.1177/1744806918777614 PMC6009083 29768956

[445] Gavva, N. R. et al. AMG 9810 [(E)-3-(4-t-butylphenyl)-N-(2,3-dihydrobenzo[4] dioxin-6-yl)acrylamide], a novel vanilloid receptor 1 (TRPV1) antagonist with antihyperalgesic properties. *J. Pharmacol. Exp. Ther.* **313**, 474–484 (2005). 15615864 10.1124/jpet.104.079855

[446] Dunne, O. M. et al. TRPV2 modulates mechanically Induced ATP Release from Human bronchial epithelial cells. *Respir. Res.* **25**, 188 (2024). 38678280 10.1186/s12931-024-02807-0 PMC11056070

[447] Murphy, T. V. et al. TRPV3 expression and vasodilator function in isolated uterine radial arteries from non-pregnant and pregnant rats. *Vasc. Pharmacol.* **83**, 66–77 (2016). 10.1016/j.vph.2016.04.004 27073026

[448] Vincent, F. et al. Identification and characterization of novel TRPV4 modulators. *Biochem. Biophys. Res. Commun.* **389**, 490–494 (2009). 19737537 10.1016/j.bbrc.2009.09.007

[449] Cunha, M. R. et al. Natural product inspired optimization of a selective TRPV6 calcium channel inhibitor. *RSC Med. Chem.* **11**, 1032–1040 (2020). 33479695 10.1039/d0md00145g PMC7513592

[450] Li, Q. et al. Transient receptor potential melastatin 7 aggravates necrotizing enterocolitis by promoting an inflammatory response in children. *Transl. Pediatr.* **11**, 2030–2039 (2022). 36643673 10.21037/tp-22-633 PMC9834944

[451] Lai, P. & Michelangeli, F. Bis(2-hydroxy-3-tert-butyl-5-methyl-phenyl)-methane (bis-phenol) is a potent and selective inhibitor of the secretory pathway Ca^2+^ ATPase (SPCA1). *Biochem. Biophys. Res. Commun.* **424**, 616–619 (2012). 22796571 10.1016/j.bbrc.2012.07.004

[452] Chang, L. et al. Role of oxidative phosphorylation in the antidepressant effects of arketamine via the vagus nerve-dependent spleen-brain axis. *Neurobiol. Dis.* **199**, 106573 (2024). 38901783 10.1016/j.nbd.2024.106573

[453] Yang, Y. et al. Phoenixin 20 promotes neuronal mitochondrial biogenesis via CREB-PGC-1alpha pathway. *J. Mol. Histol.* **51**, 173–181 (2020). 32236796 10.1007/s10735-020-09867-8

[454] Jiang, H. et al. Target the human alanine/serine/cysteine transporter 2(ASCT2): achievement and future for novel cancer therapy. *Pharmacol. Res.* **158**, 104844 (2020). 32438035 10.1016/j.phrs.2020.104844

[455] Broer, A., Fairweather, S. & Broer, S. Disruption of amino acid homeostasis by novel ASCT2 inhibitors involves multiple targets. *Front. Pharmacol.* **9**, 785 (2018). 30072900 10.3389/fphar.2018.00785 PMC6060247

[456] Kurth, I. et al. Therapeutic targeting of SLC6A8 creatine transporter suppresses colon cancer progression and modulates human creatine levels. *Sci. Adv.* **7**, eabi7511 (2021). 34613776 10.1126/sciadv.abi7511 PMC8494442

[457] Montalbetti, N., Simonin, A., Dalghi, M. G., Kovacs, G. & Hediger, M. A. Development and validation of a fast and homogeneous cell-based fluorescence screening assay for divalent metal transporter 1 (DMT1/SLC11A2) using the FLIPR Tetra. *J. Biomol. Screen* **19**, 900–908 (2014). 24505080 10.1177/1087057114521663

[458] Fujii, T. et al. Functional association between K^+^-Cl^-^ cotransporter-4 and H^+^, K^+^-ATPase in the apical canalicular membrane of gastric parietal cells. *J. Biol. Chem.* **284**, 619–629 (2009). 18984587 10.1074/jbc.M806562200

[459] Cao, X., Lenk, G. M., Mikusevic, V., Mindell, J. A. & Meisler, M. H. The chloride antiporter CLCN7 is a modifier of lysosome dysfunction in FIG4 and VAC14 mutants. *PloS Genet.* **19**, e1010800 (2023). 37363915 10.1371/journal.pgen.1010800 PMC10328317

[460] Olotu, F. et al. Structure-based discovery and in vitro validation of inhibitors of chloride intracellular channel 4 protein. *Comp. Struct. Biotechnol. J.* **21**, 688–701 (2023). 10.1016/j.csbj.2022.12.040 PMC9826898 36659928

[461] Stewart, A. K., Kurschat, C. E., Vaughan-Jones, R. D., Shmukler, B. E. & Alper, S. L. Acute regulation of mouse AE2 anion exchanger requires isoform-specific amino acid residues from most of the transmembrane domain. *J. Physiol.* **584**, 59–73 (2007). 17690150 10.1113/jphysiol.2007.136119 PMC2277056

[462] Keiter, S. et al. Does perfluorooctane sulfonate (PFOS) act as chemosensitizer in zebrafish embryos? *Sci. Total Environ.* **548-549**, 317–324 (2016). 26803730 10.1016/j.scitotenv.2015.12.089

[463] Vagiannis, D. et al. Alisertib shows negligible potential for perpetrating pharmacokinetic drug-drug interactions on ABCB1, ABCG2 and cytochromes P450, but acts as dual-activity resistance modulator through the inhibition of ABCC1 transporter. *Toxicol. Appl. Pharmacol.* **434**, 115823 (2022). 34896433 10.1016/j.taap.2021.115823

[464] Tong, S. et al. Restoration of miR-299-3p promotes macrophage phagocytosis and suppresses malignant phenotypes in breast cancer carcinogenesis via dual-targeting CD47 and ABCE1. *Int. Immunopharmacol.* **130**, 111708 (2024). 38394889 10.1016/j.intimp.2024.111708

[465] Pafumi, I. et al. Naringenin impairs two-pore channel 2 activity and inhibits VEGF-induced angiogenesis. *Sci. Rep.* **7**, 5121 (2017). 28698624 10.1038/s41598-017-04974-1 PMC5505983

[466] Chassagnon, I. R. et al. Potent neuroprotection after stroke afforded by a double-knot spider-venom peptide that inhibits acid-sensing ion channel 1a. *Proc. Natl. Acad. Sci. USA* **114**, 3750–3755 (2017). 28320941 10.1073/pnas.1614728114 PMC5389327

[467] Wareham, K., Vial, C., Wykes, R. C. E., Bradding, P. & Seward, E. P. Functional evidence for the expression of P2X1, P2X4 and P2X7 receptors in human lung mast cells. *Br. J. Pharmacol.* **157**, 1215–1224 (2009). 19552691 10.1111/j.1476-5381.2009.00287.x PMC2743840

[468] Huang, H. et al. The potential of the P2X7 receptor as a therapeutic target in a sub-chronic PCP-induced rodent model of schizophrenia. *J. Chem. Neuroanat.* **116**, 101993 (2021). 34147620 10.1016/j.jchemneu.2021.101993

[469] Olov, N. et al. Using Nanoscale Passports To Understand and Unlock Ion Channels as Gatekeepers of the Cell. *Acs Nano.* **18**, 22709–22733 (2024). 39136685 10.1021/acsnano.4c05654

[470] Zhou, R., Fu, W., Vasylyev, D., Waxman, S. G. & Liu, C. Ion channels in osteoarthritis: emerging roles and potential targets. *Nat. Rev. Rheumatol.* **20**, 545–564 (2024). 39122910 10.1038/s41584-024-01146-0 PMC12374755

[471] DeJulius, C. R. et al. Engineering approaches for RNA-based and cell-based osteoarthritis therapies. *Nat. Rev. Rheumatol.* **20**, 81–100 (2024). 38253889 10.1038/s41584-023-01067-4 PMC11129836

[472] Jackson, T. C. et al. PPAR alpha agonists improve renal preservation in kidneys subjected to chronic in vitro perfusion: interaction with mannitol. *Transpl. Int.* **20**, 277–290 (2007). 17291221 10.1111/j.1432-2277.2006.00431.x

[473] Hong, J. H. et al. Markers of squamous cell carcinoma in sarco/endoplasmic reticulum Ca^2+^ ATPase 2 heterozygote mice keratinocytes. *Prog. Biophys. Mol. Biol.* **103**, 81–87 (2010). 19840814 10.1016/j.pbiomolbio.2009.10.005

[474] Hynes, D. & Harvey, B. J. Dexamethasone reduces airway epithelial Cl^-^ secretion by rapid non-genomic inhibition of KCNQ1, KCNN4 and KATP K^+^ channels. *Steroids* **151**, 108459 (2019). 31330137 10.1016/j.steroids.2019.108459

[475] Bobi, J. et al. Kv1.3 blockade inhibits proliferation of vascular smooth muscle cells in vitro and intimal hyperplasia in vivo. *Transl. Res.* **224**, 40–54 (2020). 32522668 10.1016/j.trsl.2020.06.002

[476] Rodriguez-Prados, M. et al. MICU1 controls the sensitivity of the mitochondrial Ca^2+^ uniporter to activators and inhibitors. *Cell Chem. Biol.* **30**, 606–617 (2023). 37244260 10.1016/j.chembiol.2023.05.002 PMC10370359

